# Predictors and pathways of language and motor development in four prospective cohorts of young children in Ghana, Malawi, and Burkina Faso

**DOI:** 10.1111/jcpp.12751

**Published:** 2017-05-23

**Authors:** Elizabeth L. Prado, Souheila Abbeddou, Seth Adu‐Afarwuah, Mary Arimond, Per Ashorn, Ulla Ashorn, Jaden Bendabenda, Kenneth H. Brown, Sonja Y. Hess, Emma Kortekangas, Anna Lartey, Kenneth Maleta, Brietta M. Oaks, Eugenia Ocansey, Harriet Okronipa, Jean Bosco Ouédraogo, Anna Pulakka, Jérôme W. Somé, Christine P. Stewart, Robert C. Stewart, Stephen A. Vosti, Elizabeth Yakes Jimenez, Kathryn G. Dewey

**Affiliations:** ^1^ Department of Nutrition University of California Davis Davis CA USA; ^2^ Department of Nutrition and Food Science University of Ghana Legon Accra Ghana; ^3^ Center for Child Health Research School of Medicine and Tampere University Hospital University of Tampere Tampere Finland; ^4^ Department of Paediatrics Tampere University Hospital Tampere Finland; ^5^ School of Public Health and Family Medicine University of Malawi College of Medicine Blantyre Malawi; ^6^ Bill & Melinda Gates Foundation Seattle WA USA; ^7^ Institut de Recherche en Sciences de la Santé/DRO Bobo‐Dioulasso Burkina Faso; ^8^ Department of Public Health University of Turku and Turku University Hospital Turku Finland; ^9^ Divison of Psychiatry University of Edinburgh Edinburgh UK; ^10^ Department of Agricultural and Resource Economics University of California Davis Davis CA USA; ^11^ Center for Education Policy Research University of New Mexico Albuquerque NM USA

**Keywords:** Language development, motor development, risk factors, low‐ and middle‐income countries, stimulation, nutrition, growth, lipid‐based nutrient supplements, iLiNS Project

## Abstract

**Background:**

Previous reviews have identified 44 risk factors for poor early child development (ECD) in low‐ and middle‐income countries. Further understanding of their relative influence and pathways is needed to inform the design of interventions targeting ECD.

**Methods:**

We conducted path analyses of factors associated with 18‐month language and motor development in four prospective cohorts of children who participated in trials conducted as part of the International Lipid‐Based Nutrient Supplements (iLiNS) Project in Ghana (*n* = 1,023), Malawi (*n* = 675 and 1,385), and Burkina Faso (*n* = 1,122). In two cohorts, women were enrolled during pregnancy. In two cohorts, infants were enrolled at 6 or 9 months. In multiple linear regression and structural equation models (*SEM*), we examined 22 out of 44 factors identified in previous reviews, plus 12 additional factors expected to be associated with ECD.

**Results:**

Out of 42 indicators of the 34 factors examined, 6 were associated with 18‐month language and/or motor development in 3 or 4 cohorts: child linear and ponderal growth, variety of play materials, activities with caregivers, dietary diversity, and child hemoglobin/iron status. Factors that were not associated with child development were indicators of maternal Hb/iron status, maternal illness and inflammation during pregnancy, maternal perceived stress and depression, exclusive breastfeeding during 6 months postpartum, and child diarrhea, fever, malaria, and acute respiratory infections. Associations between socioeconomic status and language development were consistently mediated to a greater extent by caregiving practices than by maternal or child biomedical conditions, while this pattern for motor development was not consistent across cohorts.

**Conclusions:**

Key elements of interventions to ensure quality ECD are likely to be promotion of caregiver activities with children, a variety of play materials, and a diverse diet, and prevention of faltering in linear and ponderal growth and improvement in child hemoglobin/iron status.

## Introduction

In high‐income countries, early life experiences, such as intrauterine growth restriction, preterm birth, and early childhood education have been shown to have long‐term cognitive consequences (Fox, Levitt, & Nelson, 2010). Children in low and middle‐income countries (LMICs) bear a greater burden of early life risk factors for poor early child development (ECD) (Walker et al., 2011). Four systematic and qualitative reviews have identified 44 modifiable risk factors for poor ECD in LMICs (Jensen et al., 2015; Wachs & Rahman, 2013; Walker et al., 2007, 2011). Further understanding of the relative influence of these factors on child development and their pathways is needed to inform the design of interventions.

Distal and proximal risk factors, which cooccur in the context of environmental adversity, may influence child development through multiple pathways. Environmental factors, such as limited household resources and poor quality water and sanitation, may affect child development directly, or through affecting maternal or child health or nutritional status, or through influencing caregiving practices. Child health and nutritional status may also influence child development directly or through children's physical activity or through caregiver behavior. This latter pathway is based on the functional isolation hypothesis, which posits that children who are malnourished, often ill, or small for their age are less active exploring their environment, and/or receive less developmental stimulation from caregivers, both of which can affect child development (Brown & Pollitt, 1996). This pathway also reflects Sameroff's transactional model, in which the child's effect on the environment is as important as the environment's effect on the child (Sameroff, 2009). Environmental adversity may also cause high levels of chronic stress, and therefore chronic exposure to elevated stress hormones, which can affect brain development (Lupien, McEwen, Gunnar, & Heim, 2009).

Eighteen previous studies reported mediation or pathway analyses of factors associated with child development in low‐ and middle‐income countries (for details, see supplementary material available online only). Out of these 18 studies, the maximum number of factors examined was seven, only one study included maternal information gathered during pregnancy, and none examined the relative direct and indirect associations of multiple environmental, maternal, caregiving, and child factors with ECD. We examined 22 out of the 44 factors identified in previous reviews, plus 12 additional factors expected to be associated with ECD (Table S1). We analyzed 42 indicators of these 34 factors in four cohorts of children (*n = *4,205) who participated in the International Lipid‐Based Nutrient Supplements (iLiNS) Project in Ghana, Malawi, and Burkina Faso. The first objective was to identify the factors associated with 18‐month language and motor development in each iLiNS cohort. The second objective was to identify the pathways through which these factors operate. Harmonization of many of the variables across cohorts allowed replication of results in different contexts.

**Figure 1:**
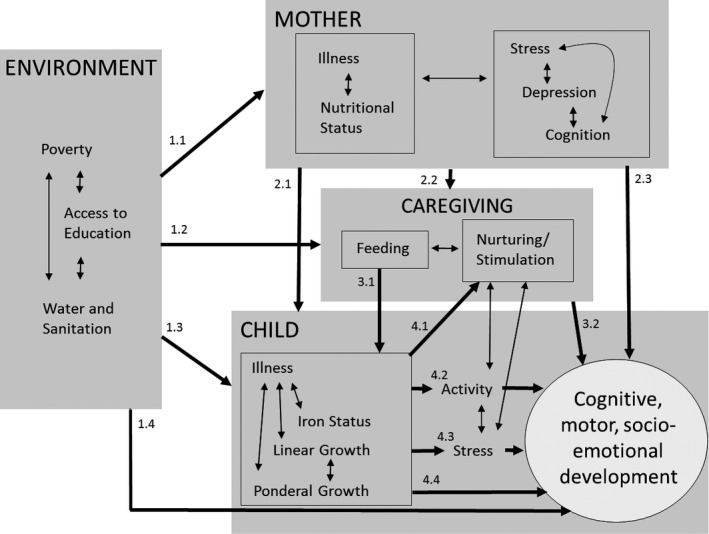
Conceptual model

Based on several previous theoretical path models (Brown & Pollitt, 1996; Engle, Menon, Garrett, & Slack, 1997; Walker et al., 2007), we developed a conceptual path model of potential influences on child development (Figure 1). We tested the following pathways, which correspond to the labels of the arrows in Figure 1. Pathway (1): Socioeconomic disparities and other environmental effects on child development may be mediated by (1.1) maternal factors, (1.2) caregiving practices, (1.3) child factors, or (1.4) may directly affect child development. Pathway (2): Maternal effects on child development may be mediated by (2.1) child factors, (2.2) caregiving practices, or (2.3) may directly affect child development. Pathway (3.1): Effects of infant feeding practices on child development may be mediated by child nutritional status. Pathway (3.2): Caregiving practices may directly affect child development. Pathway (4.1): Children who are anemic, small for their age, or often ill may elicit different stimulation from caregivers, which may mediate effects on child development. Pathway (4.2): Children who are anemic, small for their age, or often ill may be less active, resulting in less exploration of the environment, which may mediate effects on child development. Pathway (4.3): Children who are stunted or anemic may have higher basal cortisol, which may affect child development. Pathway (4.4): Child illness and nutritional status may directly affect child development.

## Methods

### iLiNS Project Trial Designs

In the iLiNS‐DYAD‐G trial in Ghana (*n = *1,320) and the iLiNS‐DYAD‐M trial in Malawi (*n = *869) pregnant women were enrolled before 20 weeks of gestation. In the iLiNS‐DOSE trial in Malawi (*n *=* *1,932) and iLiNS‐ZINC trial in Burkina Faso (*n *=* *3,220) infants were enrolled at age 6 and 9 months, respectively. All participants were assigned to receive various doses and formulations of lipid‐based nutrient supplements (LNS), or to control groups until age 18 months, when child development was assessed (Adu‐Afarwuah et al., 2015; Ashorn et al., 2015; Hess et al., 2015; Maleta et al., 2015). The effects of the interventions on 18‐month child development differed across trials, with positive effects in Burkina Faso (Prado, Abbeddou, Yakes Jimenez et al., 2016), but not in Ghana (Prado, Adu‐Afarwuah et al., 2016) or Malawi (Prado, Maleta et al., 2016; Prado, Phuka et al., 2016). Ethical approval for the study procedures was obtained from the Institutional Review Board of the University of California Davis or the Ethics Committee at Tampere University, Finland as well as local institutions in each country. All participants provided written informed consent. For further information, see Appendix S1 for extended supplementary methods.

### Participants

In the path analyses reported here, we included all children who participated in developmental assessment at age 18 months, comprising 1,023 children in iLiNS‐DYAD‐G (Cohort A), 675 in iLiNS‐DYAD‐M (Cohort B), 1,385 in iLiNS‐DOSE (Cohort C), and a random subsample of 1,122 children in iLiNS‐ZINC (Cohort D). The reasons for loss to follow‐up and the baseline characteristics of the developmental samples compared to those who enrolled but were not assessed have been published previously (Prado, Abbeddou, Yakes Jimenez et al., 2016; Prado, Adu‐Afarwuah et al., 2016; Prado, Maleta et al., 2016; Prado, Phuka et al., 2016). For the latter, Cohort A was the only cohort in which no significant differences were found; however, in cohorts C and D, the absolute differences were small (0.1 *SD*/2 to 6 percentage points), thus, they are likely of little practical significance. In Cohort B, enrolled mothers whose children did not participate had higher BMI and household assets, were significantly younger, and a greater proportion were primiparous.

### Procedure

**Figure 2:**
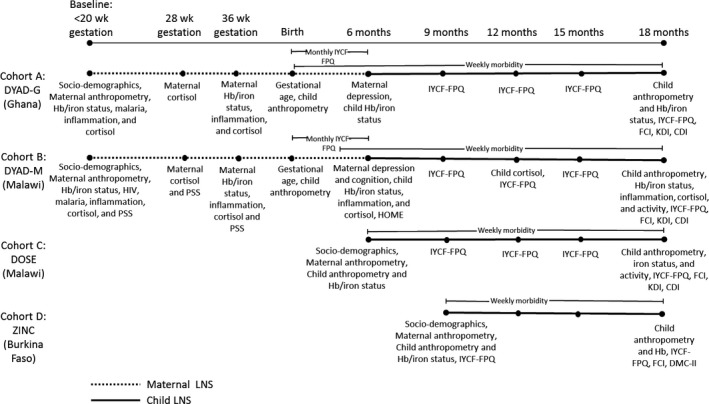
Data collection timeline in each cohort. Abbreviations: CDI: communicative development inventory, IYCF‐FPQ: infant and young child feeding—feeding practices questionnaire, DMC: developmental milestones checklist, FCI: family care indicators, Hb: hemoglobin, HOME, home observation for the measurement of the environment inventory, KDI: Kilifi developmental inventory, LNS: lipid‐based nutrient supplements, PSS: perceived stress scale

Detailed reports of the data collection procedures in each trial have been published elsewhere; therefore, we summarize the procedure for collection of variables that were used in the analyses presented here. Table S2 presents further details of the data collection procedures and variable definitions. Figure 2 shows the data collection schedule for each variable in each cohort.

Sociodemographic information was gathered at enrolment. Maternal and/or child anthropometry was measured at multiple time points. Capillary or venous blood samples were collected from mothers and/or children at multiple time points for the assessment of (a) malaria using a rapid diagnostic test, (b) biomarkers of hemoglobin/iron status, including hemoglobin (Hb) concentration (g/dl), zinc protoporphyrin (ZPP) concentration (μmol/mol heme), and soluble transferrin receptor (sTfR, mg/l), and (c) biomarkers of inflammation, including C‐reactive protein (CRP; mg/l) and alpha‐1 glycoprotein (AGP; g/l) concentrations. In ZINC and DYAD‐G, known HIV infection was an exclusion criterion, but HIV was not tested, therefore HIV status of women who enrolled was unknown. In DOSE, HIV status was also unknown. In DYAD‐M, women were tested for HIV at enrollment.

Maternal and/or child saliva samples in DYAD‐M and DYAD‐G were collected at several time points for the measurement of cortisol concentration (nmol/l) and maternal self‐reported stress was measured in DYAD‐M at multiple time points using the Perceived Stress Scale (Oaks et al., 2016; C. P. Stewart et al., 2015). Mothers were interviewed regarding depressive symptoms at 6 months postpartum in DYAD‐M using a locally validated adaptation of the Self‐Reporting Questionnaire (SRQ) and in DYAD‐G with the Edinburgh Post‐natal Depression Scale (EPDS) (R. C. Stewart et al., 2009). In DYAD‐M, at 6 months postpartum, maternal cognition was assessed using digit span forward and backward, verbal fluency, mental rotation, and functional health literacy tests (Prado, Ullman, Muadz, Alcock, & Shankar, 2012).

Children were visited weekly for morbidity surveillance. At these visits, caregivers were asked whether the child experienced any illness symptoms, including fever, diarrhea, vomiting, cough, nasal discharge, or respiratory distress during the past 7 days and/or data collectors measured the child's auricular temperature. Prevalence and/or incidence of diarrhea, fever, malaria, and/or acute respiratory infection were calculated (Table S2). In DOSE and DYAD‐M, physical activity at age 18 months was measured over 1 week with the hip‐worn ActiGraph GT3X+ accelerometer (Pensacola, FL, USA) (Pulakka et al., 2015).

Infant feeding practices were assessed at multiple time points through qualitative 24‐hr and/or 7‐day dietary recall questionnaires (Arimond et al., 2016). In DYAD‐M, nurturing and stimulation were measured at 6 months postpartum using the Home Observation for the Measurement of the Environment (HOME) Inventory (Caldwell & Bradley, 2003). In all four trials, developmental stimulation was measured at age 18 months using the Family Care Indicators (FCI) interview (Hamadani et al., 2010). The mother was interviewed with regard to the variety of play materials and activities in which adults engaged with the child in the past 3 days.

### Developmental assessment in Burkina Faso

In ZINC, we assessed motor and language development at age 18 months using the Developmental Milestones Checklist‐II (DMC‐II) (Prado et al., 2014). This tool evaluates motor and language development through both interviewing the caregiver and observing the child.

### Developmental assessment in Malawi and Ghana

In the DOSE and DYAD trials, we assessed motor development by the Kilifi Developmental Inventory (KDI), which is a direct assessment of the child (Abubakar, Holding, van Baar, Newton, & van de Vijver, 2008). Language development was assessed using a 100‐word vocabulary checklist by maternal interview based on the MacArthur‐Bates Communicative Development Inventory (Fenson et al., 2007). We developed the checklist in the local languages through interviews with caregivers and pilot testing (Prado, Adu‐Afarwuah et al., 2016; Prado, Maleta et al., 2016). The KDI and vocabulary scores showed high inter‐rater agreement and moderate to high test–retest reliability in all trials (Table S3).

### Analysis

We calculated *z*‐scores on all continuous variables so that the coefficients can be interpreted as the change in 1 *SD* of the dependent variable with every 1 *SD* change in the independent variable. For further details, see Appendix S1: Supplementary Methods.

#### Step 1: Variable selection

First, we examined independent associations between each predictor and each developmental score and dropped any that were not associated at *p *<* *.05. Second, we examined bivariate associations between predictors to check for collinearity. If two variables were highly collinear (*r *>* *.6), we dropped the one that was less strongly associated with the developmental score. Third, we examined six multivariate models and dropped any variables that were not associated at *p *<* *.05. The six multivariate models were: environmental factors; maternal factors; caregiving factors; child morbidity and nutritional status; child physical activity; and rating of the child's behavior during assessment.

#### Step 2: Path selection

We examined the association between each pair of variables on each pathway in Figure 1, to determine which variables were potential mediators. Unidirectional arrows represent pathways tested. Bidirectional arrows represent associations that were not modeled in the path analysis, except to check for collinearity. If any variables were not associated at *p *<* *.05, we dropped that pathway. All analyses up to this point were conducted using SAS version 9.4 (SAS Institute, Cary, NC). Next, for each independent variable with potential mediators, we tested the multiple mediation model using Stata version 14.1 (StataCorp, College Station, TX) binary mediation program including all potential mediators together. For each potential mediator for which the indirect effect was significant, we included the mediation pathway in the overall path model.

Finally, we ran the final path model using the sem command in Stata with the mlmv option to estimate the model on the full dataset using maximum likelihood estimation for missing values. All models with language or motor score as the dependent variable included four covariates: trial group, child sex and age at developmental assessment, and developmental data collector. For language scores, we also included whether the child was exposed to more than one language. Since the intervention affected developmental scores in ZINC only, we additionally examined the potential mediators of intervention versus control group in this cohort. We report *p*‐values uncorrected and corrected for multiple comparisons using the Benjamini–Hochberg correction (Benjamini & Hochberg, 1995), which is recommended for controlling the false discovery rate in *SEM* (Cribbie, 2007). We applied the correction separately for each model defined by one outcome in one cohort. For further details, see Appendix S1: Supplemental Methods.

## Results

Summary statistics for all independent variables are presented in Table S4. The variable selection results are shown in Tables S5–S8. The pathway selection results are shown in Tables S9–S12.

Among environmental variables, language scores were positively associated with higher household assets (DYAD‐G), households located further from the market (ZINC), an improved water source (ZINC), or higher paternal education (DYAD‐M, DOSE). Motor scores were associated with higher household assets (ZINC), households located closer to the market (DOSE), an improved water source (DOSE), or higher paternal education (DOSE).

Of the maternal variables, we observed higher language scores among children of mothers who were older (DYAD‐G), taller (DYAD‐M, ZINC), or had higher cognitive scores (DYAD‐M). Higher motor scores were associated with maternal height (DOSE, ZINC), higher BMI (ZINC), lower stress (basal cortisol) at 36 weeks gestation (DYAD‐M), higher health literacy (DYAD‐M), or inflammation (higher CRP) at <20 weeks gestation (DYAD‐G). This last association was in the opposite direction than expected.

Among the caregiving variables, we observed higher language scores among children who were fed more frequently (ZINC), had greater dietary diversity (DYAD‐G, DOSE, ZINC), and received higher quality stimulation from the home environment, as indicated by 6‐month HOME scores (DYAD‐M), 18‐month variety of play materials (all four cohorts), or 18‐month activities with caregivers (DYAD‐G, DOSE, ZINC). Higher motor scores were associated with greater dietary diversity (ZINC), and higher developmental stimulation, as indicated by 18‐month variety of play materials (DYAD‐M, DOSE, ZINC) and activities with caregivers (DYAD‐G, DOSE, ZINC).

Of the child variables, higher language scores were associated with having older siblings (DYAD‐G, DYAD‐M), LAZ in early infancy (birth/6/9 months; DYAD‐G, DOSE, ZINC), greater linear growth in later infancy (birth/6/9 to 18 months; DYAD‐G, DOSE, ZINC), WAZ at 9 months (ZINC), or higher Hb/iron status, as indicated by Hb at 6 months (DYAD‐G, DYAD‐M), change in Hb from 6 to 18 months (DYAD‐G, DYAD‐M), or ZPP at 9 months (ZINC). Motor scores were associated with LAZ in early infancy (birth/6/9 months; all four cohorts), change in LAZ in later infancy (birth/6/9 to 18 months; all four cohorts), ponderal growth status in early infancy (birth/6/9 months; DYAD‐G, DOSE, ZINC), ponderal growth in later infancy (birth/6 to 18 months; DYAD‐G, DOSE), inflammation (lower AGP) at 18 months (DYAD‐M), stress (lower basal cortisol) at 18 months (DYAD‐M), physical activity at 18 months (DOSE), or Hb/iron status as indicated by Hb at 9 months (ZINC), change in Hb 9 to 18 months (ZINC), ZPP at 6/9 months (DOSE and ZINC), or change in ZPP from 6 to 18 months (DOSE). Positive behavior during the KDI assessment also strongly predicted motor scores and exposure to multiple languages was positively associated with language scores.

**Figure 3:**
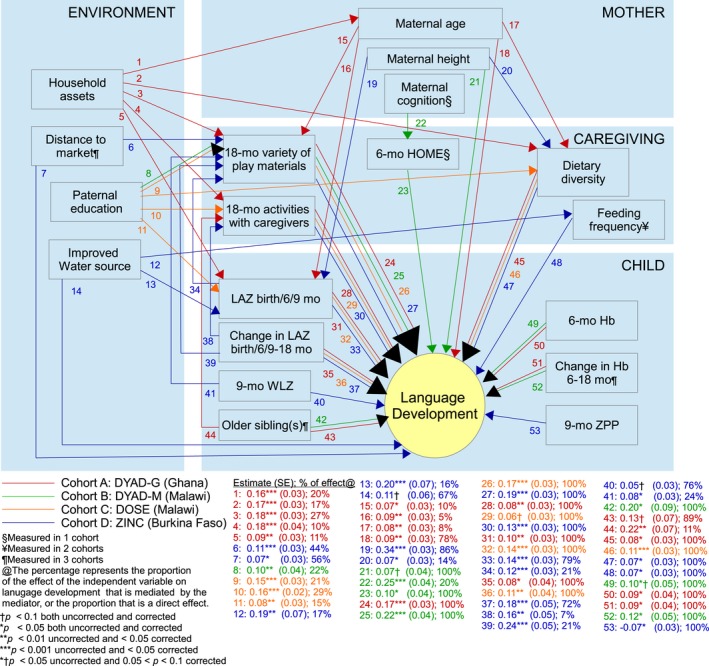
Path diagram of language development in four iLiNS cohorts

**Figure 4:**
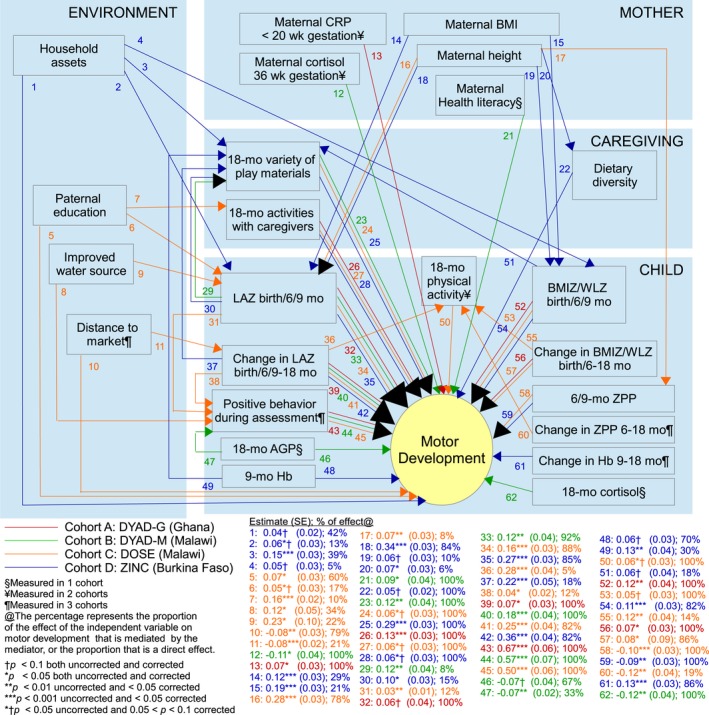
Path diagram of motor development in four iLiNS cohorts

The final models for language development accounted for 18% of the variance in language scores in DYAD‐G, 14% in DYAD‐M, 14% in DOSE, and 25% in ZINC. The final models for motor development accounted for 21% of the variance in motor scores in DYAD‐G, 36% in DYAD‐M, 31% in DOSE, and 36% in ZINC. Figures 3 and 4 present the coefficients of the direct and indirect effects that were significant at *p *<* *.1 in the final *SEM*. All coefficients are presented in Tables S13 and S14.

Regarding pathway (1), the extent to which socioeconomic disparities in language development were mediated by other factors varied across cohorts, from 22% to 85% (Figure S1). Associations between socioeconomic status (SES) and language scores were largely mediated by caregiving practices (22%–76%) rather than by maternal or child factors (0%–20%). For language, maternal age was the only maternal factor that was a mediator (DYAD‐G), and LAZ in early infancy (birth/6/9 months) was the only child factor that was a mediator (DYAD‐G, DOSE, ZINC), while four caregiving factors were mediators of SES (all four cohorts). For motor development, socioeconomic disparities were found in two cohorts and 21%–86% of these associations were mediated by other factors. In ZINC, these associations between SES and motor scores were mediated to a greater extent by caregiving practices (39%) than child factors (18%), while in DOSE they were mediated to a greater extent by child factors (21%–56%) than caregiving practices (0%–10%).

Regarding pathway (2), the extent to which associations between maternal nutritional status (height and BMI) and language or motor development were mediated versus direct effects also varied by cohort (0%–100%). These associations were mediated by child anthropometric status in early infancy (LAZ at 6/9 months: 29%–86% and BMIZ at 9 months: 10%–21%), and to a smaller degree by caregiving practices (6%–20%). Other maternal factors related to language development (age and cognition in DYAD‐G and DYAD‐M, respectively) were mediated to a small extent by caregiving (10%–20%), but were largely direct associations. Other maternal factors related to motor development (CRP in DYAD‐G, health literacy and late gestation cortisol in DYAD‐M) were direct associations.

Associations between infant feeding practices and language or motor development (pathway 3) were direct associations and were not found to be mediated by child nutritional status. Results from the ZINC cohort supported pathway (4.1). Children with poorer nutritional status (linear growth, ponderal growth, Hb) were provided with fewer play materials and interacted less with caregivers, mediating 21%–24% of associations with language scores and 14%–30% of associations with motor scores. In DYAD‐M, children with lower LAZ at birth were also provided with fewer play materials, mediating 8% of the association with motor development.

Results from the DOSE cohort supported pathway (4.2). Children whose linear and ponderal growth status (*z*‐scores), and iron status (ZPP) declined more rapidly from 6 to 18 months were less active at 18 months, mediating 5%–19% of these associations with motor development. We did not find evidence that child cortisol mediated the association between any of the child factors and development (pathway 4.3).

**Figure 5:**
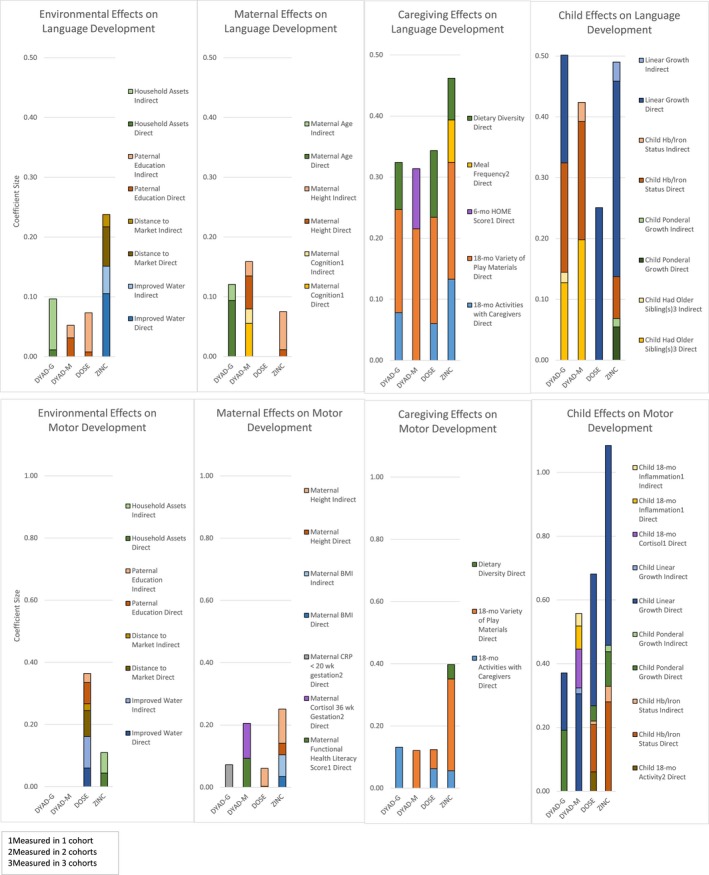
Coefficients of direct and indirect effects on language and motor development in the final models. Note. Coefficient size represents the change in *SD* of the language or motor score with 1 *SD* change in the independent variable, adjusted for all other variables in the model

Overall, caregiving and child factors showed stronger associations with child motor and language development, as compared to environmental and maternal factors, even when considering both indirect and direct effects (Figure 5).

Ratings of the child's behavior during the KDI assessment were the strongest predictor of the child's motor score, with coefficients ranging from .50 to .67 (Table S14; these coefficients are not included in Figure 5). Child behavior also mediated associations of four other factors with motor development: paternal education, unimproved water, linear growth (DOSE), and inflammation at age 18 months (DYAD‐M).

Two child factors and two caregiving factors mediated the effect of the iLiNS‐ZINC intervention on development. Children in the intervention group had greater linear growth from 9 to 18 months, mediating 13% of the effect of the intervention on language and 23% of the effect on motor scores. They also had greater increase in Hb from 9 to 18 months, mediating 16% of the effect of the intervention on motor development. Children in the intervention group had a higher variety of play materials, mediating 12% of the effect of the intervention on language and 16% of the effect on motor scores, as well as higher feeding frequency, mediating 11% of the effect on language scores. For both domains, the direct unmediated effect of the intervention was strong and significant (45%–64%).

After correcting *p*‐values for multiple comparisons, only one coefficient for language and six coefficients for motor scores increased from <.05 to >.05, in all cases remaining <.1. These coefficients are specified in Figures 3 and 4.

## Discussion and Conclusion

We conducted path analyses of 34 environmental, maternal, caregiving, and child factors hypothesized to be associated with 18‐month language and motor development in four large prospective cohorts of children in Ghana, Malawi, and Burkina Faso. Out of 42 indicators of these 34 factors examined, three indicators emerged as consistent predictors of both language and motor scores in three to four cohorts: linear growth throughout infancy and 18‐month variety of play materials and activities with caregivers. For language development, two other consistent predictors in three to four cohorts were dietary diversity and indicators of child Hb/iron status (Hb in DYAD‐G and DYAD‐M; ZPP in ZINC). For motor development, one other consistent predictor in three cohorts was ponderal growth status in early infancy (BMIZ or WLZ at birth/6/9 months). Overall, caregiving and child factors showed stronger and more consistent associations with child motor and language development than environmental and maternal factors, even when considering both indirect and direct effects. While specific pathways differed across cohorts, in general direct effects were stronger and more prevalent than indirect effects.

A strength of the study was the availability of data from four cohorts of children in three African countries, with many variables harmonized across cohorts, enabling the replication of results. Other strengths were the large number of children in each cohort and the large number of factors examined (34 factors, as compared to a maximum of 7 factors in previous studies). Another strength was that the cohorts were enrolled either during pregnancy or at 6 or 9 months postpartum and followed prospectively through 18 months postpartum, allowing these risk factors to be examined throughout most of the first 1,000 days of brain development.

A weakness was that we examined associations and therefore were not able to establish causality. Another limitation was that we were not able to explore certain risk factors or time points in these cohorts due to lack of data, for example, developmental stimulation in early infancy was measured only in one cohort, or because these cohorts did not include children exposed to certain risk factors. Our study is also susceptible to false positive findings due to multiple comparisons, however, we have mitigated this risk by reporting *p*‐values corrected for multiple comparisons and by deriving our conclusions from findings that were replicated across multiple cohorts. In addition, these samples may not be representative of their populations due to the supplements they received. However, we would expect that any bias introduced by the supplementation would tend to decrease rather than increase the magnitude of the associations and therefore our general conclusions regarding consistent significant predictors would not change. For example, in Malawi and Ghana, we found that maternal LNS attenuated the association of linear growth with motor and language scores (Prado, Abbeddou, Adu‐Afarwuah et al., 2016).

Our findings inform the design of interventions to pursue United Nations’ Sustainable Development Goal 4.2 to ‘ensure that all girls and boys have access to quality early childhood development so that they are ready for primary education.’ The consistent results from these four cohorts suggest that key aspects of interventions to promote quality ECD are likely to be promotion of caregiver activities with children, provision of a variety of play materials, and provision of a diverse diet, as well as prevention of faltering in linear and ponderal growth and improvement in Hb/iron status. Since our analysis cannot determine causality, randomized trials should be conducted evaluating large‐scale programs that focus on these key elements. Cost analyses should also be included to identify the most cost‐effective strategies for promoting ECD. Among factors that were only measured in one or two cohorts, factors that were associated with language development were maternal cognition and frequency of child feeding, while factors that were associated with motor development were maternal health literacy, maternal and child basal cortisol, 18‐month inflammation, and 18‐month physical activity, suggesting that these are important factors for further research.

A meta‐analysis of 21 interventions aimed at enhancing developmental stimulation and 18 interventions that provided supplemental nutrition in children under age 2 years in LMICs found that stimulation had a medium effect size of .42 and supplemental nutrition had a small effect size of .09 on child development (Aboud & Yousafzai, 2015). Together with our findings, this highlights the importance of targeting developmental stimulation from birth to age 2 years, even before children start preschool. Many LMICs have begun to invest in programs to promote adequate nutrition during the first 1,000 days, however, few large‐scale programs targeting developmental stimulation currently exist. Of the 21 stimulation interventions reviewed, most had small sample sizes, with only three studies with >350 children. Programs to enhance developmental stimulation need to be developed and evaluated that can be implemented on a large scale. Despite our finding that dietary diversity, child growth, and Hb/iron status were consistently associated with child development in these cohorts, the inconsistent effect of LNS on child development in our studies and the relatively small effect of supplementation across the 18 studies reviewed (Aboud & Yousafzai, 2015) demonstrates a need to develop new nutrition interventions that are more effective to enhance child development. The comparable magnitude of the coefficients for both categories of factors highlights the importance of programs integrating both nutrition and developmental stimulation in early childhood.

The early emergence of socioeconomic disparities in child development in these cohorts also underscores the importance of interventions targeting this early period. Our findings are consistent with several studies in LMICs showing that developmental scores of children in low‐SES households diverged from those of children in high‐SES households even before age 2 years (Fernald, Kariger, Hidrobo, & Gertler, 2012; Hamadani et al., 2014). While the results for motor development were not consistent across our cohorts, socioeconomic disparities in language development were mediated to a greater extent by caregiving practices than by maternal or child factors. This is consistent with three previous studies in LMICs showing that indicators of developmental stimulation in the home mediated a greater percentage of the association of cognitive development with SES than mediated by linear growth (Fernald et al., 2012; Hamadani et al., 2014; Rubio‐Codina, Attanasio, & Grantham‐McGregor, 2016).

Factors that were not associated with language or motor development in these cohorts were indicators of maternal education, low maternal Hb/iron status, maternal illness and inflammation, maternal self‐reported stress and depression, lack of exclusive breastfeeding, and child diarrhea, fever, malaria, and acute respiratory infections. One possible explanation is that early language and motor development may be resilient to these risk factors in these cohorts. Another possibility is that inaccuracy in measurement makes it difficult to detect these associations. For example, although the validity of the maternal depression questionnaires was rigorously evaluated in Malawi (R. C. Stewart, Umar, Tomenson, & Creed, 2013), the Perceived Stress Scale was not validated in the local context and therefore may not have accurately measured maternal perceptions of stress. Additionally, while positive associations have been shown between child development and breastfeeding versus not breastfeeding in previous studies (Dee, Li, Lee, & Grummer‐Strawn, 2007), in our samples breastfeeding was almost universal and therefore we were not able to examine this contrast.

In conclusion, interventions to promote quality ECD in LMICs should begin in early life. Interventions targeting caregiving practices, such as encouraging caregiver activities with children, provision of a variety of play materials and a diverse diet, are likely to have positive effects on early language and motor development and reduce inequalities. Randomized trials of large‐scale programs targeting these aspects of caregiving should be conducted, including evaluating cost‐effectiveness. Prevention of faltering in linear and ponderal growth and improvement in Hb/iron status are also key targets for intervention. Further work is required to design nutrition interventions to enhance their effectiveness to improve child development.

**Key points**

- In four prospective cohorts totaling 4,205 children in Africa, out of 42 indicators examined, six were consistently associated with language and/or motor development at age 18 months in three or four cohorts: child linear and ponderal growth, variety of play materials, activities with caregivers, dietary diversity, and hemoglobin/iron status. These are likely to be key factors for targeted interventions to enhance child development.
- At age 18 months, children from low socioeconomic status (SES) households had fallen behind those from higher‐SES households in language development in all four cohorts and in motor development in two cohorts, highlighting the importance of ECD interventions targeting this early period.
- Associations between SES and language development were largely mediated by caregiving practices rather than maternal or child biomedical conditions.

## Supporting information


**Appendix S1**: Supplemental Methods.
**Table S1.** Factors associated with child development in low‐ and middle‐income countries identified in four previous reviews and those included in our analysis.
**Table S2.** Description of variables.
**Table S3.** Interscorer agreement and test–retest reliability of the developmental assessments in Malawi and Ghana.
**Table S4.** Summary statistics for all variables examined in each cohort.
**Table S5.** Variable selection results for cohort A: DYAD‐Ghana.
**Table S6.** Variable selection results for cohort B: DYAD‐Malawi.
**Table S7.** Variable selection results for cohort C: DOSE‐Malawi.
**Table S8.** Variable selection results for cohort D: ZINC‐Burkina Faso.
**Table S9.** Pathway selection results for cohort A: DYAD‐Ghana.
**Table S10.** Pathway selection results for cohort B: DYAD‐Malawi.
**Table S11.** Pathway selection results for cohort C: DOSE‐Malawi.
**Table S12.** Pathway selection results for cohort D: ZINC‐Burkina Faso.
**Table S13.** Coefficients for direct and indirect effects on language development in the final models.
**Table S14.** Coefficients for direct and indirect effects on motor development in the final models
**Figure S1**. Percent of Socio‐Economic Disparities Mediated by Each Category of Factors.
