# Supplementary material for: Predictors and pathways of language and motor development in four prospective cohorts of young children in Ghana, Malawi, and Burkina Faso
Source: J Child Psychol Psychiatry. 2017 May 23;58(11):1264–75. doi: 10.1111/jcpp.12751 (PMC5697619; doi:10.1111/jcpp.12751)
Supplement: Supplementary file 1 — Appendix S1: Supplemental Methods. Table S1. Factors associated with child development in low‐ and middle‐income countries identified in four previous reviews and those included in our analysis. Table S2. Description of variables. Table S3. Interscorer agreement and test–retest reliability of the developmental assessments in Malawi and Ghana. Table S4. Summary statistics for all variables examined in each cohort. Table S5. Variable selection results for cohort A: DYAD‐Ghana. Table S6. Variable selection results for cohort B: DYAD‐Malawi. Table S7. Variable selection results for cohort C: DOSE‐Malawi. Table S8. Variable selection results for cohort D: ZINC‐Burkina Faso. Table S9. Pathway selection results for cohort A: DYAD‐Ghana. Table S10. Pathway selection results for cohort B: DYAD‐Malawi. Table S11. Pathway selection results for cohort C: DOSE‐Malawi. Table S12. Pathway selection results for cohort D: ZINC‐Burkina Faso. Table S13. Coefficients for direct and indirect effects on language development in the final models. Table S14. Coefficients for direct and indirect effects on motor development in the final models Figure S1. Percent of Socio‐Economic Disparities Mediated by Each Category of Factors. [file JCPP-58-1264-s001.docx]

# **Supporting online information for *Predictors and pathways of language and motor development in four prospective cohorts of young children in Ghana, Malawi, and Burkina Faso*by Prado et al.**

We searched PubMed and PsycINFO for papers published since 1960 using the following search terms: (“child development” OR “motor development” OR “language development” OR “cognitive development”) AND (mediat* OR path OR pathway) AND (“low-income country” OR “low-income countries” OR “middle-income country” OR “middle-income countries” OR “low- and middle-income countries” OR “Africa” OR “Asia”). These searches resulted in 248 publications. Based on review of each abstract, we excluded studies conducted in high-income countries, studies that did not report mediation or path analyses, studies that did not examine child motor, language, or cognitive development scores, and studies that did not include children age 0-18 years, resulting in 18 studies that met the inclusion criteria (Boivin et al., 2016; Fernald, Kariger, Hidrobo, & Gertler, 2012; Frongillo et al., 2016; Hamadani et al., 2014; Jukes & Grigorenko, 2010; Kitsao-Wekulo et al., 2013; Knauer et al., 2016; Lopez Boo, 2016; McCoy, Zuilkowski, & Fink, 2015; Murray et al., 2016; Obradovic, Yousafzai, Finch, & Rasheed, 2016; Prado, Abbeddou, Yakes Jimenez, Some, Dewey, et al., 2016; Rubio-Codina, Attanasio, & Grantham-McGregor, 2016; Santibañez & Fagioli, 2016; Singla, Kumbakumba, & Aboud, 2015; Tran, Luchters, & Fisher, 2016; Tran et al., 2014; Woldehanna, 2016).

Of these 18 studies, nine investigated mediators of socio-economic disparities (SED) in child development. The most common mediators of SED examined were linear growth status in six studies, indicators of home stimulation in five studies, and pre-school or school attendance or exposure to mathematics instruction in four studies. All of these factors were found to be significant mediators of associations between socio-economic status (SES) and at least one cognitive score. In three studies, indicators of developmental stimulation in the home mediated a greater percentage of the association with SES than mediated by linear growth.

Of the 18 studies, six investigated mediators of the effects of interventions on child development. In four parenting interventions, effects on child development were mediated by increases in developmental stimulation in the home and/or the quality of mother-child interactions. In a nutrition intervention, effects on child development were partly mediated by increases in child growth and Hb. In an infant and young child feeding intervention, increases in child development scores were partially mediated through improved minimum dietary diversity and the consumption of iron-rich food.

The other three studies examined mediators of associations between child development and malaria episodes, maternal iron status and depression, and ethnicity. In Uganda, associations between child malaria episodes and child development were partly mediated by anemia episodes. In Vietnam, associations of pre-natal maternal iron status and depression with child development at age 6 months were not mediated by birth weight or preterm birth. Differences in cognitive performance between Wolof and Mandinka children in the Gambia were mediated by participation in primary school and migration to the city.

Regarding pathway (3.1): Effects of infant feeding practices on child development may be mediated by child nutritional status, this association could also be bi-directional, with child factors also influencing feeding practices (Surkan et al., 2015); however, since we could only model one direction in the path analysis, we did not test this hypothesis. Pathway (4.3): Children who are stunted or anemic may have higher basal cortisol, which may affect child development (Felt et al., 2012; Fernald & Grantham-McGregor, 1998).

**Appendix S1: Supplemental Methods**

iLiNS Project Trial Designs

In the iLiNS-ZINC trial in Burkina Faso, the intervention group received 20 g LNSs/d containing different amounts of zinc and weekly diarrhea and malaria surveillance and treatment, while the control group received no contact between enrollment (9 months) and 18 months. In the iLiNS-DOSE trial, the intervention group received different daily doses of LNSs (10-40 g/day) and weekly diarrhea and malaria surveillance and referral, while the control group only received weekly diarrhea and malaria surveillance and referral from 6 to 18 months. In the two iLiNS-DYAD trials, the intervention group received maternal LNSs (20 g/d) during pregnancy and until 6 months postpartum and child LNS (20 g/d) from age 6 to 18 months. The first control group received a daily maternal multiple micronutrient (MMN) tablet until 6 months postpartum and the second control group received a daily iron and folic acid (IFA) tablet until delivery and a placebo tablet from delivery to 6 months postpartum. Children in the two control groups did not receive LNSs. All groups received bi-weekly morbidity surveillance and referral during pregnancy and weekly morbidity surveillance and referral for the infant after birth.

Ethical approval for the iLiNS-ZINC study procedures was obtained from the Institutional Review Board of the University of California Davis and the Comitéd’EthiqueInstitutionnel du Centre Muraz, Bobo Dioulasso. The study was registered with the U.S. National Institute of Health as a clinical trial (www.ClinicalTrials.gov; NCT00944281).Ethical approval for the iLiNS-DOSE and iLiNS-DYAD-M study procedures was obtained from the University of Malawi, College of Medicine Research and Ethics Committee and the Ethics Committee at Tampere University Hospital District, Finland. These studies were also registered with the U.S. National Institute of Health as a clinical trial (www.ClinicalTrials.gov; NCT00945698 and NCT01239693). Ethical approval for theiLiNS-DYAD-G study procedures was obtained from the Ethics Committees at the University of California, Davis, the Ghana Health Service, and the University of Ghana Noguchi Memorial Institute for Medical Research(www.ClinicalTrials.gov; NCT00970866). All participants provided written informed consent, by signature or thumb-print.

Participants

Maternal mortality in the samples assessed for 18-month development was 5/1023 (<1%) children in DYAD-G in Ghana and 2/675 (<1%) children in DYAD-M in Malawi. Maternal mortality was not recorded in DOSE or ZINC.

Baseline characteristics of the developmental samples compared to those who enrolled but were not assessed have been published previously (Prado, Abbeddou, Yakes Jimenez, Some, Ouedraogo, et al., 2016; Prado, Adu-Afarwuah, et al., 2016; Prado, Maleta, et al., 2016; Prado, Phuka, et al., 2016). Cohort A was the only cohort in which no significant differences were found; however, in cohorts C and D, the absolute differences were small: in Cohort C, 0.1 SD difference in maternal education; in Cohort D, differences of 0.09 SD / 2 to 6 percentage points in in baseline WLZ, maternal marital status, and diarrhea prevalence. Thus, they are likely of little practical significance and it is unlikely that the developmental sample is not representative of the full sample. In Cohort B, a large proportion of the mothers of children who did not participate in developmental assessment (70%) had enrolled at the public hospital in Mangochi, which was a more transient area compared to the other two sites at Malindi and Lungwena. Compared to those who did participate in developmental assessment, enrolled mothers whose children did not participate were significantly younger and had higher BMI. A greater proportion were primiparous and a smaller proportion had a household asset index below the median.

*Analysis*

For the DMC-II scores, we calculated z-scores in the iLiNS-ZINC sample. For the KDI and CDI, we calculated z-scores on the full sample of children from the three DOSE and DYAD cohorts. To reduce skewness, the KDI and DMC-II motor scores were log-transformed and the CDI vocabulary score was square root transformed before calculating the z-scores. We examined the distribution of each independent variable separately by trial cohort. We log-transformed skewed variables and truncated outliers to the 1st and 99th percentile. If transformation did not result in a normal distribution, we created a binary variable.

*Step 1: Variable Selection*. First, we examined independent associations between each factor and each developmental score and dropped any that were not associated at *p*<0.05. For any variables that represented the same indicator over time (e.g. maternal Hb at baseline, change in Hb baseline to 36 wk gestation, and change in Hb 36 wk gestation to 6 mo pp), we considered those as a block and entered them together into the same model. If any of a block of variables was significant (e.g., change in Hb baseline to 36 wk gestation only), the others were also retained as covariates. No variables within these sets were highly collinear (*r*s< 0.6). Second, we examined bivariate associations between predictors to check for collinearity. If two variables were highly collinear (*r*>0.6), we dropped the one that was less strongly associated with the developmental score. Third, we examined six multivariate models with each category of factors together predicting developmental score and dropped any that were not associated at *p*<0.05. If, at this step, no variable was significantly associated when controlling for the others, we retained the one that was most strongly associated with the developmental score. The six multivariate models were: (1) all environmental factors together, (2) all maternal factors together, (3) all caregiving factors together, (4) child morbidity and nutritional status (5) child activity based on accelerometer counts (DOSE and DYAD-M only) and (6) rating of the child’s behavior during assessment (motor scores for DOSE, DYAD-M, and DYAD-G only).

*Step 2: Path Selection.* After the final set of variables was determined, we first examined the association between each pair of variables on each of the hypothesized pathways, to determine which variables were potential mediators. If any variables were not associated at p <0.05, we dropped that pathway. All analyses up to this point were conducted using SAS version 9.4 (SAS Institute, Cary, NC). Next, for each independent variable with potential mediators, we tested the multiple mediation model using the Stata version 14.1 (StataCorp, College Station, TX) binary mediation program including all potential mediators together. For each potential mediator for which the indirect effect was significant, we included the mediation pathway in the overall path model.

Finally, we ran the final path model using the sem command in Stata with the mlmv option to estimate the model on the full dataset using maximum likelihood for missing values. All models with language or motor score as the dependent variable included 4 covariates: trial group (intervention or control group), child sex and age at developmental assessment, and developmental data collector. Since the intervention affected developmental scores in the ZINC trial only, we additionally examined the potential mediators of intervention versus control group in this cohort only. For language scores, we also included one additional covariate: whether the child was exposed to more than one language. If, for any variable, the association with the developmental score was not significant at *p*<0.1 in the final model and it was not mediated by another variable, we dropped it and re-ran the final model. If any pathway between an independent variable and a mediator was not significant in the final model at p <0.1 we retained it in the model but did not include it in the path diagrams.

Table S1. Factors associated with child development in low- and middle-income countries identified in four previous reviews and those included in our analysis

| Category | 22 Factors identified in previous reviews and  included here | 12 Factors not included in previous reviews and  included here | 22 Factors identified in previous reviews and  not included here |
| --- | --- | --- | --- |
| Environmental | - Maternal education - Paternal education - Poor sanitation | - Socio-economic status - Poor water quality | - Poor hygiene - Exposure to toxins (lead, arsenic, manganese, pesticides, methyl-mercury) - Exposure to violence - Crowded or chaotic home environments - Refugee status - Pre-school attendance - Social support for parents and/or children |
| Maternal | - Maternal undernutrition - Maternal iron deficiency anemia - Maternal multiple micronutrient supplementation - Maternal infection during pregnancy - Maternal HIV - Maternal depression - Maternal stress | - Maternal age - Maternal inflammation - Maternal cognition | - Maternal omega-3 fatty acid deficiency - Maternal chronic disease, including gestational diabetes - Maternal tobacco use - Maternal alcohol consumption - Maternal ability to cope with stress |
| Caregiving | - Breastfeeding practices - Cognitive stimulation - Caregiver sensitivity and responsivity | - Infant and young child feeding practices | - Institutionalization - Being orphaned |
| Child | - Preterm birth - Intrauterine growth restriction - Linear growth retardation - Iron deficiency anemia - Child multiple micronutrient supplementation^1^ - Diarrhea - Malaria - Child stress - Parity/birth order | - Child ponderal growth - Child inflammation - Child fever - Child acute respiratory infection - Child physical activity - Child behavior during assessment | - Birth complications, including asphyxia - Hypothermia - Neonatal sepsis - Neonatal tetanus - Iodine deficiency - Omega-3 fatty acid deficiency - Zinc deficiency - Intestinal helminth/other parasitic infection |

^1^Child multiple micronutrient supplementation was provided as lipid-based nutrient supplements fortified with 22 vitamins and minerals

TableS2. Description of Variables

|  |  |  | Cohort A:  DYAD-Ghana | | | | | Cohort B:  DYAD-Malawi | | | | Cohort C:  DOSE-Malawi | | | | Cohort D:  ZINC-Burkina Faso | | | |  |  |  |
| --- | --- | --- | --- | --- | --- | --- | --- | --- | --- | --- | --- | --- | --- | --- | --- | --- | --- | --- | --- | --- | --- | --- |
| Environmental Factors | | |  | |  | | |  | |  | |  | |  | |  | |  | |  |  |  |
|  | Poverty | |  | |  | | |  | |  | |  | |  | |  | |  | |  |  |  |
|  |  | Asset Index | For each trial, we used principal components analysis to construct an asset index based on ownership of a set of assets and household characteristics.(Vyas & Kumaranayake, 2006) | | | | | | | | | | | | | | | | |  |  |  |
|  |  | Household Food Insecurity Access Scale | The household food insecurity access (HFIA) scale was based on a set of questions that captures perceptions and reported experiences of three domains of food insecurity: (1) anxiety about the household food supply; (2) insufficient quality; and (3) insufficient food intake and its physical consequences.(Coates, Swindale, & Bilinsky, 2007) In each trial, we calculated the z-score by month of collection, in order to adjust for seasonal changes in food security. | | | | | | | | | | | | | | | | |  |  |  |
|  |  | Distance to Nearest Market | | Same as cohorts C and D | | | | - | | | | Coordinates of household and market locations were collected by handheld Global Positioning Systems (GPS). The shortest route along the road network (in meters) from each household to the nearest market was calculated using ArcMap (Environmental Systems Research Institute, Inc., Redlands, CA). | | | | | | | |  |  |  |
|  | Access to Education | |  | |  | | |  | |  | |  | |  | |  | |  | |  |  |  |
|  |  | Maternal Education | Years of completed formal education | | | | | | | | | | | | | | | | |  |  |  |
|  |  | Paternal Education | Years of completed formal education | | | | | | | | | | | | | | | | |  |  |  |
|  | Water, Sanitation, and Hygiene | |  | |  | | |  | |  | |  | |  | |  | |  | |  |  |  |
|  |  | Unimproved water source | Unimproved water: unprotected well, surface water (river, pond, lake, etc.)  Improved water: indoor or outdoor plumbing or pipe, water vendor, borehole, protected well | | | | | Unimproved water: unprotected well, lake, river  Improved water: piped water, borehole, protected well | | | | | | | | Unimproved water: uncovered well, surface water (river, etc.)  Improved water: piped water inside or outside the household compound, covered well, pump | | | |  |  |  |
|  |  | Unimproved toilet facility | Unimproved toilet: pan/bucket, no toilet facility (bush, beach, etc.)  Improved toilet: public or private flush toilet (water closet), private pit latrine | | | | | Unimproved toilet: regular pit latrine, no toilet facility  Improved toilet: water closet, improved pit latrine | | | | | | | | Unimproved toilet: unimproved latrine, no toilet facility (nature)  Improved toilet: water closet with running water, improved latrine | | | |  |  |  |
| Maternal Factors | | | |  | |  | |  | |  | |  | |  | |  | |  | |  |  |  |
|  | Maternal Age | | | Age in years at baseline | | | | | | | | | | | | | | | |  |  |  |
|  | Maternal Nutritional Status | | |  | |  | |  | |  | |  | |  | |  | |  | |  |  |  |
|  |  | Maternal Height | | Height in cm at baseline | | | | | | | | | | | | | | | |  |  |  |
|  |  | Maternal Body Mass Index (BMI) | | Calculated as kg/m^2^ at baseline | | | | | | | | | | | | | | | |  |  |  |
|  |  | Maternal Baseline^c^Hb (g/dL) | | Determined from venous blood samples using Hemocue (Hemocue AG, Wetzikon, Switzerland) | | | | Determined using on-site cuvette readers (HemoCue AB; Angelholm). | | | | - | | | | - | | | |  |  |  |
|  |  | Change in Hb (Δg/dL) Baseline^c^ to 36 wk gestation | | Calculated by subtracting Hb at Baseline from Hb at 36 wk gestation | | | | | | | | - | | | | - | | | |  |  |  |
|  |  | Change in Hb (Δg/dL) 36 wk gestation to 6 mo pp | | Calculated by subtracting Hb at 36 wk gestation from Hb at 6 mo pp | | | | | | | | - | | | | - | | | |  |  |  |
|  |  | Maternal Baseline^c^ ZPP (μmol/molheme) | | Determined from venous blood samples using Hematofluorometer (Aviv Biomedical Co. NJ, USA), after red blood cells were washed three times with normal saline. | | | | | | | | - | | | | - | | | |  |  |  |
|  |  | Change in ZPP(Δμmol/molheme) Baseline^c^ to 36 wk gestation | | Calculated by subtracting ZPP at Baseline from ZPP at 36 wk gestation | | | | | | | | - | | | | - | | | |  |  |  |
|  |  | Change in ZPP(Δμmol/molheme) 36 wk gestation to 6 mo pp | | - | | | | Calculated by subtracting ZPP at 36 wk gestation from ZPP at 6 mo pp | | | | - | | | | - | | | |  |  |  |
|  |  | Maternal Baseline^c^sTfR (mg/L) | | - | | | | Determined from venous blood samples by immunoturbidimetry on the Cobas Integra 400 system autoanalyzer (F. Hoffmann-La Roche Ltd, Basel, Switzerland). | | | | - | | | | - | | | |  |  |  |
|  |  | Change in sTfR (Δmg/L)Baseline^c^ to 36 wk gestation | | - | | | | Calculated by subtracting sTfR at Baseline from sTfR at 36 wk gestation | | | | - | | | | - | | | |  |  |  |
|  | Maternal Illness and Inflammation | | |  | |  | |  | |  | |  | |  | |  | |  | |  |  |  |
|  |  | Maternal HIV Positive | | - | | | | Determined using a whole-blood antibodyrapid test (Alere Determine HIV-1/2; Alere Medical Co,Ltd.). If the result was positive, the test was repeated by usinganother whole-blood antibody rapid test (Uni-Gold HIV; Trinity  Biotech plc). | | | | - | | | | - | | | |  |  |  |
|  |  | Maternal Malaria at Baseline^c^: Positive RDT | | Determined using Malaria Rapid Diagnostic Test (Clearview Malaria Combo,Vision Biotech, South Africa) | | | | Determined using Malaria Rapid Diagnostic Test (Clearview Malaria Combo; BritishBiocell International Ltd.) | | | | - | | | | - | | | |  |  |  |
|  |  | Maternal Baseline^c^ AGP (g/L) | | Determined using aCobas Integra 400 plus  Automatic Analyzer (Roche Diagnostic Corp., Indianapolis, IN, USA). | | | | Determined using a Cobas Integra 400 system autoanalyzer (F. Hoffmann-La Roche Ltd, Basel, Switzerland) | | | | - | | | | - | | | |  |  |  |
|  |  | Maternal AGP 36 wk gestation | |  |  |  |  |  |  |  |  | - | | | | - | | | |  |  |  |
|  |  | Maternal Elevated AGP | | >=1 g/L at both time points | | | | | | | | - | | | | - | | | |  |  |  |
|  |  | Maternal Baseline^c^ CRP (mg/L) | | Determined using a Cobas Integra 400 plus  Automatic Analyzer (Roche Diagnostic Corp., Indianapolis, IN, USA). | | | | Determined using a Cobas Integra 400 system autoanalyzer (F. Hoffmann-La Roche Ltd, Basel, Switzerland) | | | | - | | | | - | | | |  |  |  |
|  |  | Maternal CRP 36 wk gestation | |  |  |  |  |  |  |  |  | - | | | | - | | | |  |  |  |
|  |  | Maternal Elevated CRP | | >=5 mg/L at both time points | | | | | | | | - | | | | - | | | |  |  |  |
|  | Maternal Stress | | |  | | |  | |  | |  | |  | |  | |  | |  | | |  |
|  |  | Maternal Baseline^c^ Basal Cortisol (nmol/L) | | Saliva was collected after fasting for 30 minutes using an inert polymer swab (Salimetrics Oral Swab). Samples were measured in duplicate using a high sensitivity ELISA test kit (Salimetrics, Carlsbad, CA). We calculated the residual of basal cortisol (nmol/L, log-transformed) predicted by time of day the saliva sample was taken, time since waking, and time since last meal, in order to adjust for these factors. | | | | | | | | | - | | | | - | | | | |  |
|  |  | Maternal Basal Cortisol (nmol/L) at 28 wk gestation | |  |  |  |  |  |  |  |  |  | - | | | | - | | | | |  |
|  |  | Maternal Basal Cortisol (nmol/L) at 36 wk gestation | |  |  |  |  |  |  |  |  |  | - | | | | - | | | | |  |
|  |  | Mean Maternal Basal Cortisol across time points | | Calculated as the mean of all available time points for each participant. | | | | | | | | | - | | | | - | | | | |  |
|  |  | Maternal Baseline Perceived Stress Scale (PSS) score | | - | | | | | Assessed using the Perceived Stress Scale(Cohen, Kamarck, & Mermelstein, 1983; C. P. Stewart et al., 2015) | | | | - | | | | - | | | | |  |
|  |  | Maternal PSS score at 28 wk gestation | | - | | | | |  |  |  |  | - | | | | - | | | | |  |
|  |  | Maternal PSS score at 36 wk gestation | | - | | | | |  |  |  |  | - | | | | - | | | | |  |
|  |  | Mean Maternal PSS score across time points | | - | | | | | Calculated as the mean of all available time points for each participant | | | | - | | | | - | | | | |  |
|  | Maternal Depression 6 mo pp | | |  | | |  | |  | |  | |  | |  | |  | |  | | |  |
|  |  | Maternal Depression Score | | Assessed using the Edinburgh Post-natal Depression Scale (EPDS).(Cox, Holden, & Sagovsky, 1987) | | | | | Assessed using the Self-Reporting Questionnaire (SRQ)(Robert C. Stewart et al., 2016; R. C. Stewart et al., 2009; WHO, 1994) | | | | - | | | | - | | | | |  |
|  | Maternal Cognition 6 mo pp | | |  | | |  | |  | |  | |  | |  | |  | |  | | |  |
|  |  | Maternal Cognitive Z-Score | | - | | | | | Calculated as the mean z-score across five cognitive tests z-scores: digit span forward and backward, verbal fluency for food and people’s names, and mental rotation test. | | | | - | | | | - | | | | |  |
|  |  | Maternal Functional Health Literacy Score | | - | | | | | This test measures the mother’s understanding of health messages written in words and pictures, such as medication instructions, health cards, and growth charts. The score is the number of questions answered correctly out of a maximum 36 points. | | | | - | | | | - | | | | |  |
| Child Factors | | | |  | | |  | |  | |  | |  | |  | |  | |  | | |  |
|  |  | Firstborn | | Children of primiparous mothers were considered firstborn. | | | | | | | | | - | | | | Children were considered firstborn if there were no older siblings in the household roster. | | | | |  |
|  |  | Born Preterm (< 37 wk) | | Gestational age at enrolment was mainly determined by ultrasound. | | | | | | | | | - | | | | - | | | | |  |
|  | Child Linear Growth | | |  | | |  | |  | |  | |  | |  | |  | |  | | |  |
|  |  | LAZ at Birth/6/9 mo | | LAZ was measured within 14 days of birth, with 91% measured within 48 hours of birth, and was calculated based on WHO norms(WHO Multicentre Growth Reference Study Group, 2006) | | | | | LAZ was measured within 6 weeks of birth, with a mean (SD) of 13 (6) days after birth, and was calculated based on WHO norms(WHO Multicentre Growth Reference Study Group, 2006) | | | | LAZ at 6 months was calculated based on WHO norms(WHO Multicentre Growth Reference Study Group, 2006) | | | | LAZ at 9 months was calculated based on WHO norms(WHO Multicentre Growth Reference Study Group, 2006) | | | | |  |
|  |  | Postnatal Linear Growth: Change in LAZ Birth/6/9 to 18 mo | | Calculated by subtracting LAZ at birth from LAZ at 18 months | | | | | | | | | Calculated by subtracting LAZ at 6 months from LAZ at 18 months | | | | Calculated by subtracting LAZ at 9 months from LAZ at 18 months | | | | |  |
|  | Child Ponderal Growth | | |  | | |  | |  | |  | |  | |  | |  | |  | | |  |
|  |  | BMIZ at Birth/WLZ at 6/9 mo | | Length and weight were measured within 6 weeks of birth and BMIZ was calculated based on WHO norms(WHO Multicentre Growth Reference Study Group, 2006) | | | | | | | | | BMIZ at 6 months was calculated based on WHO norms(WHO Multicentre Growth Reference Study Group, 2006) | | | | BMIZ at 9 months was calculated based on WHO norms(WHO Multicentre Growth Reference Study Group, 2006) | | | | |  |
|  |  | Postnatal Ponderal Growth: Change in BMIZ/WLZ from Birth/6/9 mo to 18 mo | | Calculated by subtracting BMIZ at birth from BMIZ at 18 months | | | | | | | | | Calculated by subtracting BMIZ at 6 months from BMIZ at 18 months | | | | Calculated by subtracting BMIZ at 9 months from BMIZ at 18 months | | | | |  |
|  | Child Hb/Iron Status | | |  | | |  | |  | |  | |  | |  | |  | |  | | |  |
|  |  | Child 6 or 9-moHb (g/dL) | | Hb at 6 months was determined using on-site cuvette readers (HemoCue AB; Angelholm). | | | | | | | | | | | | | Hb at 9 months was determined using on-site cuvette readers (HemoCue AB; Angelholm). | | | | |  |
|  |  | Change in Hb 6 or 9 to 18 mo | | Calculated by subtracting Hb at 6 months from Hb at 18 months | | | | | | | | |  | | - | | Calculated by subtracting Hb at 9 months from Hb at 18 months | | | | |  |
|  |  | Child 6 or 9-mo ZPP(Δμmol/molheme) | | ZPP at 6 months determined using a hematofluorometer (206D, AVIV Biomedical Inc., Lakewood, NJ, USA). | | | | | | | | | | | | | ZPP at 9 months was determined using a hematofluorometer (206D, AVIV Biomedical Inc., Lakewood, NJ, USA). | | | | |  |
|  |  | Change in ZPP 6 or 9 to 18 mo | | Calculated by subtracting ZPP at 6 months from ZPP at 18 months | | | | | | | | | | | | | - | | | | |  |
|  | Child Illness and Inflammation | | |  | | |  | |  | |  | |  | |  | |  | |  | | |  |
|  |  | Child 6-mo AGP (g/L) | | - | | | | | Determined using a Cobas Integra 400 system autoanalyzer (F. Hoffmann-La Roche Ltd, Basel, Switzerland) | | | | - | | | | - | | | | |  |
|  |  | Child 18-mo AGP (g/L) | | - | | | | |  |  |  |  | - | | | | - | | | | |  |
|  |  | Child Elevated AGP | | - | | | | | >=1 g/L at both time points | | | | - | | | | - | | | | |  |
|  |  | Child 6-mo CRP (mg/L) | | - | | | | | Determined using a Cobas Integra 400 system autoanalyzer (F. Hoffmann-La Roche Ltd, Basel, Switzerland) | | | | - | | | | - | | | | |  |
|  |  | Child 18-mo CRP (mg/L) | | - | | | | |  |  |  |  | - | | | | - | | | | |  |
|  |  | Child Elevated CRP | | - | | | | | >=5 mg/L at both time points | | | | - | | | | - | | | | |  |
|  |  | Highest Quartile Diarrhea Prevalence (>=3.5% of days) | | Diarrhea was defined as caregiver report of three or more loose or liquid stools per 24 hours. Diarrhea prevalence was calculated as the proportion of days on which the child experienced diarrhea among all days of observation for that child, excluding children with < 90 days of observation. | | | | | | | | | | | | | Defined and calculated in the same way as the other cohorts, excluding children with < 30 days of observation. | | | | |  |
|  |  | Diarrhea Incidence (episodes per year) | | - | | | | | - | | | | An episode of diarrhea was defined as the period starting the day the child first had diarrhea following a diarrhea-free period of 2 days, and ending on the last day the child had diarrhea that was followed by ≥2 days without diarrhea. | | | | | | | | |  |
|  |  | Highest Quartile Fever Prevalence (>=2.5% of days) | | Fever was defined as any reported fever by the caregiver in the absence of respiratory symptoms (cough, rapid breathing, difficult breathing or nasal discharge) or diarrhea. Fever prevalence was calculated as the proportion of days on which a child experienced fever among all days of observations for that child. | | | | | Fever was defined as any elevated measured auricular temperature (>37.5°C). Fever prevalence was calculated as the proportion of days on which the child experienced fever among all days of observation for that child, excluding children with < 90 days of observation.  - | | | | | | | | Fever was defined as any reported fever by the caregiver or any elevated measured auricular temperature (>37.5°C). Fever prevalence was calculated as the proportion of days on which the child experienced fever among all days of observation for that child, excluding children with < 30 days of observation. | | | | |  |
|  |  | Malaria or Undefined Fever Incidence (episodes per year) | | - | | | | | - | | | | Fever episodes in the absence of diarrhea and respiratory symptoms, with or without other symptoms, were categorized as ‘undefined fever’. Although we did not routinely test for malaria during home visits, cases of ‘undefined fever’ could be classified as suspected malaria cases according to the Integrated Management of Childhood  Illnesses (IMCI) classification for high malaria risk areas. | | | | Any reported or confirmed fever during the 24 h preceding the morbidity visit, associated with a positive RDT was defined as malaria.A child was considered at risk for malaria if the child did not receive any antimalarial treatment within the previous 21 days. Malaria incidence was defined as the number of new episodes of malaria per 100 child-days at risk. This was converted to episodes per 365 child days for Table 1, for comparability with Malawi, but episodes per 100 days was used in the analysis. | | | | |  |
|  |  | ARI Prevalence (% of days) | | If diarrhea was absent but the caregiver reported any respiratory symptoms (cough, nasal obstruction, wheezing, severe difficulty breathing, rapid breathing, or nasal discharge), a diagnosis of ARI was made. | | | | | - | | | | If diarrhea was absent but the caregiver reported any respiratory symptoms (cough, rapid or difficult breathing and nasal discharge) with or without fever, a diagnosis of ARI was made. | | | | Acute respiratory illness (ARI) was defined as any episode in which the caregiver reported cough with respiratory difficulties (wheezing/stridor or chest  in-drawing) or with a purulent nasal discharge. | | | | |  |
|  | Child Stress | | |  | | |  | |  | |  | |  | |  | |  | |  | | |  |
|  |  | Child 6-mo Basal Cortisol (nmol/L) | | - | | | | | Saliva samples were collected upon arrival at the clinic. Samples were analyzed using ELISA kit (Salimetrics, Carlsbad, CA).We calculated the residual of basal cortisol (nmol/L, log-transformed) predicted by time of day the saliva sample was taken, time since waking, and time since last meal, in order to adjust for these factors. | | | | - | | | | - | | | | |  |
|  |  | Child 12-mo Basal Cortisol (nmol/L) | | - | | | | |  |  |  |  | - | | | | - | | | | |  |
|  |  | Child 18-mo Basal Cortisol (nmol/L) | | - | | | | |  |  |  |  | - | | | | - | | | | |  |
|  |  | Mean Child Basal Cortisol across time points | | - | | | | | Calculated as the mean of all available time points for each participant. | | | | - | | | | - | | | | |  |
|  | Child 18-moPhysical Activity | | |  | | |  | |  | |  | |  | |  | |  | |  | | |  |
|  |  | Accelerometer Mean Vector Magnitude | | - | | | | | Physical activity was measured over 1 week with hip-wornActiGraph GT3X+accelerometer (Pensacola, FL, USA). It recorded accelerationsin three different axes: vertical, antero-posterior and medio-lateral. Wecombined the data from these axes as vector magnitude (VM) counts/15s,which we calculated by taking a square root of the sum of squared activitycounts of each axis. We averaged the daily means of VM counts after excluding night time and strings of ≥20 min of zeroes. We only included participants with minimum of 4 days of ≥6 hours of data.(Pulakka et al., 2013) | | | | | | | | - | | | | |  |
|  |  | Mean % of time spent in moderate to vigorous physical activity | | - | | | | | We calculated the mean daily proportion of time spend in moderate-to-vigorous activity by vertical axis according to the cut-point>=419 counts/15s.(Trost, Fees, Haar, Murray, & Crowe, 2012) | | | | | | | | - | | | | |  |
|  |  | Positive behavior during assessment | | The child’s mood during the KDI assessment was rated as positive (smiling/laughing or occasional smiles) or not positive (crying/inconsolable, occasional crying, changeable/mood swings, or no visible emotions). The child’s interaction with the assessor during the KDI was rated as positive (friendly) or not positive (avoidant and withdrawn, clings to family member, hesitant/when approached will accept reluctantly, difficult to engage in tasks, or inappropriate approaches to assessor). The child’s activity level during the KDI was rated as positive (active and maintains interest) or not positive (unarousable, sleepy, can hardly be awakened, sleepy but easily awake, does not spontaneously engage in activity, and awake but loses interest). The child’s overall behavior during the assessment was coded as positive if all three of these dimensions were rated positive. | | | | | | | | | | | | | - | | | | |  |
| Caregiving Factors | | | |  | | |  | |  | |  | |  | |  | |  | |  | | |  |
|  | Infant Feeding | | |  | | |  | |  | |  | |  | |  | |  | |  | | |  |
|  |  | Exclusive Breastfeeding First 6 mo | | Caregiver reported feeding the child no foods or liquids other than breastmilk in monthly 24-hr dietary recalls from age 1 to 5 months. | | | | | | | | | - | | | | - | | | | |  |
|  |  | 9-mo24-hr feeding frequency | | - | | | | | Caregiver recalls of the number of times child was fed solid or semi-solid food yesterday.(World Health Organization, 2010) | | | | - | | | | Same as Cohort B | | | | |  |
|  |  | 12-mo 24-hr feeding frequency | | - | | | | |  |  |  |  | - | | | | - | | | | |  |
|  |  | 15-mo 24-hr feeding frequency | | - | | | | |  |  |  |  | - | | | | - | | | | |  |
|  |  | 18-mo 24-hr feeding frequency | | - | | | | |  |  |  |  | - | | | | Same as Cohort B | | | | |  |
|  |  | Mean feeding frequency across time points | | - | | | | | Calculated as the mean of all available time points for each participant. | | | | - | | | | Calculated as the mean of all available time points for each participant. | | | | |  |
|  |  | 9-mo24-hr dietary diversity | | - | | | | | - | | | | - | | | | Calculated as the sum of 7 food groups consumed yesterday, with food groups defined as in WHO (2010). World Health Organization, 2010 #6797} | | | | |  |
|  |  | 18-mo24-hr dietary diversity | | - | | | | | - | | | | - | | | |  |  |  |  |  |  |
|  |  | Mean 24-hr dietary diversity | | - | | | | | - | | | | - | | | | Calculated as the mean of all available time points for each participant. | | | | |  |
|  |  | 9-mo 7-day dietary diversity score | | Sum of whether 10 nutrient-rich food groups were fed to child at least 3 days in past week | | | | | | | | | | | | | Sum of whether 10 nutrient-rich food groups plus 1 category for starchy foods were fed to child at least 3 days in past week. | | | | |  |
|  |  | 12-mo 7-day dietary diversity score | |  |  |  |  |  |  |  |  |  |  |  |  |  |  |  |  |  |  |  |
|  |  | 15-mo 7-day dietary diversity score | |  |  |  |  |  |  |  |  |  |  |  |  |  |  |  |  |  |  |  |
|  |  | 18-mo 7-day dietary diversity score | |  |  |  |  |  |  |  |  |  |  |  |  |  |  |  |  |  |  |  |
|  |  | Mean 7-day dietary diversity score | | Calculated as the mean of all available time points for each participant. | | | | | | | | | | | | | | | | | |  |
|  | Nurturing and Stimulation | | |  | | |  | |  | |  | |  | |  | |  | |  | | |  |
|  |  | 6-mo HOME Total Score | | - | | | | | At a home visit 6 mo post-partum, data collectors administered the Infant/Toddler Home Observation for the Measurement of the Environment (HOME) Inventory.(Caldwell & Bradley, 2003) | | | | - | | | | - | | | | |  |
|  |  | 6-mo HOME Responsivity Score | | - | | | | |  |  |  |  | - | | | | - | | | | |  |
|  |  | 6-mo HOME Acceptance Score | | - | | | | |  |  |  |  | - | | | | - | | | | |  |
|  |  | 6-mo HOME Organization Score | | - | | | | |  |  |  |  | - | | | | - | | | | |  |
|  |  | 6-month HOME Learning Materials Score | | - | | | | |  |  |  |  | - | | | | - | | | | |  |
|  |  | 6-mo HOME Involvement Score | | - | | | | |  |  |  |  | - | | | | - | | | | |  |
|  |  | 6-mo HOME Variety Score | | - | | | | |  |  |  |  | - | | | | - | | | | |  |
|  |  | 18-moFCI Variety of Play Materials | | Using the Family Care Indicators (FCI) interview,(Hamadani et al., 2010; Kariger et al., 2012) the mother reportedwhether seven types of play materials were available to the child in the home (e.g. toys for pretending, toys for stacking or building). | | | | | | | | | | | | | | | | | |  |
|  |  | 18-moFCI Activities with Caregivers | | For each of six activities, mothers reported whether the child’s mother, father, and any other adult had engaged in that activity with the child in the past three days (e.g. told stories, sang songs). We calculated the total score as the sum of these 18 item scores (6 activities for each of the three categories of potential caregivers). | | | | | | | | | | | | | | | | | |  |

Table S3*.* Inter-Scorer Agreement and Test-Retest Reliability of the Developmental Assessments in Malawi and Ghana

|  |  | Kilifi Developmental Inventory | Vocabulary Checklist |
| --- | --- | --- | --- |
| Inter-Scorer Agreement^1^ | |  |  |
|  | DOSE | 95% | 96% |
|  | DYAD-M | 92% | 98% |
|  | DYAD-G | 91% | 96% |
| Test-Retest Reliability^2^ | |  |  |
|  | DOSE round 1 | 0.57 | 0.94 |
|  | DOSE round 2 | 0.77 | 0.97 |
|  | DOSE round 3 | 0.87 | 0.81 |
|  | DYAD-M | 0.65 | 0.82 |
|  | DYAD-G round 1 | 0.53 | 0.84 |
|  | DYAD-G round 2 | 0.65 | 0.94 |

^1^Inter-scorer agreement was calculated as the agreement between pairs of data collectors independently scoring the same test session or interview.

^2^Test-retest reliability was calculated as the Pearson’s correlation between the scores of a set of children tested on two different days, separated by a period of ~7 days. After each round of reliability testing, we conducted re-training on items that showed inconsistency between testers or between test sessions in order to improve reliability over time.

TableS4. Summary Statistics for All Variables Examined in Each Cohort

|  |  |  | **Cohort A**:  DYAD-Ghana | | **Cohort B**:  DYAD-Malawi | | **Cohort C**:  DOSE-Malawi | | **Cohort D**:  ZINC-Burkina Faso | |  | Cohorts (A, B, C, D) in which the variable was retained and direction  (+,-) of association | |
| --- | --- | --- | --- | --- | --- | --- | --- | --- | --- | --- | --- | --- | --- |
|  |  |  | n | Mean (SD) or Median (SD) or n (%) | n | Mean (SD) or Median (SD) or n (%) | n | Mean (SD) or Median (SD) or n (%) | n | Mean (SD) or Median (SD) or n (%) |  | Language | Motor |
| Environmental Factors | | |  |  |  |  |  |  |  |  |  |  |  |
|  | Poverty | |  |  |  |  |  |  |  |  |  |  |  |
|  |  | Household Asset Index: mean (SD) | 1021 | 0.03 (0.97) | 667 | -0.33 (0.91) | 1184 | -0.39 ^a^ (0.94) | 1121 | -0.10 ^a^ (1.00) |  | A+ | D+ |
|  |  | Household Food Insecurity Access Scale: median (SD) | 1019 | 0 (4) | 667 | 4 (4) | 1155 | 6 (6) | 1121 | 1 (4) |  | 0 | 0 |
|  |  | Distance to Nearest Market (m): median (SD) | 1016 | 1242 (1860) | - | - | 1385 | 2388 (2739) | 1095 | 801 (1723) |  | D+^b^ | C- |
|  | Access to Education | |  |  |  |  |  |  |  |  |  |  |  |
|  |  | Paternal Education (y): mean (SD) | 869 | 9 (3) | 650 | 5 (4) | 1336 | 6 (4) | - | - |  | B+,C+ | C+ |
|  |  | Father no education: n (%) | - | - | - | - | - | - | 1121 | 340 (30 %) |  | 0 | 0 |
|  |  | Maternal Education (y): mean (SD) | 1023 | 8 (4) | 671 | 4 (3) | 1358 | 5 (4) | - | - |  | 0 | 0 |
|  |  | Mother no education: n (%) | - | - | - | - | - | - | 1121 | 666 (59 %) |  | 0 | 0 |
|  | Water and Sanitation | |  |  |  |  |  |  |  |  |  |  |  |
|  |  | Unimproved water source: n (%) | 1021 | 67 (7 %) | 674 | 60 (9 %) | 1200 | 92 (8 %) | 1121 | 819 (73 %) |  | D- | C- |
|  |  | Unimproved toilet facility: n (%) | 1021 | 26 (3 %) | 674 | 610 (91 %) | 1200 | 1176 (98 %) | 1121 | 1094 (98 %) |  | 0 | 0 |
| Maternal Factors | | |  |  |  |  |  |  |  |  |  |  |  |
|  |  | Maternal Age (y): mean (SD) | 1023 | 27 (5) | 675 | 25 (6) | 1358 | 26 (6) | 1115 | 27 (7) |  | A+ | 0 |
|  | Maternal Nutritional Status | |  |  |  |  |  |  |  |  |  |  |  |
|  |  | Maternal Height (cm): mean (SD) | 1006 | 159 (6) | 672 | 156 (6) | 1383 | 156 (6) | 1119 | 162 (6) |  | B+,D+ | C+,D+ |
|  |  | Maternal Body Mass Index (kg/m^2^): mean (SD) | 1006 | 25 (5) | 671 | 22 (3) | 1379 | 22 (3) | 1119 | 21 (3) |  | 0 | D+ |
|  |  | Maternal Baseline^c^Hb (g/dL): mean (SD) | 1023 | 11 (1) | 674 | 11 (2) | - | - | - | - |  | 0 | 0 |
|  |  | Change in Hb (Δg/dL) Baseline^c^ to 36 wk gestation: mean (SD) | 878 | 0.5 (1.2) | 548 | 0.0 (1.6) | - | - | - | - |  | 0 | 0 |
|  |  | Change in Hb (Δg/dL) 36 wk gestation to 6 mo pp: mean (SD) | 826 | 1.5 (1.1) | 501 | 1.7 (1.9) | - | - | - | - |  | 0 | 0 |
|  |  | Maternal Baseline^c^ ZPP (μmol/molheme): median (SD) | 1023 | 37 (31) | 674 | 41 (40) | - | - | - | - |  | 0 | 0 |
|  |  | Change in ZPP(Δμmol/molheme) Baseline^c^ to 36 wk gestation: mean (SD) | 876 | 2 (28) | 550 | 43 (183) | - | - | - | - |  | 0 | 0 |
|  |  | Change in ZPP(Δμmol/molheme) 36 wk gestation to 6 mo pp: mean (SD) | - | - | 531 | -15 (244) | - | - | - | - |  | 0 | 0 |
|  |  | Maternal Baseline^c^TfR (mg/L): median (SD) | - | - | 656 | 4 (3) | - | - | - | - |  | 0 | 0 |
|  |  | Change in TfR (Δmg/L)Baseline^c^ to 36 wk gestation: mean (SD) | - | - | 556 | 1 (2) | - | - | - | - |  | 0 | 0 |
|  | Maternal Illness and Inflammation | |  |  |  |  |  |  |  |  |  |  |  |
|  |  | Maternal HIV Positive: n (%) | - | - | 672 | 80 (12 %) | - | - | - | - |  | 0 | 0 |
|  |  | Maternal Malaria at Baseline^c^: Positive RDT: n (%) | 1023 | 91 (9 %) | 672 | 152 (23 %) | - | - | - | - |  | 0 | 0 |
|  |  | Maternal Baseline^c^ AGP (g/L): mean (SD) | 1004 | 0.6 (0.2) | 656 | 0.7^a^ (0.2) | - | - | - | - |  | 0 | 0 |
|  |  | Maternal AGP 36 wk gestation: median (SD) | 875 | 0.4 (0.2) | 561 | 0.5 (0.2) | - | - | - | - |  | 0 | 0 |
|  |  | Maternal Elevated AGP (>=1 g/L) at both time points: n (%) | 860 | 4 (0 %) | 545 | 11 (2 %) | - | - | - | - |  | 0 | 0 |
|  |  | Maternal Baseline^c^ CRP (mg/L): median (SD) | 1004 | 3.6 (11.1) | 656 | 3.7 (17.2) | - | - | - | - |  | 0 | A+^b^ |
|  |  | Maternal CRP 36 wk gestation: median (SD) | 875 | 2.1 (16.2) | 561 | 2.7 (15.8) | - | - | - | - |  | 0 | 0 |
|  |  | Maternal Elevated CRP (>=5 mg/L) at both time points: n (%) | 860 | 129 (15 %) | 472 | 84 (15 %) | - | - | - | - |  | 0 | 0 |
|  | Maternal Stress | |  |  |  |  |  |  |  |  |  |  |  |
|  |  | Maternal Baseline^c^ Basal Cortisol (nmol/L): median (SD) | 722 | 4.3 (2.7) | 656 | 4.9 (3.8) | - | - | - | - |  | 0 | 0 |
|  |  | Maternal Basal Cortisol (nmol/L) at 28 wk gestation: median (SD) | 690 | 5.8 (3.3) | 479 | 5.2 (3.7) | - | - | - | - |  | 0 | 0 |
|  |  | Maternal Basal Cortisol (nmol/L) at 36 wk gestation: median (SD) | 602 | 7.5 (3.0) | 556 | 7.6 (2.7) | - | - | - | - |  | 0 | B- |
|  |  | Mean Maternal Basal Cortisol across time points: median (SD) | 878 | 6.2 (2.2) | 672 | 6.0 (2.6) | - | - | - | - |  | 0 | 0 |
|  |  | Maternal Baseline Perceived Stress Scale (PSS) score: mean (SD) | - | - | 631 | 14^d^ (5) | - | - | - | - |  | 0 | 0 |
|  |  | Maternal PSS score at 28 wk gestation: mean (SD) | - | - | 503 | 15^d^ (6) | - | - | - | - |  | 0 | 0 |
|  |  | Maternal PSS score at 36 wk gestation: mean (SD) | - | - | 557 | 14^d^ (6) | - | - | - | - |  | 0 | 0 |
|  |  | Mean Maternal PSS score across time points: mean (SD) | - | - | 673 | 14^d^ (4) | - | - | - | - |  | 0 | 0 |
|  | Maternal Depression 6 mo pp | |  |  |  |  |  |  |  |  |  |  |  |
|  |  | Maternal Depression Score: mean (SD) | 988 | 5.3^e^ (4.8) | 615 | 1.7^f^ (2.6) | - | - | - | - |  | 0 | 0 |
|  | Maternal Cognition 6 mo pp | |  |  |  |  |  |  |  |  |  |  |  |
|  |  | Maternal Cognitive Z-Score: mean (SD) | - | - | 641 | 0^g^ (1) | - | - | - | - |  | B+ | 0 |
|  |  | Maternal Functional Health Literacy Score: mean (SD) | - | - | 643 | 16^h^ (4) | - | - | - | - |  | 0 | B+ |
| Child Factors | | |  |  |  |  |  |  |  |  |  |  |  |
|  |  | Firstborn: n (%) | 1023 | 326 (32 %) | 535 | 138 (21 %) | - | - | 1121 | 203 (18 %) |  | A-,B- | 0 |
|  |  | Born Preterm (< 37 wk): n (%) | 1022 | 77 (8 %) | 626 | 49 (7 %) | - | - | - | - |  | 0 | 0 |
|  | Child Linear Growth | |  |  |  |  |  |  |  |  |  |  |  |
|  |  | LAZ at Birth/6/9 mo: mean (SD) | 985 | -0.61^i^ (0.98) | 619 | -1.1^i^ (1.1) | 1385 | -1.41^j^ (1.04) | 1119 | -1.2^k^ (1.1) |  | A+,C+,D+ | A+,B+,C+,D+ |
|  |  | Postnatal Linear Growth: Change in LAZ Birth/6/9 to 18 mo: mean (SD) | 954 | -0.22^i^ (0.98) | 579 | -0.5^i^ (1.1) | 1360 | -0.45^j^ (0.76) | 1118 | -0.36^k^ (0.61) |  | A+,C+,D+ | A+,B+,C+,D+ |
|  | Child Ponderal Growth | |  |  |  |  |  |  |  |  |  |  |  |
|  |  | BMIZ at Birth/WLZ at 6/9 mo: mean (SD) | 985 | -0.6^i^ (0.99) | 618 | 0.0^i^ (1.07) | 1385 | 0.27^j^ (1.11) | 1120 | -1.04^k^ (1.06) |  | D+ | A+,C+,D+ |
|  |  | Postnatal Ponderal Growth: Change in BMIZ/WLZ from Birth/6/9 mo to 18 mo: mean (SD) | 954 | 0.16^i^ (1.25) | 578 | 0.10^i^ (1.30) | 1360 | -0.55^j^ (0.98) | 1119 | 0.25^k^ (0.84) |  | 0 | A+,C+ |
|  | Child Hb/Iron Status | |  |  |  |  |  |  |  |  |  |  |  |
|  |  | Child 6 or 9-moHb (g/dL): mean (SD) | 856 | 11^j^ (1) | 644 | 10^j^ (2) | 1374 | 10^j^ (2) | 1122 | 8.9^k^ (1.5) |  | A+,B+ | D+ |
|  |  | Change in Hb 6 or 9 to 18 mo: mean (SD) | 856 | 0.0^j^ (1.0) | 628 | 0.5^j^ (1.8) | - | - | 1119 | 0.6^k^ (2) |  | A+,B+ | D+ |
|  |  | Child 6 or 9-mo ZPP(Δμmol/molheme): median (SD) | 808 | 58^j^ (47) | 645 | 204^j^ (296) | 1320 | 83^j^ (70) | 1121 | 168^k^ (116) |  | D- | C-,D- |
|  |  | Change in ZPP 6 or 9 to 18 mo: mean (SD) | 825 | -1^j^ (48) | 619 | -68^j^ (344) | 1031 | -9^j^ (76) | - | - |  | 0 | C- |
|  | Child Illness and Inflammation | |  |  |  |  |  |  |  |  |  |  |  |
|  |  | Child 6-mo AGP (g/L): median (SD) | - | - | 530 | 1.2 (0.4) | - |  |  | - |  | 0 | 0 |
|  |  | Child 18-mo AGP (g/L): median (SD) | - | - | 611 | 1.3 (0.6) | - | - | - | - |  | 0 | B- |
|  |  | Child Elevated AGP (>=1 g/L) at both 6 and 18 mo: n (%) | - | - | 489 | 235 (48 %) | - | - | - | - |  | 0 | 0 |
|  |  | Child 6-mo CRP (mg/L): median (SD) | - | - | 531 | 1.9 (17.9) | - | - | - | - |  | 0 | 0 |
|  |  | Child 18-mo CRP (mg/L): median (SD) | - | - | 612 | 1.9 (27.1) | - | - | - | - |  | 0 | 0 |
|  |  | Child Elevated CRP (>=5 mg/L) at both 6 and 18 mo: n (%) | - | - | 490 | 50 (10 %) | - | - | - | - |  | 0 | 0 |
|  |  | Highest Quartile Diarrhea Prevalence (>=3.5% of days): n (%) | 1023 | 268 (26 %) | 657 | 174 (26 %) | 1364 | 2^a^ (4) | 746 | 2^a^ (3) |  | 0 | 0 |
|  |  | Diarrhea Incidence (episodes per year): median (SD) | - | - | - | - | 1364 | 1 (2) | 746 | 3 (4) |  | 0 | 0 |
|  |  | Highest Quartile Fever Prevalence (>=2.5% of days): n (%) | 1023 | 239 (23 %) | 655 | 136 (21 %) | 1364 | 7^a^ (7) | 746 | 3^a^ (3) |  | 0 | 0 |
|  |  | Malaria or Undefined Fever Incidence (episodes per year): median (SD) | - | - | - | - | 1364 | 1^l^ (2) | 746 | 2^m^ (2) |  | 0 | 0 |
|  |  | ARI Prevalence (% of days): median (SD) | 1023 | 16 (12) | - | - | 1364 | 9 (5) | 746 | 476^n^ (64 %) |  | 0 | 0 |
|  | Child Stress | |  |  |  |  |  |  |  |  |  |  |  |
|  |  | Child 6-mo Basal Cortisol (nmol/L): median (SD) | - | - | 612 | 4.1 (4.9) | - | - | - | - |  | 0 | 0 |
|  |  | Child 12-mo Basal Cortisol (nmol/L): median (SD) | - | - | 601 | 3.5 (5.7) | - | - | - | - |  | 0 | 0 |
|  |  | Child 18-mo Basal Cortisol (nmol/L): median (SD) | - | - | 651 | 3.4 (5.0) | - | - | - | - |  | 0 | B- |
|  |  | Mean Child Basal Cortisol across time points: median (SD) | - | - | 673 | 4.0 (3.3) | - | - | - | - |  | 0 | 0 |
|  | Child 18-mo Activity | |  |  |  |  |  |  |  |  |  |  |  |
|  |  | Accelerometer Mean Vector Magnitude: mean (SD) | - | - | 582 | 301 (56) | 1090 | 304 (65) | - | - |  | 0 | C+ |
|  |  | Mean % of time spent in moderate to vigorous physical activity: mean (SD) | - | - | 582 | 11 (4) | 1090 | 12 (4) | - | - |  | 0 | 0 |
|  |  | Positive behavior during assessment: n (%) | 918 | 545 (59 %) | 668 | 370 (55 %) | 1332 | 354 (27 %) | - | - |  | - | A+,B+,C+ |
| Caregiving Factors | | |  |  |  |  |  |  |  |  |  |  |  |
|  | Infant Feeding | |  |  |  |  |  |  |  |  |  |  |  |
|  |  | Exclusive Breastfeeding First 6 mo: n (%) | 794 | 423 (53 %) | 248 | 25 (10 %) | - | - | - | - |  | 0 | 0 |
|  |  | 9-mo24-hr feeding frequency: mean (SD) | - | - | 542 | 2 (1) | - | - | 973 | 2 (1) |  | 0 | 0 |
|  |  | 12-mo 24-hr feeding frequency: mean (SD) | - | - | 571 | 3 (1) | - | - | - | - |  | 0 | 0 |
|  |  | 15-mo 24-hr feeding frequency: mean (SD) | - | - | 598 | 3 (1) | - | - | - | - |  | 0 | 0 |
|  |  | 18-mo 24-hr feeding frequency: mean (SD) | - | - | 622 | 3 (1) | - | - | 1118 | 3 (1) |  | 0 | 0 |
|  |  | Mean feeding frequency across time points: mean (SD) | - | - | 664 | 2 (1) | - | - | 1121 | 3 (1) |  | D+ | 0 |
|  |  | 9-mo24-hr dietary diversity: mean (SD) | - | - | - | - | - | - | 1121 | 2^o^ (1) |  | 0 | 0 |
|  |  | 18-mo24-hr dietary diversity: mean (SD) | - | - | - | - | - | - | 1119 | 3^o^ (1) |  | 0 | 0 |
|  |  | Mean 24-hr dietary diversity: mean (SD) | - | - | - | - | - | - | 1121 | 3^o^ (1) |  | 0 | 0 |
|  |  | 9-mo 7-day dietary diversity score: mean (SD) | 968 | 3 (2) | 515 | 2 (1) | 1098 | 3 (2) | 1085 | 2^o^ (1) |  | 0 | 0 |
|  |  | 12-mo 7-day dietary diversity score: mean (SD) | 993 | 3 (2) | 532 | 3 (1) | 1049 | 3 (2) | - | - |  | 0 | 0 |
|  |  | 15-mo 7-day dietary diversity score: mean (SD) | 970 | 3 (2) | 561 | 3 (1) | 1043 | 3 (1) | - | - |  | 0 | 0 |
|  |  | 18-mo 7-day dietary diversity score: mean (SD) | 1008 | 4 (2) | 592 | 3 (1) | 1235 | 3 (2) | 1099 | 3^o^ (1) |  | 0 | 0 |
|  |  | Mean 7-day dietary diversity score: mean (SD) | 1023 | 3 (1) | 663 | 2 (1) | 1385 | 2 (1) | 1119 | 3^o^ (1) |  | A+,C+,D+ | D+ |
|  | Nurturing and Stimulation | |  |  |  |  |  |  |  |  |  |  |  |
|  |  | 6-mo HOME Total Score: mean (SD) | - | - | 641 | 24 (3) | - | - | - | - |  | B+ | 0 |
|  |  | 6-mo HOME Responsivity Score: mean (SD) | - | - | 641 | 7 (2) | - | - | - | - |  | 0 | 0 |
|  |  | 6-mo HOME Acceptance Score: mean (SD) | - | - | 641 | 6 (1) | - | - | - | - |  | 0 | 0 |
|  |  | 6-mo HOME Organization Score: mean (SD) | - | - | 641 | 4 (1) | - | - | - | - |  | 0 | 0 |
|  |  | 6-month HOME Learning Materials Score: mean (SD) | - | - | 641 | 1 (1) | - | - | - | - |  | 0 | 0 |
|  |  | 6-mo HOME Involvement Score: mean (SD) | - | - | 641 | 3 (1) | - | - | - | - |  | 0 | 0 |
|  |  | 6-mo HOME Variety Score: mean (SD) | - | - | 641 | 3 (1) | - | - | - | - |  | 0 | 0 |
|  |  | 18-moFCI Variety of Play Materials: mean (SD) | 1023 | 3 (1) | 675 | 3 (1) | 1381 | 4 (1) | 1120 | 3 (1) |  | A+,B+,C+,D+ | B+,C+,D+ |
|  |  | 18-mo FCI Activities with Caregivers: mean (SD) | 1023 | 5 (3) | 675 | 3 (3) | 1381 | 3 (2) | 1120 | 8 (3) |  | A+,C+,D+ | A+,C+,D+ |

^a^Median (SD)

^b^Association in opposite direction as expected

^c^Baseline measures were collected at < 20 wk gestation

^d^Out of Maximum 40 points

^e^Score on the Edinburgh Post-Natal Depression Scale, out of maximum 30 points

^f^Score on the Self-Reporting Questionnaire, out of maximum 20 points

^g^Meanz-score across five cognitive tests z-scores: digit span forward and backward, verbal fluency for food and people’s names, and mental rotation test.

^h^Out of Maximum 36 points

^i^Birth

^j^6 months

^k^9 months

^l^Fever in the absence of diarrhea and respiratory symptoms, which can be classified as suspected malaria cases according to the Integrated Management of ChildhoodIllnesses (IMCI) classification for high malaria risk areas.

^m^Malaria incidence

^n^Child experienced ARI during the surveillance period: n (%)

^o^Includes starchy staple foods, which were not included in the other three cohorts

Note: Where the distribution shows n(%), the binary variable was used for analysis. Where the distribution shows mean (SD), the continuous variable was used for analysis. Where the distribution shows median (SD), the log-transformed continuous variable was used for analysis.

TableS5. Variable Selection Results for Cohort A: DYAD-Ghana

|  |  | Language | | | | | |  | Motor | | | | | |
| --- | --- | --- | --- | --- | --- | --- | --- | --- | --- | --- | --- | --- | --- | --- |
|  |  | Independent association with language z-score | | | Adjusted for other variables in the same category | | |  | Independent association with motor z-score | | | Adjusted for other variables in the same category | | |
|  |  | Estimate (SE) | p-value | Decision | Estimate (SE) | p-value | Decision |  | Estimate (SE) | p-value | Decision | Estimate (SE) | p-value | Decision |
| Environmental Factors | |  |  |  |  |  |  |  |  |  |  |  |  |  |
|  | Asset Index | 0.06 (0.03) | 0.02 | retain | 0.05 (0.03) | 0.10 | retain |  | 0.04 (0.03) | 0.24 | drop | - | - | - |
|  | Household Food Insecurity Access Scale | -0.03 (0.03) | 0.29 | drop | - | - | - |  | -0.04 (0.03) | 0.21 | drop | - | - | - |
|  | Distance to Nearest Market | -0.02 (0.03) | 0.44 | drop | - | - | - |  | -0.04 (0.03) | 0.14 | drop | - | - | - |
|  | Paternal Education | 0.06 (0.03) | 0.04 | retain | 0.05 (0.03) | 0.14 | drop |  | 0.04 (0.03) | 0.23 | drop | - | - | - |
|  | Maternal Education | 0.00(0.03) | 0.86 | drop | - | - | - |  | 0.00(0.03) | 0.91 | drop | - | - | - |
|  | Unimproved water source | 0.06 (0.11) | 0.58 | drop | - | - | - |  | 0.02 (0.12) | 0.86 | drop | - | - | - |
|  | Unimproved toilet facility | 0.14 (0.17) | 0.40 | drop | - | - | - |  | 0.01 (0.20) | 0.96 | drop | - | - | - |
| Maternal Factors | |  |  |  |  |  |  |  |  |  |  |  |  |  |
|  | Maternal Age (y) | 0.14 (0.03) | <.0001 | retain | 0.12 (0.03) | <.0001 | retain |  | 0.06 (0.03) | 0.06 | drop | - | - | - |
|  | Maternal Height (cm) | 0.02 (0.03) | 0.47 | drop | - | - | - |  | 0.01 (0.03) | 0.86 | drop | - | - | - |
|  | Maternal Body Mass Index (kg/m2) | 0.04 (0.03) | 0.10 | drop | - | - | - |  | 0.07 (0.03) | 0.03 | retain | 0.06 (0.03) | 0.07 | drop |
|  | Maternal Malaria at Baseline: Positive RDT | -0.1(0.09) | 0.26 | drop | - | - | - |  | 0.11 (0.11) | 0.28 | drop | - | - | - |
|  | Maternal Baseline Hb Concentration (g/L) | 0.07 (0.04) | 0.06 | drop | - | - | - |  | 0.04 (0.04) | 0.33 | drop | - | - | - |
|  | Change in Hb (Δg/L) Baseline to 36 wk gestation | 0.07 (0.05) | 0.11 | drop | - | - | - |  | 0.05 (0.05) | 0.37 | drop | - | - | - |
|  | Change in Hb (Δg/L) 36 wk gestation to 6 mo pp | 0.01 (0.04) | 0.72 | drop | - | - | - |  | 0.01 (0.04) | 0.89 | drop | - | - | - |
|  | Maternal Baseline ZPP Concentration (μmol/molheme) | -0.02 (0.03) | 0.58 | drop | - | - | - |  | 0.04 (0.04) | 0.23 | drop | - | - | - |
|  | Change in ZPP(Δμmol/molheme) Baseline to 36 wk gestation | 0.02 (0.03) | 0.48 | drop | - | - | - |  | 0.02 (0.04) | 0.62 | drop | - | - | - |
|  | Maternal Baseline AGP | 0.04 (0.03) | 0.17 | drop | - | - | - |  | 0.05 (0.03) | 0.08 | drop | - | - | - |
|  | Maternal AGP 36 wk gestation | -0.06 (0.03) | 0.03 | retain | -0.05 (0.03) | 0.10 | drop |  | 0.04 (0.03) | 0.24 | drop | - | - | - |
|  | Maternal Elevated AGP (>=1) at both Baseline and 36 wk gestation | -0.08 (0.42) | 0.84 | drop | - | - | - |  | 0.17 (0.53) | 0.75 | drop | - | - | - |
|  | Maternal Baseline CRP | 0.02 (0.03) | 0.55 | drop | - | - | - |  | 0.08 (0.03) | 0.01 | retain | 0.07 (0.03) | 0.02 | retain |
|  | Maternal CRP 36 wk gestation | -0.04 (0.03) | 0.19 | drop | - | - | - |  | 0.01 (0.03) | 0.68 | drop | - | - | - |
|  | Maternal Elevated CRP (>=5) at both Baseline and 36 wk gestation | -0.04 (0.08) | 0.58 | drop | - | - | - |  | 0.09 (0.09) | 0.35 | drop | - | - | - |
|  | Maternal Baseline Basal Cortisol | 0 (0.03) | 0.99 | drop | - | - | - |  | -0.05 (0.03) | 0.16 | drop | - | - | - |
|  | Maternal Basal Cortisol at 36 wk gestation | -0.04 (0.03) | 0.25 | drop | - | - | - |  | -0.01 (0.03) | 0.88 | drop | - | - | - |
|  | Maternal Basal Cortisol at 6 mo pp | -0.03 (0.03) | 0.40 | drop | - | - | - |  | -0.01 (0.04) | 0.71 | drop | - | - | - |
|  | Mean Maternal Basal Cortisol across the three time points | -0.03 (0.03) | 0.40 | drop | - | - | - |  | -0.01 (0.04) | 0.71 | drop | - | - | - |
|  | Maternal Depression Score | 0 (0.01) | 0.55 | drop | - | - | - |  | 0.01 (0.01) | 0.18 | drop |  |  |  |
| Child Factors | |  |  |  |  |  |  |  |  |  |  |  |  |  |
|  | Firstborn | -0.19 (0.06) | 0.0006 | retain | -0.2 (0.07) | 0.00 | retain |  | 0 (0.06) | 0.99 | drop | - | - | - |
|  | Born Preterm | -0.19 (0.1) | 0.06 | drop | - | - | - |  | -0.21 (0.11) | 0.06 | drop | - | - | - |
|  | BMIZ at Birth | 0.09 (0.03) | 0.01 | retain | 0.06 (0.04) | 0.15 | drop |  | 0.12 (0.04) | 0.00 | retain1 | 0.1 (0.04) | 0.01 | retain |
|  | Postnatal Ponderal Growth: Change in BMIZ Birth to 18 mo | -0.02 (0.03) | 0.61 | retain1 | -0.04 (0.04) | 0.27 | drop |  | 0.1(0.04) | 0.01 | retain | 0.07 (0.04) | 0.07 | retain^1^ |
|  | LAZ at Birth | 0.11 (0.03) | 0.0004 | retain | 0.09 (0.04) | 0.02 | retain |  | 0.1(0.03) | 0.004 | retain | 0.08 (0.04) | 0.049 | retain |
|  | Postnatal Linear Growth: Change in LAZ Birth to 18 mo | 0.04 (0.03) | 0.15 | retain1 | 0.06 (0.04) | 0.11 | retain^1^ |  | 0.09 (0.03) | 0.01 | retain | 0.06 (0.04) | 0.13 | retain^1^ |
|  | Child 6-month Hb Concentration (g/L) | 0.1(0.03) | 0.002 | retain | 0.11 (0.04) | 0.005 | retain |  | 0 (0.04) | 0.98 | drop | - | - | - |
|  | Change in Hb 6 to 18 months | 0.1(0.03) | 0.005 | retain | 0.1 (0.04) | 0.01 | retain |  | 0 (0.04) | 0.97 | drop | - | - | - |
|  | Child 6-month ZPP Concentration | -0.04 (0.03) | 0.29 | retain1 | 0.02 (0.04) | 0.61 | drop |  | -0.04 (0.04) | 0.30 | drop | - | - | - |
|  | Change in ZPP 6 to 18 months | -0.07 (0.03) | 0.02 | retain | -0.03 (0.04) | 0.39 | drop |  | 0.04 (0.04) | 0.24 | drop | - | - | - |
|  | Highest Quartile Diarrhea Prevalence (>=3.5% of days) | 0.04 (0.06) | 0.46 | drop | - | - | - |  | -0.07 (0.07) | 0.32 | drop | - | - | - |
|  | Highest Quartile Fever Prevelance (>=2.5% of days) | -0.03 (0.06) | 0.68 | drop | - | - | - |  | 0.08 (0.07) | 0.25 | drop | - | - | - |
|  | ARI Prevalence (% of days) | -0.03 (0.03) | 0.18 | drop | - | - | - |  | -0.04 (0.03) | 0.23 | drop | - | - | - |
|  | Positive behavior during assessment | - | - | - | - | - | - |  | 0.6(0.06) | <.0001 | retain | 0.6(0.06) | <.0001 | retain |
| Caregiving Factors | |  |  |  |  |  |  |  |  |  |  |  |  |  |
|  | Exclusive Breastfeeding First 6 mo | -0.03 (0.06) | 0.65 | drop | - | - | - |  | -0.04 (0.07) | 0.51 | drop | - | - | - |
|  | 9-month dietary diversity score | 0.1(0.03) | 0.0003 | drop^2^ | - | - | - |  | 0.04 (0.03) | 0.24 | drop | - | - | - |
|  | 12-month dietary diversity score | 0.09 (0.03) | 0.0007 | drop^2^ | - | - | - |  | 0.09 (0.03) | 0.0026 | drop^2^ | - | - | - |
|  | 15-month dietary diversity score | 0.08 (0.03) | 0.0021 | drop^2^ | - | - | - |  | 0.05 (0.03) | 0.10 | drop | - | - | - |
|  | 18-month dietary diversity score | 0.12 (0.03) | <.0001 | drop^2^ | - | - | - |  | 0.08 (0.03) | 0.01 | drop^2^ | - | - | - |
|  | Mean dietary diversity score | 0.14 (0.03) | <.0001 | retain | 0.06 (0.02) | 0.0123 | retain |  | 0.1(0.03) | 0.001 | retain | 0.05 (0.03) | 0.07 | drop |
|  | 18-Month variety of play materials | 0.24 (0.03) | <.0001 | retain | 0.19 (0.03) | <.0001 | retain |  | 0.09 (0.03) | 0.0013 | retain | 0.04 (0.03) | 0.17 | drop |
|  | 18-Month activities with caregivers | 0.17 (0.03) | <.0001 | retain | 0.10 (0.03) | 0.0005 | retain |  | 0.13 (0.03) | <.0001 | retain | 0.11 (0.03) | 0.0002 | retain |

^1^retain as a covariate

^2^drop due to collinearity with mean dietary diversity score

TableS6. Variable Selection Results for Cohort B: DYAD-Malawi

|  |  | Language | | | | | |  | Motor | | | | | |
| --- | --- | --- | --- | --- | --- | --- | --- | --- | --- | --- | --- | --- | --- | --- |
|  |  | Independent association with language z-score | | | Adjusted for other variables in the same category | | |  | Independent association with motor z-score | | | Adjusted for other variables in the same category | | |
|  |  | Estimate (SE) | p-value | Decision | Estimate (SE) | p-value | Decision |  | Estimate (SE) | p-value | Decision | Estimate (SE) | p-value | Decision |
| Environmental Factors | |  |  |  |  |  |  |  |  |  |  |  |  |  |
|  | Asset Index | 0.13 (0.04) | 0.00 | retain | 0.05 (0.06) | 0.41 | drop |  | 0.09 (0.04) | 0.04 | retain | 0.08 (0.05) | 0.10 | drop |
|  | Household Food Insecurity Access Scale | -0.02 (0.04) | 0.58 | drop | - | - | - |  | -0.03 (0.04) | 0.49 | drop | - | - | - |
|  | Paternal Education | 0.14 (0.05) | 0.00 | retain | 0.10 (0.05) | 0.06 | retain |  | 0.14 (0.05) | 0.002 | retain | 0.12 (0.05) | 0.01 | retain |
|  | Maternal Education | 0.11 (0.04) | 0.01 | retain | 0.04 (0.05) | 0.46 | drop |  | 0.05 (0.04) | 0.29 | drop | - | - | - |
|  | Unimproved water source | -0.01 (0.15) | 0.92 | drop | - | - | - |  | -0.07 (0.14) | 0.62 | drop | - | - | - |
|  | Unimproved toilet facility | -0.32 (0.14) | 0.03 | retain | -0.15 (0.18) | 0.39 | drop |  | 0.03 (0.14) | 0.83 | drop | - | - | - |
| Maternal Factors | |  |  |  |  |  |  |  |  |  |  |  |  |  |
|  | Maternal Age (y) | 0.05 (0.04) | 0.23 | drop | - | - | - |  | 0.01 (0.04) | 0.82 | drop | - | - | - |
|  | Maternal Height (cm) | 0.11 (0.04) | 0.01 | retain | 0.12 (0.05) | 0.01 | retain |  | 0.02 (0.04) | 0.60 | drop | - | - | - |
|  | Maternal Body Mass Index (kg/m2) | 0.02 (0.04) | 0.72 | drop | - | - | - |  | 0.08 (0.04) | 0.07 | drop | - | - | - |
|  | Maternal HIV Positive | 0.02 (0.13) | 0.88 | drop | - | - | - |  | -0.12 (0.13) | 0.34 | drop | - | - | - |
|  | Maternal Malaria at Baseline: Positive RDT | 0.08 (0.10) | 0.43 | drop | - | - | - |  | -0.09 (0.1) | 0.39 | drop | - | - | - |
|  | Maternal Baseline Hb Concentration (g/L) | 0.09 (0.07) | 0.16 | drop | - | - | - |  | 0.05 (0.06) | 0.43 | drop | - | - | - |
|  | Change in Hb (Δg/L) Baseline to 36 wk gestation | 0.13 (0.07) | 0.08 | drop | - | - | - |  | 0.01 (0.07) | 0.90 | drop | - | - | - |
|  | Change in Hb (Δg/L) 36 wk gestation to 6 mo pp | 0.1(0.06) | 0.13 | drop | - | - | - |  | 0 (0.06) | 0.98 | drop | - | - | - |
|  | Maternal Baseline ZPP Concentration (μmol/molheme) | -0.12 (0.06) | 0.054 | drop | - | - | - |  | -0.08 (0.06) | 0.14 | drop | - | - | - |
|  | Change in ZPP(Δμmol/molheme) Baseline to 36 wk gestation | -0.09 (0.07) | 0.19 | drop | - | - | - |  | -0.08 (0.06) | 0.21 | drop | - | - | - |
|  | Change in ZPP(Δμmol/molheme) 36 wk gestation to 6 mo pp | -0.09 (0.06) | 0.14 | drop | - | - | - |  | -0.02 (0.06) | 0.69 | drop | - | - | - |
|  | Maternal Baseline Serum (?)Tfr | 0.06 (0.05) | 0.23 | drop | - | - | - |  | -0.01 (0.05) | 0.78 | drop | - | - | - |
|  | Change in Tfr Baseline to 36 wk gestation | 0.03 (0.05) | 0.54 | drop | - | - | - |  | -0.03 (0.05) | 0.54 | drop | - | - | - |
|  | Maternal Baseline AGP | -0.05 (0.04) | 0.20 | drop | - | - | - |  | -0.02 (0.04) | 0.64 | drop | - | - | - |
|  | Maternal AGP 36 wk gestation | -0.1(0.05) | 0.02 | retain | -0.05 (0.06) | 0.37 | drop |  | -0.03 (0.04) | 0.44 | drop | - | - | - |
|  | Maternal Elevated AGP (>=1) at both Baseline and 36 wk gestation | -0.05 (0.33) | 0.87 | drop | - | - | - |  | -0.58 (0.31) | 0.06 | drop | - | - | - |
|  | Maternal Baseline CRP | 0.01 (0.04) | 0.87 | drop | - | - | - |  | 0 (0.04) | 0.91 | drop | - | - | - |
|  | Maternal CRP 36 wk gestation | -0.09 (0.05) | 0.046 | retain | -0.04 (0.06) | 0.53 | drop |  | 0 (0.04) | 0.93 | drop | - | - | - |
|  | Maternal Elevated CRP (>=5) at both Baseline and 36 wk gestation | -0.03 (0.13) | 0.84 | drop | - | - | - |  | 0.18 (0.12) | 0.15 | drop | - | - | - |
|  | Maternal Baseline Basal Cortisol | -0.07 (0.04) | 0.09 | drop | - | - | - |  | -0.04 (0.04) | 0.35 | drop | - | - | - |
|  | Maternal Basal Cortisol at 28 wk gestation | -0.05 (0.05) | 0.37 | drop | - | - | - |  | 0.01 (0.05) | 0.80 | drop | - | - | - |
|  | Maternal Basal Cortisol at 36 wk gestation | 0.02 (0.05) | 0.77 | drop | - | - | - |  | -0.12 (0.05) | 0.01 | retain | -0.10 (0.05) | 0.04 | retain |
|  | Mean Maternal Basal Cortisol across the three time points | -0.08 (0.04) | 0.06 | drop | - | - | - |  | -0.05 (0.04) | 0.18 | drop | - | - | - |
|  | Maternal Baseline Perceived Stress Scale (PSS) score | 0.05 (0.04) | 0.22 | drop | - | - | - |  | 0.06 (0.04) | 0.15 | drop | - | - | - |
|  | Maternal PSS score at 28 wk gestation | 0.05 (0.05) | 0.32 | drop | - | - | - |  | -0.03 (0.05) | 0.55 | drop | - | - | - |
|  | Maternal PSS score at 36 wk gestation | -0.02 (0.05) | 0.67 | drop | - | - | - |  | 0.02 (0.05) | 0.64 | drop | - | - | - |
|  | Mean Maternal PSS score across the three time points | 0.04 (0.04) | 0.34 | drop | - | - | - |  | 0.04 (0.04) | 0.38 | drop | - | - | - |
|  | Maternal Depression Score | 0.09 (0.04) | 0.0503 | drop | - | - | - |  | 0.03 (0.04) | 0.47 | drop | - | - | - |
|  | Maternal Cognitive Score | 0.12 (0.04) | 0.01 | retain | 0.1 (0.05) | 0.04 | retain |  | 0.05 (0.04) | 0.20 | drop | - | - | - |
|  | Maternal Functional Health Literacy Score | 0.11 (0.04) | 0.02 | retain | 0.07 (0.05) | 0.15 | drop |  | 0.12 (0.04) | 0.01 | retain | 0.12 (0.05) | 0.01 | retain |
| Child Factors | |  |  |  |  |  |  |  |  |  |  |  |  |  |
|  | Firstborn | -0.24 (0.1) | 0.02 | retain | -0.27 (0.13) | 0.049 | retain |  | 0.05 (0.1) | 0.62 | drop | - | - | - |
|  | Born Preterm | -0.36 (0.16) | 0.02 | retain | -0.36 (0.25) | 0.15 | drop |  | -0.13 (0.16) | 0.39 | drop | - | - | - |
|  | Born Small for Gestational Age | -0.08 (0.1) | 0.42 | retain^1^ | 0.03 (0.13) | 0.79 | drop |  | -0.02 (0.09) | 0.85 | retain^1^ | 0.08 (0.11) | 0.44 | retain1 |
|  | Postnatal Ponderal Growth: Residual of SGA Predicting 18-mo WLZ | 0.1(0.04) | 0.02 | retain | 0.09 (0.05) | 0.09 | drop |  | 0.12 (0.04) | 0.002 | retain | 0.11 (0.04) | 0.01 | retain |
|  | LAZ at Birth | 0.16 (0.05) | 0.0005 | retain | 0.09 (0.07) | 0.21 | drop |  | 0.2 (0.04) | <.0001 | retain | 0.18 (0.05) | 0.0005 | retain |
|  | Postnatal Linear Growth: Change in LAZ Birth to 18 mo | 0.17 (0.05) | 0.0003 | retain | 0.1 (0.06) | 0.11 | drop |  | 0.23 (0.05) | <.0001 | retain | 0.18 (0.05) | 0.0003 | retain |
|  | Child 6-month Hb Concentration (g/L) | 0.19 (0.06) | 0.0007 | retain | 0.04 (0.08) | 0.59 | drop |  | 0.1 (0.05) | 0.06 | drop | - | - | - |
|  | Change in Hb 6 to 18 months | 0.17 (0.05) | 0.0022 | retain | 0.11 (0.07) | 0.13 | drop |  | 0.09 (0.05) | 0.08 | drop | - | - | - |
|  | Child 6-month ZPP Concentration | -0.16 (0.06) | 0.01 | retain | -0.15 (0.08) | 0.049 | retain |  | -0.06 (0.06) | 0.27 | drop | - | - | - |
|  | Change in ZPP 6 to 18 months | -0.12 (0.06) | 0.04 | retain | -0.15 (0.07) | 0.0498 | retain |  | -0.11 (0.06) | 0.06 | drop | - | - | - |
|  | Child 6-month AGP | -0.09 (0.05) | 0.045 | drop^2^ | - | - | - |  | -0.05 (0.05) | 0.29 | drop | - | - | - |
|  | Child 18-month AGP | -0.11 (0.04) | 0.01 | drop^2^ | - | - | - |  | -0.15 (0.04) | 0.001 | retain | -0.06 (0.04) | 0.16 | drop |
|  | Child Elevated AGP (>=1) at both 6 and 18 months | -0.22 (0.08) | 0.01 | retain | -0.19 (0.11) | 0.10 | drop |  | -0.19 (0.08) | 0.02 | drop^5^ | - | - | - |
|  | Child 6-month CRP | -0.05 (0.05) | 0.28 | drop | - | - | - |  | 0 (0.05) | 0.99 | drop | - | - | - |
|  | Child 18-month CRP | -0.08 (0.04) | 0.07 | drop | - | - | - |  | -0.13 (0.04) | 0.003 | drop^5^ | - | - | - |
|  | Child Elevated CRP (>=1) at both 6 and 18 months | -0.32 (0.11) | 0.004 | retain | -0.24 (0.18) | 0.19 | drop |  | -0.32 (0.11) | 0.004 | drop^5^ | - | - | - |
|  | Highest Quartile Diarrhea Prevalence (>=3.5% of days) | -0.15 (0.1) | 0.14 | drop | - | - | - |  | -0.06 (0.1) | 0.51 | drop | - | - | - |
|  | Highest Quartile Fever Prevalence (>=2.5% of days) | -0.25 (0.1) | 0.02 | retain | -0.17 (0.13) | 0.20 | drop |  | -0.21 (0.1) | 0.04 | retain | -0.11 (0.11) | 0.32 | drop |
|  | Child 6-month Basal Cortisol | -0.08 (0.05) | 0.12 | drop | - | - | - |  | -0.09 (0.05) | 0.10 | drop | - | - | - |
|  | Child 12-month Basal Cortisol | 0.04 (0.05) | 0.40 | drop | - | - | - |  | -0.05 (0.05) | 0.27 | drop | - | - | - |
|  | Child 18-month Basal Cortisol | -0.05 (0.04) | 0.29 | drop | - | - | - |  | -0.15 (0.04) | 0.00 | retain | -0.14 (0.05) | 0.003 | retain |
|  | Mean Child Basal Cortisol across the three time points | -0.04 (0.04) | 0.33 | drop | - | - | - |  | -0.14 (0.04) | 0.00 | drop^6^ | - | - | - |
|  | Accelerometer Mean Vector Magnitude | 0.07 (0.05) | 0.15 | drop | - | - | - |  | 0.12 (0.04) | 0.01 | retain | 0.12 (0.04) | 0.008 | retain |
|  | Mean % of time spent in moderate to vigorous physical activity | 0.02 (0.04) | 0.67 | drop | - | - | - |  | -0.03 (0.04) | 0.43 | drop | - | - | - |
|  | Positive behavior during assessment: n (%) | - | - | - | - | - | - |  | 0.63 (0.08) | <.0001 | retain | 0.63 (0.08) | <.0001 | retain |
| Caregiving Factors | |  |  |  |  |  |  |  |  |  |  |  |  |  |
|  | Exclusive Breastfeeding First 6 months | 0.14 (0.23) | 0.56 | drop | - | - | - |  | -0.07 (0.22) | 0.74 | drop | - | - | - |
|  | 9-month feeding frequency yesterday | 0.05 (0.05) | 0.28 | drop | - | - | - |  | 0.05 (0.05) | 0.26 | drop | - | - | - |
|  | 12-month feeding frequency yesterday | -0.04 (0.05) | 0.42 | drop | - | - | - |  | 0.02 (0.04) | 0.66 | drop | - | - | - |
|  | 15-month feeding frequency yesterday | 0.04 (0.04) | 0.32 | drop | - | - | - |  | -0.04 (0.04) | 0.41 | drop | - | - | - |
|  | 18-month feeding frequency yesterday | 0.1(0.04) | 0.02 | retain | 0.07 (0.04) | 0.09 | drop |  | 0 (0.04) | 0.99 | drop | - | - | - |
|  | Mean feeding frequency across the four time points | 0.05 (0.04) | 0.21 | drop | - | - | - |  | -0.01 (0.04) | 0.82 | drop | - | - | - |
|  | 9-month dietary diversity score | 0.13 (0.05) | 0.01 | drop^3^ | - | - | - |  | 0.04 (0.05) | 0.42 | drop | - | - | - |
|  | 12-month dietary diversity score | 0.09 (0.05) | 0.06 | drop | - | - | - |  | -0.07 (0.05) | 0.12 | drop | - | - | - |
|  | 15-month dietary diversity score | 0.06 (0.05) | 0.17 | drop | - | - | - |  | 0.06 (0.05) | 0.16 | drop | - | - | - |
|  | 18-month dietary diversity score | 0.1(0.05) | 0.02 | drop^3^ | - | - | - |  | 0.08 (0.05) | 0.07 | drop | - | - | - |
|  | Mean dietary diversity score | 0.13 (0.04) | 0.002 | retain | 0.09 (0.05) | 0.07 | drop |  | 0.03 (0.04) | 0.45 | drop | - | - | - |
|  | 6-month HOME Total Score | 0.12 (0.04) | 0.004 | retain | 0.12 (0.04) | 0.004 | retain |  | 0.05 (0.04) | 0.29 | drop | - | - | - |
|  | 6-month HOME Responsivity Sub-Scale Score | 0 (0.04) | 0.94 | drop | - | - | - |  | 0.01 (0.04) | 0.77 | drop | - | - | - |
|  | 6-month HOME Acceptance Sub-Scale Score | 0.04 (0.04) | 0.32 | drop | - | - | - |  | -0.08 (0.04) | 0.06 | drop | - | - | - |
|  | 6-month HOME Organization Sub-Scale Score | 0.11 (0.04) | 0.01 | drop^4^ | - | - | - |  | 0.08 (0.04) | 0.07 | drop | - | - | - |
|  | 6-month HOME Learning Materials Sub-Scale Score | 0.11 (0.05) | 0.02 | drop^4^ | - | - | - |  | 0.1 (0.05) | 0.04 | retain | 0.1 (0.05) | 0.02 | retain |
|  | 6-month HOME Involvement Sub-Scale Score | 0.1(0.05) | 0.04 | drop^4^ | - | - | - |  | 0.03 (0.05) | 0.45 | drop | - | - | - |
|  | 6-month HOME Variety Sub-Scale Score | 0.11 (0.04) | 0.01 | drop^4^ | - | - | - |  | 0 (0.04) | 0.95 | drop | - | - | - |
|  | 18-Month variety of play materials | 0.4(0.05) | <.0001 | retain | 0.36 (0.06) | <.0001 | retain |  | 0.18 (0.05) | 0.0003 | retain | 0.17 (0.05) | 0.0005 | retain |
|  | 18-Month activities with caregivers | 0.18 (0.06) | 0.002 | retain | 0.07 (0.06) | 0.22 | drop |  | 0.07 (0.05) | 0.15 | drop | - | - | - |

^1^retain as a covariate

^2^drop due to collinearity with high AGP at both time points

^3^drop due to collinearity with mean dietary diversity score

^4^drop due to collinearity with total home score

^5^drop due to collinearity with 18-mo AGP

^6^drop due to collinearity with 18-mo basal cortisol

TableS7. Variable Selection Results for Cohort C: DOSE-Malawi

|  |  | Language | | | | | |  | Motor | | | | | |
| --- | --- | --- | --- | --- | --- | --- | --- | --- | --- | --- | --- | --- | --- | --- |
|  |  | Independent association with language z-score | | | Adjusted for other variables in the same category | | |  | Independent association with motor z-score | | | Adjusted for other variables in the same category | | |
|  |  | Estimate (SE) | p-value | Decision | Estimate (SE) | p-value | Decision |  | Estimate (SE) | p-value | Decision | Estimate (SE) | p-value | Decision |
| Environmental Factors | |  |  |  |  |  |  |  |  |  |  |  |  |  |
|  | Asset Index | 0.07 (0.03) | 0.02 | retain | 0.03 (0.04) | 0.45 | drop |  | 0.14 (0.03) | <.0001 | retain | 0.06 (0.04) | 0.09 | drop |
|  | Household Food Insecurity Access Scale | -0.07 (0.03) | 0.03 | retain | -0.04 (0.03) | 0.23 | drop |  | -0.07 (0.03) | 0.02 | retain | -0.02 (0.03) | 0.54 | drop |
|  | Distance to Nearest Market | 0.01 (0.03) | 0.61 | drop | - | - | - |  | -0.14 (0.03) | <.0001 | retain | -0.08 (0.03) | 0.01 | retain |
|  | Paternal Education | 0.08 (0.03) | 0.01 | retain | 0.06 (0.04) | 0.13 | retain |  | 0.13 (0.03) | <.0001 | retain | 0.08 (0.03) | 0.02 | retain |
|  | Maternal Education | 0.09 (0.03) | 0.00 | retain | 0.04 (0.04) | 0.36 | drop |  | 0.14 (0.03) | <.0001 | retain | 0.05 (0.04) | 0.15 | drop |
|  | Unimproved water source | -0.1(0.11) | 0.39 | drop | - | - | - |  | -0.26 (0.11) | 0.01 | retain | -0.25 (0.11) | 0.03 | retain |
|  | Unimproved toilet facility | -0.05 (0.21) | 0.82 | drop | - | - | - |  | -0.48 (0.2) | 0.02 | retain | 0.12 (0.23) | 0.61 | drop |
| Maternal Factors | |  |  |  |  |  |  |  |  |  |  |  |  |  |
|  | Maternal Age | -0.04 (0.03) | 0.20 | drop | - | - | - |  | -0.03 (0.03) | 0.23 | drop | - | - | - |
|  | Maternal Height | 0.04 (0.03) | 0.20 | drop | - | - | - |  | 0.08 (0.03) | 0.002 | retain | 0.08 (0.03) | 0.00 | retain |
|  | Maternal Body Mass Index | 0 (0.03) | 0.94 | drop | - | - | - |  | 0.05 (0.03) | 0.08 | drop | - | - | - |
| Child Factors | |  |  |  |  |  |  |  |  |  |  |  |  |  |
|  | Child 6-month Hb Concentration | 0.06 (0.03) | 0.03 | retain | 0.04 (0.04) | 0.37 | drop |  | 0.07 (0.03) | 0.01 | retain | 0.02 (0.04) | 0.52 | drop |
|  | Child 6-month ZPP Concentration | -0.08 (0.04) | 0.03 | retain | -0.06 (0.04) | 0.13 | drop |  | -0.12 (0.03) | 0.001 | retain | -0.11 (0.04) | 0.004 | retain |
|  | Change in ZPP 6 to 18 months | -0.03 (0.04) | 0.46 | retain^1^ | -0.03 (0.04) | 0.40 | drop |  | -0.08 (0.03) | 0.02 | retain | -0.07 (0.03) | 0.03 | retain |
|  | Child 6-month LAZ | 0.16 (0.03) | <.0001 | retain | 0.12 (0.04) | 0.00 | retain |  | 0.22 (0.03) | <.0001 | retain | 0.2 (0.03) | <.0001 | retain |
|  | Change in LAZ 6 to 18 months | 0.12 (0.04) | 0.002 | retain | 0.08 (0.05) | 0.08 | retain^1^ |  | 0.31 (0.04) | <.0001 | retain | 0.27 (0.04) | <.0001 | retain |
|  | Child 6-month WLZ | 0.05 (0.03) | 0.12 | retain^1^ | 0.01 (0.04) | 0.74 | drop |  | 0.1(0.03) | 0.000 | retain | 0.03 (0.03) | 0.34 | retain^1^ |
|  | Change in WLZ 6 to 18 months | 0.08 (0.03) | 0.03 | retain | 0.05 (0.04) | 0.25 | drop |  | 0.12 (0.03) | <.0001 | retain | 0.09 (0.04) | 0.01 | retain |
|  | Diarrhea Prevalence | -0.03 (0.03) | 0.25 | drop | - | - | - |  | 0 (0.03) | 0.97 | drop | - | - | - |
|  | Diarrhea Incidence | -0.01 (0.03) | 0.73 | drop | - | - | - |  | -0.04 (0.03) | 0.09 | drop | - | - | - |
|  | Fever Prevelance | -0.05 (0.03) | 0.06 | drop | - | - | - |  | -0.02 (0.03) | 0.49 | drop | - | - | - |
|  | Undefined Fever Incidence | -0.02 (0.03) | 0.44 | drop | - | - | - |  | -0.06 (0.03) | 0.02 | retain | -0.05 (0.03) | 0.07 | drop |
|  | ARI Incidence | 0.01 (0.03) | 0.61 | drop | - | - | - |  | 0.05 (0.03) | 0.08 | drop | - | - | - |
|  | Accelerometer Mean Vector Magnitude | 0.06 (0.03) | 0.07 | drop | - | - | - |  | 0.12 (0.03) | <.0001 | retain | 0.12 (0.03) | <.0001 | retain |
|  | Mean % of time spent in moderate to vigorous physical activity | -0.02 (0.03) | 0.46 | drop | - | - | - |  | -0.05 (0.03) | 0.12 | drop | - | - | - |
|  | Positive behavior during assessment | - | - | - | - | - | - |  | 0.62 (0.06) | <.0001 | retain | 0.13 (0.03) | <.0001 | retain |
| Caregiving Factors | |  |  |  |  |  |  |  |  |  |  |  |  |  |
|  | 9-month dietary diversity score | 0.11 (0.03) | 0.00 | drop^2^ | - | - | - |  | 0.04 (0.03) | 0.19 | drop | - | - | - |
|  | 12-month dietary diversity score | 0.06 (0.03) | 0.05 | drop^2^ | - | - | - |  | 0.05 (0.03) | 0.11 | drop | - | - | - |
|  | 15-month dietary diversity score | 0.10(0.03) | 0.00 | drop^2^ | - | - | - |  | 0.07 (0.03) | 0.02 | drop^1^ | - | - | - |
|  | 18-month dietary diversity score | 0.09 (0.03) | 0.00 | drop^2^ | - | - | - |  | 0.02 (0.03) | 0.42 | drop | - | - | - |
|  | Mean dietary diversity score | 0.15 (0.03) | <.0001 | retain | 0.13 (0.03) | <.0001 | retain |  | 0.07 (0.03) | 0.01 | retain | 0.06 (0.03) | 0.08 | drop |
|  | 18-Month variety of play materials | 0.28 (0.03) | <.0001 | retain | 0.23 (0.03) | <.0001 | retain |  | 0.11 (0.03) | 0.00 | retain | 0.06 (0.03) | 0.04 | retain |
|  | 18-Month activities with caregivers | 0.18 (0.03) | <.0001 | retain | 0.09 (0.04) | 0.01 | retain |  | 0.13 (0.03) | <.0001 | retain | 0.1 (0.03) | 0.001 | retain |

^1^retain as covariate

^2^drop due to collinearity with mean dietary diversity score

TableS8. Variable Selection Results for Cohort D: ZINC-Burkina Faso

|  |  | Language | | | | | |  | Motor | | | | | |
| --- | --- | --- | --- | --- | --- | --- | --- | --- | --- | --- | --- | --- | --- | --- |
|  |  | Independent association with language z-score | | | Adjusted for other variables in the same category | | |  | Independent association with motor z-score | | | Adjusted for other variables in the same category | | |
|  |  | Estimate (SE) | p-value | Decision | Estimate (SE) | p-value | Decision |  | Estimate (SE) | p-value | Decision | Estimate (SE) | p-value | Decision |
| Environmental Factors | |  |  |  |  |  |  |  |  |  |  |  |  |  |
|  | Asset Index | 0.05 (0.03) | 0.07 | drop | - | - | - |  | 0.12 (0.03) | <.0001 | retain | 0.11 (0.03) | 0.00 | retain |
|  | Household Food Insecurity Access Scale | -0.02 (0.07) | 0.73 | drop | - | - | - |  | -0.03 (0.07) | 0.70 | drop | - | - | - |
|  | Distance to Nearest Market | 0.06 (0.02) | 0.01 | retain | 0.08 (0.03) | 0.01 | retain |  | 0.03 (0.02) | 0.27 | drop | - | - | - |
|  | Father no education | -0.02 (0.06) | 0.81 | drop | - | - | - |  | -0.01 (0.06) | 0.92 | drop | - | - | - |
|  | Mother no education | -0.10(0.06) | 0.08 | drop | - | - | - |  | -0.07 (0.06) | 0.21 | drop | - | - | - |
|  | Unimproved water source | -0.19 (0.07) | <0.01 | retain | -0.21 (0.07) | 0.002 | retain |  | -0.21 (0.07) | 0.002 | retain | -0.13 (0.07) | 0.054 | drop |
|  | Unimproved toilet facility | 0.02 (0.19) | 0.91 | drop | - | - | - |  | -0.17 (0.19) | 0.37 | drop | - | - | - |
| Maternal Factors | |  |  |  |  |  |  |  |  |  |  |  |  |  |
|  | Maternal Age | 0.00(0.00) | 0.68 | drop | - | - | - |  | 0.01 (0) | 0.07 | drop | - | - | - |
|  | Maternal Height | 0.01 (0.01) | 0.04 | retain | 0.06 (0.03) | 0.04 | retain |  | 0.01 (0.01) | 0.004 | retain | 0.08 (0.03) | 0.01 | retain |
|  | Maternal Body Mass Index | 0.02 (0.01) | 0.17 | drop | - | - | - |  | 0.04 (0.01) | 0.0002 | retain | 0.11 (0.03) | 0.000 | retain |
| Child Factors | |  |  |  |  |  |  |  |  |  |  |  |  |  |
|  | Firstborn | -0.13 (0.08) | 0.08 | drop | - | - | - |  | -0.13 (0.08) | 0.09 | drop | - | - | - |
|  | Child 9-month Hb Concentration (g/L) | 0.15 (0.04) | <0.01 | retain | 0.08 (0.04) | 0.06 | drop |  | 0.23 (0.04) | <.0001 | retain | 0.10 (0.05) | 0.03 | retain |
|  | Change in Hb 9 to 18 months | 0.05 (0.04) | 0.18 | retain^1^ | 0.02 (0.04) | 0.64 | drop |  | 0.20(0.04) | <.0001 | retain | 0.15 (0.04) | 0.0006 | retain |
|  | Child 9-month ZPP Concentration | -0.11 (0.03) | <0.01 | retain | -0.1 (0.03) | 0.002 | retain |  | -0.11 (0.03) | <0.01 | retain | -0.12 (0.04) | 0.0007 | retain |
|  | Child 9-month LAZ | 0.18 (0.03) | <.0001 | retain | 0.15 (0.03) | <.0001 | retain |  | 0.35 (0.03) | <.0001 | retain | 0.27 (0.03) | <.0001 | retain |
|  | Change in LAZ 9 to 18 months | 0.25 (0.05) | <.0001 | retain | 0.23 (0.05) | <.0001 | retain |  | 0.47 (0.05) | <.0001 | retain | 0.38 (0.06) | <.0001 | retain |
|  | Child 9-month WLZ | 0.14 (0.03) | <.0001 | retain | 0.08 (0.03) | 0.01 | retain |  | 0.27 (0.03) | <.0001 | retain | 0.12 (0.04) | 0.001 | retain |
|  | Change in WLZ 9 to 18 months | 0.06 (0.04) | 0.18 | retain^1^ | 0.03 (0.04) | 0.43 | retain^1^ |  | 0.11 (0.04) | 0.01 | retain | 0.01 (0.05) | 0.92 | retain^1^ |
|  | Diarrhea Prevalence (% of days) | 1.89 (1.21) | 0.12 | drop | - | - | - |  | 0.01 (0.05) | 0.83 | drop | - | - | - |
|  | Diarrhea Incidence (episodes per 100 d) | 0.15 (0.08) | 0.06 | drop | - | - | - |  | 0.07 (0.08) | 0.38 | drop | - | - | - |
|  | Fever Prevalence (% of days) | 3.34 (1.34) | 0.01 | retain | 0.06 (0.03) | 0.09 | drop |  | 0.00(0.06) | 0.94 | drop | - | - | - |
|  | Malaria incidence (episodes per 100 d) | -0.14 (0.1) | 0.13 | drop | - | - | - |  | -0.20(0.1) | 0.03 | retain | -0.06 (0.03) | 0.06 | drop |
|  | Child had ARI during surveillance period | -0.02 (0.07) | 0.76 | drop | - | - | - |  | -0.05 (0.07) | 0.49 | drop | - | - | - |
| Caregiving Factors | |  |  |  |  |  |  |  |  |  |  |  |  |  |
|  | 9-month meal frequency in past 24 hours | 0.11 (0.03) | 0.00 | drop^2^ | - | - | - |  | 0.04 (0.03) | 0.14 | drop | - | - | - |
|  | 18-month meal frequency in past 24 hours | 0.12 (0.03) | <.0001 | drop^2^ | - | - | - |  | 0.08 (0.03) | 0.00 | drop^2^ | - | - | - |
|  | Mean meal frequency in past 24 hours | 0.20(0.04) | <.0001 | retain | 0.08 (0.03) | 0.01 | retain |  | 0.11 (0.04) | 0.00 | retain | -0.01 (0.03) | 0.63 | drop |
|  | 9-month dietary diversity in past 24 hours | 0.09 (0.02) | 0.00 | drop^2^ | - | - | - |  | 0.09 (0.02) | <.0001 | drop^2^ | - | - | - |
|  | 18-month dietary diversity in past 24 hours | 0.08 (0.03) | 0.00 | drop^2^ | - | - | - |  | 0.07 (0.03) | 0.01 | drop^2^ | - | - | - |
|  | Mean dietary diversity in past 24 hours | 0.15 (0.03) | <.0001 | drop^3^ | - | - | - |  | 0.14 (0.03) | <.0001 | drop^3^ | - | - | - |
|  | 9-month dietary diversity in past week | 0.09 (0.02) | <.0001 | drop^2^ | - | - | - |  | 0.09 (0.02) | <.0001 | drop^2^ | - | - | - |
|  | 18-month dietary diversity in past week | 0.10(0.02) | <.0001 | drop^2^ | - | - | - |  | 0.08 (0.03) | <0.01 | drop^2^ | - | - | - |
|  | Mean dietary diversity in past 7 days | 0.17 (0.03) | <.0001 | retain | 0.08 (0.03) | 0.01 | retain |  | 0.14 (0.03) | <.0001 | retain | 0.08 (0.03) | 0.005 | retain |
|  | 18-Month variety of play materials | 0.30(0.03) | <.0001 | retain | 0.23 (0.03) | <.0001 | retain |  | 0.40(0.03) | <.0001 | retain | 0.37 (0.03) | <.0001 | retain |
|  | 18-Month activities with caregivers | 0.23 (0.03) | <.0001 | retain | 0.14 (0.03) | <.0001 | retain |  | 0.20(0.03) | <.0001 | retain | 0.07 (0.03) | 0.009 | retain |

^1^retain as covariate

^2^drop due to collinearity with the mean of both time points

^3^drop due to collinearity with the mean dietary diversity in past 7 days

TableS9. Pathway Selection Results for Cohort A: DYAD-Ghana

| Dependent Variable | Independent Variable (IV) | Potential Mediator | Association between IV and Potential Mediator | | | Indirect effect in Multiple Mediation Model | | |
| --- | --- | --- | --- | --- | --- | --- | --- | --- |
|  |  |  | Estimate (SE) | p-value | Decision | Estimate (SE) | p-value | Decision |
| Language Z-Score | |  |  |  |  |  |  |  |
|  | Asset Index | |  |  |  |  |  |  |
|  |  | Maternal Age | 0.15 (0.03) | <.0001 | retain | 0.015 (0.006) | 0.01 | retain |
|  |  | Child Firstborn | 0.05 (0.07) | 0.45 | drop | - | - | - |
|  |  | LAZ at Birth | 0.1(0.03) | <0.01 | retain | 0.01 (0.005) | 0.046 | retain |
|  |  | Postnatal Linear Growth | -0.01 (0.03) | 0.67 | drop | - | - | - |
|  |  | Child 6-month Hb Concentration (g/L) | 0.09 (0.03) | 0.01 | retain | 0.009 (0.005) | 0.07 | drop |
|  |  | Change in Hb 6 to 18 months | 0 (0.04) | 0.94 | drop | - | - | - |
|  |  | Mean dietary diversity score | 0.18 (0.03) | <.0001 | retain | 0.014 (0.006) | 0.03 | retain |
|  |  | 18-Month variety of play materials | 0.19 (0.03) | <.0001 | retain | 0.026 (0.009) | <0.0001 | retain |
|  |  | 18-Month activities with caregivers | 0.19 (0.04) | <.0001 | retain | 0.014 (0.006) | 0.02 | retain |
|  | Maternal Age | |  |  |  |  |  |  |
|  |  | Child Firstborn | -1.39 (0.11) | <.0001 | retain | 0.03 (0.02) | 0.13 | drop |
|  |  | LAZ at Birth | 0.1(0.03) | <0.01 | retain | 0.011 (0.005) | 0.04 | retain |
|  |  | Postnatal Linear Growth | -0.07 (0.03) | 0.03 | retain | -0.005 (0.004) | 0.16 | drop |
|  |  | Child 6-month Hb Concentration (g/L) | 0.06 (0.03) | 0.09 | drop | - | - | - |
|  |  | Change in Hb 6 to 18 months | 0.06 (0.04) | 0.09 | drop | - | - | - |
|  |  | Mean dietary diversity score | 0.11 (0.03) | <0.01 | retain | 0.011 (0.005) | 0.02 | retain |
|  |  | 18-Month variety of play materials | 0.09 (0.03) | <0.01 | retain | 0.017 (0.006) | <0.01 | retain |
|  |  | 18-Month activities with caregivers | 0.05 (0.04) | 0.16 | drop | - | - | - |
|  | Child Firstborn | |  |  |  |  |  |  |
|  |  | Mean dietary diversity score | 0.1(0.07) | 0.16 | drop | - | - | - |
|  |  | 18-Month variety of play materials | -0.04 (0.07) | 0.58 | drop | - | - | - |
|  |  | 18-Month activities with caregivers | 0.23 (0.07) | <0.01 | retain | 0.012 (0.006) | 0.03 | retain |
|  | Mean dietary diversity score | |  |  |  |  |  |  |
|  |  | Postnatal Linear Growth | -0.06 (0.03) | 0.06 | drop | - | - | - |
|  |  | Change in Hb 6 to 18 months | -0.01 (0.03) | 0.88 | drop | - | - | - |
|  | LAZ at Birth | |  |  |  |  |  |  |
|  |  | 18-Month variety of play materials | 0.07 (0.04) | 0.07 | drop | - | - | - |
|  |  | 18-Month activities with caregivers | 0.03 (0.04) | 0.52 | drop | - | - | - |
|  | Postnatal Linear Growth | |  |  |  |  |  |  |
|  |  | 18-Month variety of play materials | -0.04 (0.04) | 0.31 | drop | - | - | - |
|  |  | 18-Month activities with caregivers | 0.03 (0.04) | 0.43 | drop | - | - | - |
|  | Child 6-month Hb Concentration (g/L) | |  |  |  |  |  |  |
|  |  | 18-Month variety of play materials | 0.03 (0.04) | 0.54 | drop | - | - | - |
|  |  | 18-Month activities with caregivers | 0.10(0.05) | 0.04 | retain | 0.014 (0.007) | 0.06 | drop |
|  | Change in Hb 6 to 18 months | |  |  |  |  |  |  |
|  |  | 18-Month variety of play materials | 0.04 (0.04) | 0.37 | drop | - | - | - |
|  |  | 18-Month activities with caregivers | 0.09 (0.05) | 0.06 | drop | - | - | - |
| Motor Z-Score | |  |  |  |  |  |  |  |
|  | Maternal Baseline CRP | |  |  |  |  |  |  |
|  |  | Born Small for Gestational Age | 0.05 (0.03) | 0.11 | drop | - | - | - |
|  |  | Postnatal Ponderal Growth | -0.03 (0.03) | 0.40 | drop | - | - | - |
|  |  | LAZ at Birth | 0.01 (0.03) | 0.80 | drop | - | - | - |
|  |  | Postnatal Linear Growth | -0.08 (0.03) | 0.01 | retain | -0.009 (0.005) | 0.85 | drop |
|  |  | 18-Month activities with caregivers | 0.02 (0.04) | 0.67 | drop | - | - | - |
|  |  | Positive behavior during assessment | 0.13 (0.07) | 0.06 | drop | - | - | - |
|  | BMIZ at Birth | |  |  |  |  |  |  |
|  |  | 18-Month activities with caregivers | 0.01 (0.05) | 0.78 | drop | - | - | - |
|  |  | Positive behavior during assessment | 0 (0.09) | 0.99 | drop | - | - | - |
|  | Postnatal Ponderal Growth | |  |  |  |  |  |  |
|  |  | 18-Month activities with caregivers | 0.03 (0.05) | 0.53 | drop | - | - | - |
|  |  | Positive behavior during assessment | -0.09 (0.07) | 0.20 | drop | - | - | - |
|  | LAZ at Birth | |  |  |  |  |  |  |
|  |  | 18-Month activities with caregivers | 0.03 (0.04) | 0.52 | drop | - | - | - |
|  |  | Positive behavior during assessment | 0.06 (0.08) | 0.44 | drop | - | - | - |
|  | Postnatal Linear Growth | |  |  |  |  |  |  |
|  |  | 18-Month activities with caregivers | 0.03 (0.04) | 0.43 | drop | - | - | - |
|  |  | Positive behavior during assessment | -0.16 (0.08) | 0.06 | drop | - | - | - |

TableS10. Pathway Selection Results for Cohort B: DYAD-Malawi

| Dependent Variable | Independent Variable (IV) | Potential Mediator | Association between IV and Potential Mediator | | | Indirect effect in Multiple Mediation Model | | |
| --- | --- | --- | --- | --- | --- | --- | --- | --- |
|  | |  | Estimate (SE) | p-value | Decision | Estimate (SE) | p-value | Decision |
| Language Z-Score | |  |  |  |  |  |  |  |
|  | Paternal Education | |  |  |  |  |  |  |
|  |  | Maternal Height | 0.15 (0.04) | <.0001 | retain | 0.012 (0.006) | 0.07 | drop |
|  |  | Maternal Cognitive Score | 0.32 (0.04) | <.0001 | retain | 0.017 (0.013) | 0.19 | drop |
|  |  | Child 6-month ZPP Concentration | 0.04 (0.04) | 0.32 | drop | - | - | - |
|  |  | Change in ZPP 6 to 18 months | -0.07 (0.04) | 0.10 | drop | - | - | - |
|  |  | 6-month HOME Total Score | 0.2(0.04) | <.0001 | retain | 0.016 (0.009) | 0.09 | drop |
|  |  | 18-Month variety of play materials | 0.1(0.04) | 0.01 | retain | 0.023 (0.01) | 0.03 | retain |
|  | Maternal Height | |  |  |  |  |  |  |
|  |  | Child 6-month ZPP Concentration | -0.09 (0.04) | 0.03 | retain | 0.005 (0.005) | 0.28 | drop |
|  |  | Change in ZPP 6 to 18 months | 0.04 (0.04) | 0.38 | drop | - | - | - |
|  |  | 6-month HOME Total Score | 0.06 (0.04) | 0.13 | drop | - | - | - |
|  |  | 18-Month variety of play materials | 0.03 (0.04) | 0.36 | drop | - | - | - |
|  | Maternal Cognitive Score | |  |  |  |  |  |  |
|  |  | Child 6-month ZPP Concentration | -0.02 (0.04) | 0.58 | drop | - | - | - |
|  |  | Change in ZPP 6 to 18 months | 0.03 (0.05) | 0.54 | drop | - | - | - |
|  |  | 6-month HOME Total Score | 0.25 (0.04) | <.0001 | retain | 0.021 (0.01) | 0.04 | retain |
|  |  | 18-Month variety of play materials | 0.05 (0.04) | 0.18 | drop | - | - | - |
|  | Firstborn |  |  |  |  |  |  |  |
|  |  | 6-month HOME Total Score | 0.15 (0.1) | 0.12 | drop | - | - | - |
|  |  | 18-Month variety of play materials | -0.14 (0.09) | 0.13 | drop | - | - | - |
|  | Child 6-month ZPP Concentration | |  |  |  |  |  |  |
|  |  | 6-month HOME Total Score | 0 (0.06) | 0.94 | drop | - | - | - |
|  |  | 18-Month variety of play materials | -0.16 (0.05) | 0.002 | retain | -0.025 (0.011) | 0.54 | drop |
|  | Change in ZPP 6 to 18 months | |  |  |  |  |  |  |
|  |  | 6-month HOME Total Score | 0.04 (0.06) | 0.47 | drop | - | - | - |
|  |  | 18-Month variety of play materials | -0.05 (0.05) | 0.29 | drop | - | - | - |
| Motor Z-Score | |  |  |  |  |  |  |  |
|  | Paternal Education | |  |  |  |  |  |  |
|  |  | Maternal Basal Cortisol at 36 wk gestation | 0.11 (0.05) | 0.02 | retain | -0.015 (0.01) | 0.13 | drop |
|  |  | Maternal Functional Health Literacy Score | 0.26 (0.04) | <.0001 | retain | 0.018 (0.013) | 0.18 | drop |
|  |  | Postnatal Ponderal Growth | 0.1(0.05) | 0.03 | retain | 0.012 (0.009) | 0.22 | drop |
|  |  | LAZ at Birth | 0.05 (0.05) | 0.32 | drop | - | - | - |
|  |  | Postnatal Linear Growth | 0.07 (0.05) | 0.15 | drop | - | - | - |
|  |  | Child 18-month Basal Cortisol | -0.05 (0.04) | 0.27 | drop | - | - | - |
|  |  | Accelerometer Mean Vector Magnitude | 0.03 (0.04) | 0.47 | drop | - | - | - |
|  |  | Positive behavior during assessment: n (%) | -0.01 (0.02) | 0.52 | drop | - | - | - |
|  |  | 6-month HOME Learning Materials Sub-Scale Score | 0.26 (0.04) | <.0001 | retain | 0.013 (0.015) | 0.38 | drop |
|  |  | 18-Month variety of play materials | 0.1(0.04) | 0.01 | retain | 0.013 (0.01) | 0.21 | drop |
|  | Maternal Basal Cortisol at 36 wk gestation | |  |  |  |  |  |  |
|  |  | Postnatal Ponderal Growth | 0.03 (0.05) | 0.51 | drop | - | - | - |
|  |  | LAZ at Birth | -0.11 (0.05) | 0.04 | retain | -0.007 (0.007) | 0.30 | drop |
|  |  | Postnatal Linear Growth | 0.09 (0.05) | 0.07 | drop | - | - | - |
|  |  | Child 18-month Basal Cortisol | 0.09 (0.05) | 0.08 | drop | - | - | - |
|  |  | Accelerometer Mean Vector Magnitude | 0.02 (0.05) | 0.68 | drop | - | - | - |
|  |  | Positive behavior during assessment: n (%) | -0.15 (0.1) | 0.12 | drop | - | - | - |
|  |  | 6-month HOME Learning Materials Sub-Scale Score | 0.08 (0.05) | 0.12 | drop | - | - | - |
|  |  | 18-Month variety of play materials | 0 (0.05) | 0.98 | drop | - | - | - |
|  | Maternal Functional Health Literacy Score | |  |  |  |  |  |  |
|  |  | Postnatal Ponderal Growth | -0.01 (0.04) | 0.89 | drop | - | - | - |
|  |  | LAZ at Birth | 0.08 (0.05) | 0.10 | drop | - | - | - |
|  |  | Postnatal Linear Growth | -0.05 (0.05) | 0.32 | drop | - | - | - |
|  |  | Child 18-month Basal Cortisol | 0 (0.04) | 0.95 | drop | - | - | - |
|  |  | Accelerometer Mean Vector Magnitude | 0.04 (0.04) | 0.32 | drop | - | - | - |
|  |  | Positive behavior during assessment: n (%) | -0.09 (0.08) | 0.28 | drop | - | - | - |
|  |  | 6-month HOME Learning Materials Sub-Scale Score | 0.11 (0.04) | 0.01 | retain | 0.008 (0.005) | 0.15 | drop |
|  |  | 18-Month variety of play materials | 0.10(0.04) | 0.01 | retain | 0.009 (0.006) | 0.10 | drop |
|  | Postnatal Ponderal Growth | |  |  |  |  |  |  |
|  |  | Child 18-month Basal Cortisol | 0.03 (0.04) | 0.43 | drop | - | - | - |
|  |  | Accelerometer Mean Vector Magnitude | -0.03 (0.04) | 0.50 | drop | - | - | - |
|  |  | Positive behavior during assessment: n (%) | 0.17 (0.08) | 0.03 | retain | 0.029 (0.014) | 0.03 | retain |
|  |  | 6-month HOME Learning Materials Sub-Scale Score | 0.03 (0.04) | 0.52 | drop | - | - | - |
|  |  | 18-Month variety of play materials | 0 (0.04) | 0.92 | drop | - | - | - |
|  | LAZ at Birth |  |  |  |  |  |  |  |
|  |  | Child 18-month Basal Cortisol | 0.02 (0.04) | 0.71 | drop | - | - | - |
|  |  | Accelerometer Mean Vector Magnitude | 0.01 (0.05) | 0.89 | drop | - | - | - |
|  |  | Positive behavior during assessment: n (%) | 0.07 (0.07) | 0.35 | drop | - | - | - |
|  |  | 6-month HOME Learning Materials Sub-Scale Score | 0.14 (0.04) | 0.00 | retain | 0.01 (0.007) | 0.18 | drop |
|  |  | 18-Month variety of play materials | 0.12 (0.04) | 0.00 | retain | 0.017 (0.008) | 0.04 | retain |
|  | Postnatal Linear Growth | |  |  |  |  |  |  |
|  |  | Child 18-month Basal Cortisol | 0.02 (0.05) | 0.65 | drop | - | - | - |
|  |  | Accelerometer Mean Vector Magnitude | 0.07 (0.05) | 0.16 | drop | - | - | - |
|  |  | Positive behavior during assessment | 0.11 (0.08) | 0.15 | drop | - | - | - |
|  |  | 6-month HOME Learning Materials Sub-Scale Score | 0.02 (0.04) | 0.62 | drop | - | - | - |
|  |  | 18-Month variety of play materials | 0.04 (0.04) | 0.36 | drop | - | - | - |

TableS11. Pathway Selection Results for Cohort C: DOSE-Malawi

| Dependent Variable | Independent Variable (IV) | Potential Mediator | Association between IV and Potential Mediator | | | Indirect effect in Multiple Mediation Model | | |
| --- | --- | --- | --- | --- | --- | --- | --- | --- |
|  |  |  | Estimate (SE) | p-value | Decision | Estimate (SE) | p-value | Decision |
| Language Z-Score | |  |  |  |  |  |  |  |
|  | Paternal Education | |  |  |  |  |  |  |
|  |  | Child 6-month LAZ | 0.08 (0.03) | 0.004 | retain | 0.013 (0.004) | 0.0000 | retain |
|  |  | Change in LAZ 6 to 18 months | 0.05 (0.02) | 0.02 | retain | 0.005 (0.003) | 0.07 | drop |
|  |  | Accelerometer Mean Vector Magnitude | -0.03 (0.03) | 0.31 | drop | - | - | - |
|  |  | Mean dietary diversity score | 0.13 (0.02) | <.0001 | retain | 0.016 (0.005) | 0.0000 | retain |
|  |  | 18-Month variety of play materials | 0.15 (0.02) | <.0001 | retain | 0.022 (0.006) | 0.0000 | retain |
|  |  | 18-Month activities with caregivers | 0.16 (0.02) | <.0001 | retain | 0.012 (0.005) | 0.02 | retain |
|  | Mean Dietary Diversity | |  |  |  |  |  |  |
|  |  | Change in LAZ 6 to 18 months | -0.02 (0.02) | 0.42 | drop | - | - | - |
|  | Child 6-month LAZ | |  |  |  |  |  |  |
|  |  | 18-Month variety of play materials | 0.01 (0.02) | 0.76 | drop | - | - | - |
|  |  | 18-Month activities with caregivers | 0.03 (0.02) | 0.21 | drop | - | - | - |
|  | Change in LAZ 6 to 18 months | |  |  |  |  |  |  |
|  |  | 18-Month variety of play materials | 0.04 (0.03) | 0.24 | drop | - | - | - |
|  |  | 18-Month activities with caregivers | 0.04 (0.03) | 0.28 | drop | - | - | - |
| Motor Z-Score | |  |  |  |  |  |  |  |
|  | Distance to Nearest Market | |  |  |  |  |  |  |
|  |  | Maternal Height | -0.08 (0.03) | 0.01 | retain | 0 (0.002) | 0.84 | drop |
|  |  | Child 6-month ZPP Concentration | 0.02 (0.03) | 0.37 | drop | - | - | - |
|  |  | Change in ZPP 6 to 19 months | -0.02 (0.03) | 0.57 | drop | - | - | - |
|  |  | Child 6-month LAZ | -0.04 (0.03) | 0.19 | drop | - | - | - |
|  |  | Change in LAZ 6 to 18 months | -0.08 (0.02) | 0.0001 | retain | -0.028 (0.007) | 0.0000 | retain |
|  |  | Change in WLZ 6 to 18 months | 0.03 (0.03) | 0.21 | drop | - | - | - |
|  |  | Accelerometer Mean Vector Magnitude | -0.03 (0.03) | 0.30 | drop | - | - | - |
|  |  | Positive behavior during assessment | -0.05 (0.06) | 0.38 | drop | - | - | - |
|  |  | 18-Month variety of play materials | -0.03 (0.02) | 0.16 | drop | - | - | - |
|  |  | 18-Month activities with caregivers | -0.04 (0.02) | 0.12 | drop | - | - | - |
|  | Paternal Education | |  |  |  |  |  |  |
|  |  | Maternal Height | 0.10(0.03) | 0.0005 | retain | 0.001 (0.003) | 0.74 | drop |
|  |  | Child 6-month ZPP Concentration | 0.02 (0.03) | 0.45 | drop | - | - | - |
|  |  | Change in ZPP 6 to 19 months | -0.03 (0.03) | 0.33 | drop | - | - | - |
|  |  | Child 6-month LAZ | 0.08 (0.03) | 0.0035 | retain | 0.017 (0.006) | 0.01 | retain |
|  |  | Change in LAZ 6 to 18 months | 0.05 (0.02) | 0.02 | retain | 0.009 (0.006) | 0.13 | drop |
|  |  | Change in WLZ 6 to 18 months | -0.05 (0.03) | 0.04 | retain | 0.001 (0.002) | 0.81 | drop |
|  |  | Accelerometer Mean Vector Magnitude | -0.03 (0.03) | 0.31 | drop | - | - | - |
|  |  | Positive behavior during assessment | 0.14 (0.06) | 0.03 | retain | 0.017 (0.008) | 0.03 | retain |
|  |  | 18-Month variety of play materials | 0.15 (0.02) | <.0001 | retain | 0.009 (0.005) | 0.052 | drop |
|  |  | 18-Month activities with caregivers | 0.16 (0.02) | <.0001 | retain | 0.011 (0.005) | 0.047 | retain |
|  | Unimproved water source | |  |  |  |  |  |  |
|  |  | Maternal Height | -0.31 (0.11) | 0.00 | retain | -0.002 (0.004) | 0.52 | drop |
|  |  | Child 6-month ZPP Concentration | -0.02 (0.11) | 0.88 | drop | - | - | - |
|  |  | Change in ZPP 6 to 19 months | 0.06 (0.13) | 0.65 | drop | - | - | - |
|  |  | Child 6-month LAZ | -0.34 (0.11) | 0.00 | retain | -0.018 (0.008) | 0.02 | retain |
|  |  | Change in LAZ 6 to 18 months | -0.01 (0.08) | 0.87 | drop | - | - | - |
|  |  | Change in WLZ 6 to 18 months | 0.06 (0.1) | 0.56 | drop | - | - | - |
|  |  | Accelerometer Mean Vector Magnitude | -0.29 (0.12) | 0.02 | retain | -0.007 (0.004) | 0.10 | drop |
|  |  | Positive behavior during assessment | 11.5^1^ | 0.001 | retain | -0.029 (0.014) | 0.04 | retain |
|  |  | 18-Month variety of play materials | 0.09 (0.1) | 0.36 | drop | - | - | - |
|  |  | 18-Month activities with caregivers | -0.13 (0.1) | 0.17 | drop | - | - | - |
|  | Maternal Height | |  |  |  |  |  |  |
|  |  | Child 6-month ZPP Concentration | -0.07 (0.03) | 0.01 | retain | 0.011 (0.005) | 0.04 | retain |
|  |  | Change in ZPP 6 to 19 months | 0.08 (0.03) | 0.01 | retain | -0.007 (0.005) | 0.13 | drop |
|  |  | Child 6-month LAZ | 0.29 (0.03) | <.0001 | retain | 0.063 (0.012) | 0.000 | retain |
|  |  | Change in LAZ 6 to 18 months | 0.01 (0.02) | 0.57 | drop |  |  |  |
|  |  | Change in WLZ 6 to 18 months | 0.05 (0.03) | 0.04 | retain | 0.003 (0.003) | 0.25 | drop |
|  |  | Accelerometer Mean Vector Magnitude | 0.02 (0.03) | 0.41 | drop | - | - | - |
|  |  | Positive behavior during assessment | 0.06 (0.06) | 0.31 | drop | - | - | - |
|  |  | 18-Month variety of play materials | -0.01 (0.02) | 0.68 | drop |  |  |  |
|  |  | 18-Month activities with caregivers | 0.07 (0.02) | 0.00 | retain | 0.006 (0.004) | 0.12 | drop |
|  | Child 6-month ZPP Concentration | |  |  |  |  |  |  |
|  |  | Accelerometer Mean Vector Magnitude | 0 (0.03) | 0.91 | drop | - | - | - |
|  |  | Positive behavior during assessment | -0.02 (0.06) | 0.70 | drop | - | - | - |
|  |  | 18-Month variety of play materials | 0 (0.02) | 0.96 | drop | - | - | - |
|  |  | 18-Month activities with caregivers | -0.01 (0.02) | 0.67 | drop | - | - | - |
|  | Change in ZPP 6 to 18 months | |  |  |  |  |  |  |
|  |  | Accelerometer Mean Vector Magnitude | -0.12 (0.03) | 0.001 | retain | -0.018 (0.008) | 0.01 | retain |
|  |  | Positive behavior during assessment | 0.00 (0.00) | 0.10 | drop | - | - | - |
|  |  | 18-Month variety of play materials | -0.01 (0.03) | 0.70 | drop | - | - | - |
|  |  | 18-Month activities with caregivers | 0.01 (0.03) | 0.74 | drop | - | - | - |
|  | Child 6-month LAZ | |  |  |  |  |  |  |
|  |  | Accelerometer Mean Vector Magnitude | -0.02 (0.03) | 0.60 | drop | - | - | - |
|  |  | Positive behavior during assessment | 0.14 (0.06) | 0.02 | retain | 0.029 (0.009) | 0.001 | retain |
|  |  | 18-Month variety of play materials | 0.01 (0.02) | 0.76 | drop | - | - | - |
|  |  | 18-Month activities with caregivers | 0.03 (0.02) | 0.21 | drop | - | - | - |
|  | Change in LAZ 6 to 18 months | |  |  |  |  |  |  |
|  |  | Accelerometer Mean Vector Magnitude | 0.26 (0.04) | <.0001 | retain | 0.031 (0.01) | 0.00 | retain |
|  |  | Positive behavior during assessment | 0.16 (0.08) | 0.049 | retain | 0.014 (0.006) | 0.03 | retain |
|  |  | 18-Month variety of play materials | 0.04 (0.03) | 0.24 | drop | - | - | - |
|  |  | 18-Month activities with caregivers | 0.04 (0.03) | 0.28 | drop | - | - | - |
|  | Change in WLZ 6 to 18 months | |  |  |  |  |  |  |
|  |  | Accelerometer Mean Vector Magnitude | 0.10(0.03) | 0.002 | retain | 0.013 (0.006) | 0.02 | retain |
|  |  | Positive behavior during assessment | 0.09 (0.07) | 0.17 | drop | - | - | - |
|  |  | 18-Month variety of play materials | -0.01 (0.03) | 0.76 | drop | - | - | - |
|  |  | 18-Month activities with caregivers | 0.02 (0.03) | 0.42 | drop | - | - | - |

^1^Wald Chi-Square

TableS12. Pathway Selection Results for Cohort D: ZINC-Burkina Faso

| Dependent Variable | Independent Variable (IV) | Potential Mediator | Association between IV and Potential Mediator | | | Indirect effect in Multiple Mediation Model | | |
| --- | --- | --- | --- | --- | --- | --- | --- | --- |
|  |  |  | Estimate (SE) | p-value | Decision | Estimate (SE) | p-value | Decision |
| Language Z-Score | |  |  |  |  |  |  |  |
|  | Distance to Nearest Market | |  |  |  |  |  |  |
|  |  | Maternal Height | 0.03 (0.03) | 0.29 | drop | - | - | - |
|  |  | Child 9-month ZPP Concentration | -0.02 (0.03) | 0.44 | drop | - | - | - |
|  |  | Child 9-month LAZ | -0.01 (0.03) | 0.67 | drop | - | - | - |
|  |  | Change in LAZ 9 to 18 months | -0.01 (0.02) | 0.59 | drop | - | - | - |
|  |  | Child 9-month WLZ | 0 (0.03) | 0.93 | drop | - | - | - |
|  |  | Mean meal frequency in past 24 hours | 0.01 (0.03) | 0.87 | drop | - | - | - |
|  |  | Mean dietary diversity in past 7 days | -0.05 (0.03) | 0.11 | drop | - | - | - |
|  |  | 18-Month variety of play materials | 0.11 (0.03) | 0.0004 | retain | 0.034 (0.01) | 0.001 | retain |
|  |  | 18-Month activities with caregivers | -0.02 (0.03) | 0.60 | drop | - | - | - |
|  | Unimproved water source | |  |  |  |  |  |  |
|  |  | Maternal Height | 0.04 (0.07) | 0.53 | drop | - | - | - |
|  |  | Child 9-month ZPP Concentration | 0.05 (0.07) | 0.48 | drop | - | - | - |
|  |  | Child 9-month LAZ | -0.19 (0.07) | 0.01 | retain | -0.012 (0.005) | 0.02 | retain |
|  |  | Change in LAZ 9 to 18 months | -0.04 (0.04) | 0.30 | drop | - | - | - |
|  |  | Child 9-month WLZ | -0.07 (0.07) | 0.33 | drop | - | - | - |
|  |  | Mean meal frequency in past 24 hours | -0.21 (0.07) | 0.002 | retain | -0.012 (0.005) | 0.02 | retain |
|  |  | Mean dietary diversity in past 7 days | -0.13 (0.07) | 0.051 | drop | - | - | - |
|  |  | 18-Month variety of play materials | -0.15 (0.07) | 0.02 | retain | -0.012 (0.007) | 0.09 | drop |
|  |  | 18-Month activities with caregivers | -0.14 (0.07) | 0.03 | retain | -0.007 (0.004) | 0.07 | drop |
|  | Maternal Height | |  |  |  |  |  |  |
|  |  | Child 9-month ZPP Concentration | 0.00(0.03) | 0.93 | drop | - | - | - |
|  |  | Child 9-month LAZ | 0.34 (0.03) | <.0001 | retain | 0.053 (0.012) | 0.0000 | retain |
|  |  | Change in LAZ 9 to 18 months | -0.02 (0.02) | 0.30 | drop | - | - | - |
|  |  | Child 9-month WLZ | 0.06 (0.03) | 0.04 | retain | 0.007 (0.004) | 0.055 | drop |
|  |  | Mean meal frequency in past 24 hours | 0.03 (0.03) | 0.29 | drop | - | - | - |
|  |  | Mean dietary diversity in past 7 days | 0.07 (0.03) | 0.01 | retain | 0.01 (0.005) | 0.04 | retain |
|  |  | 18-Month variety of play materials | 0.05 (0.03) | 0.08 | drop | - | - | - |
|  |  | 18-Month activities with caregivers | 0.05 (0.03) | 0.08 | drop | - | - | - |
|  | Intervention Cohort | |  |  |  |  |  |  |
|  |  | Change in LAZ 9 to 18 months | 0.24 (0.04) | <.0001 | retain | 0.022 (0.006) | 0.00 | retain |
|  |  | Mean meal frequency in past 24 hours | 0.32 (0.06) | <.0001 | retain | 0.015 (0.006) | 0.01 | retain |
|  |  | Mean dietary diversity in past 7 days | 0.20(0.06) | 0.002 | retain | 0.005 (0.003) | 0.11 | drop |
|  |  | 18-Month variety of play materials | 0.21 (0.06) | 0.001 | retain | 0.02 (0.008) | 0.01 | retain |
|  |  | 18-Month activities with caregivers | -0.06 (0.06) | 0.34 | drop | - | - | - |
|  | Mean meal frequency in past 24 hours | |  |  |  |  |  |  |
|  |  | Change in LAZ 9 to 18 months | 0.02 (0.02) | 0.25 | drop | - | - | - |
|  | Mean dietary diversity in past 7 days | |  |  |  |  |  |  |
|  |  | Change in LAZ 9 to 18 months | 0.02 (0.02) | 0.31 | drop | - | - | - |
|  | Child 9-month ZPP Concentration | |  |  |  |  |  |  |
|  |  | 18-Month variety of play materials | -0.05 (0.03) | 0.07 | drop | - | - | - |
|  |  | 18-Month activities with caregivers | -0.03 (0.03) | 0.26 | drop | - | - | - |
|  | Child 9-month LAZ | |  |  |  |  |  |  |
|  |  | 18-Month variety of play materials | 0.15 (0.03) | <.0001 | retain | 0.043 (0.01) | 0.00 | retain |
|  |  | 18-Month activities with caregivers | 0.05 (0.03) | 0.055 | drop | - | - | - |
|  | Change in LAZ 9 to 18 months | |  |  |  |  |  |  |
|  |  | 18-Month variety of play materials | 0.27 (0.05) | <.0001 | retain | 0.037 (0.009) | 0.00 | retain |
|  |  | 18-Month activities with caregivers | 0.15 (0.05) | 0.004 | retain | 0.013 (0.006) | 0.03 | retain |
|  | Child 9-month WLZ | |  |  |  |  |  |  |
|  |  | 18-Month variety of play materials | 0.14 (0.03) | <.0001 | retain | 0.036 (0.011) | 0.00 | retain |
|  |  | 18-Month activities with caregivers | 0.07 (0.03) | 0.04 | retain | 0.01 (0.005) | 0.07 | drop |
| Motor Z-Score |  |  |  |  |  |  |  |  |
|  | Asset Index |  |  |  |  |  |  |  |
|  |  | Maternal Height | 0.00(0.03) | 0.94 | drop | - | - | - |
|  |  | Maternal Body Mass Index | 0.08 (0.03) | 0.005 | retain | 0.003 (0.003) | 0.26 | drop |
|  |  | Child 9-month ZPP Concentration | -0.03 (0.03) | 0.26 | drop | - | - | - |
|  |  | Child 9-month Hb Concentration (g/L) | 0.08 (0.03) | 0.004 | retain | 0.001 (0.002) | 0.60 | drop |
|  |  | Change in Hb 9 to 18 months | -0.03 (0.03) | 0.35 | drop | - | - | - |
|  |  | Child 9-month LAZ | 0.07 (0.03) | 0.03 | retain | 0.02 (0.008) | 0.01 | retain |
|  |  | Change in LAZ 9 to 18 months | 0.03 (0.02) | 0.09 | drop | - | - | - |
|  |  | Child 9-month WLZ | 0.07 (0.03) | 0.03 | retain | 0.008 (0.004) | 0.045 | retain |
|  |  | Mean dietary diversity in past 7 days | 0.05 (0.03) | 0.11 | drop | - | - | - |
|  |  | 18-Month variety of play materials | 0.18 (0.03) | <.0001 | retain | 0.058 (0.01) | 0.0000 | retain |
|  |  | 18-Month activities with caregivers | 0.04 (0.03) | 0.21 | drop | - | - | - |
|  | Maternal Height | |  |  |  |  |  |  |
|  |  | Child 9-month ZPP Concentration | 0.00(0.03) | 0.93 | drop | - | - | - |
|  |  | Child 9-month Hb Concentration (g/L) | -0.02 (0.03) | 0.50 | drop | - | - | - |
|  |  | Change in Hb 9 to 18 months | 0.00(0.03) | 0.91 | drop | - | - | - |
|  |  | Child 9-month LAZ | 0.34 (0.03) | <.0001 | retain | 0.102 (0.014) | 0.0000 | retain |
|  |  | Change in LAZ 9 to 18 months | -0.02 (0.02) | 0.30 | drop | - | - | - |
|  |  | Child 9-month WLZ | 0.06 (0.03) | 0.04 | retain | 0.012 (0.005) | 0.01 | retain |
|  |  | Mean dietary diversity in past 7 days | 0.07 (0.03) | 0.01 | retain | 0.008 (0.004) | 0.04 | retain |
|  |  | 18-Month variety of play materials | 0.05 (0.03) | 0.08 | drop | - | - | - |
|  |  | 18-Month activities with caregivers | 0.05 (0.03) | 0.08 | drop | - | - | - |
|  | Maternal Body Mass Index | |  |  |  |  |  |  |
|  |  | Child 9-month ZPP Concentration | -0.01 (0.03) | 0.76 | drop | - | - | - |
|  |  | Child 9-month Hb Concentration (g/L) | 0.06 (0.03) | 0.03 | retain | 0.008 (0.005) | 0.09 | drop |
|  |  | Change in Hb 9 to 18 months | -0.06 (0.03) | 0.03 | retain | -0.01 (0.006) | 0.08 | drop |
|  |  | Child 9-month LAZ | 0.15 (0.03) | <.0001 | retain | 0.036 (0.008) | 0.0000 | retain |
|  |  | Change in LAZ 9 to 18 months | 0.00(0.02) | 0.83 | drop | - | - | - |
|  |  | Child 9-month WLZ | 0.2(0.03) | <.0001 | retain | 0.024 (0.007) | 0.0004 | retain |
|  |  | Mean dietary diversity in past 7 days | -0.01 (0.03) | 0.67 | drop | - | - | - |
|  |  | 18-Month variety of play materials | 0.08 (0.03) | 0.01 | retain | 0.025 (0.01) | 0.01 | retain |
|  |  | 18-Month activities with caregivers | 0.02 (0.03) | 0.46 | drop | - | - | - |
|  | Intervention Cohort | |  |  |  |  |  |  |
|  |  | Change in Hb 9 to 18 months | 0.42 (0.06) | <.0001 | retain | 0.03 (0.008) | 0.0002 | retain |
|  |  | Change in LAZ 9 to 18 months | 0.24 (0.04) | <.0001 | retain | 0.045 (0.008) | 0.0000 | retain |
|  |  | Mean dietary diversity in past 7 days | 0.2(0.06) | 0.002 | retain | 0.005 (0.003) | 0.08 | drop |
|  |  | 18-Month variety of play materials | 0.21 (0.06) | 0.001 | retain | 0.031 (0.01) | 0.003 | retain |
|  |  | 18-Month activities with caregivers | -0.06 (0.06) | 0.34 | drop | - | - | - |
|  | Mean dietary diversity in past 7 days | |  |  |  |  |  |  |
|  |  | Change in Hb 9 to 18 months | 0.05 (0.03) | 0.12 | drop | - | - | - |
|  |  | Change in LAZ 9 to 18 months | 0.02 (0.02) | 0.31 | drop | - | - | - |
|  | Child 9-month ZPP Concentration | |  |  |  |  |  |  |
|  |  | 18-Month variety of play materials | -0.05 (0.03) | 0.07 | drop | - | - | - |
|  |  | 18-Month activities with caregivers | -0.03 (0.03) | 0.26 | drop | - | - | - |
|  | Child 9-month Hb Concentration (g/L) | |  |  |  |  |  |  |
|  |  | 18-Month variety of play materials | 0.19 (0.04) | <.0001 | retain | 0.074 (0.016) | <0.0001 | retain |
|  |  | 18-Month activities with caregivers | 0.12 (0.04) | 0.002 | retain | 0.008 (0.004) | 0.07 | drop |
|  | Change in Hb 9 to 18 months | |  |  |  |  |  |  |
|  |  | 18-Month variety of play materials | 0.09 (0.04) | 0.03 | retain | 0.034 (0.016) | 0.03 | retain |
|  |  | 18-Month activities with caregivers | 0.06 (0.04) | 0.11 | drop | - | - | - |
|  | Child 9-month LAZ | |  |  |  |  |  |  |
|  |  | 18-Month variety of play materials | 0.15 (0.03) | <.0001 | retain | 0.057 (0.011) | <0.0001 | retain |
|  |  | 18-Month activities with caregivers | 0.05 (0.03) | 0.055 | drop | - | - | - |
|  | Change in LAZ 9 to 18 months | |  |  |  |  |  |  |
|  |  | 18-Month variety of play materials | 0.27 (0.05) | <.0001 | retain | 0.054 (0.011) | <0.01 | retain |
|  |  | 18-Month activities with caregivers | 0.15 (0.05) | 0.004 | retain | 0.005 (0.003) | 0.09 | drop |
|  | Child 9-month WLZ | |  |  |  |  |  |  |
|  |  | 18-Month variety of play materials | 0.14 (0.03) | <.0001 | retain | 0.053 (0.013) | <0.0001 | retain |
|  |  | 18-Month activities with caregivers | 0.07 (0.03) | 0.04 | retain | 0.004 (0.003) | 0.15 | drop |

Table S13. Coefficients for Direct and Indirect Effects on Language Development in the Final Models

|  |  |  | **Cohort A**:  DYAD-Ghana | | **Cohort B**:  DYAD-Malawi | | **Cohort C**:  DOSE-Malawi | | **Cohort D**:  ZINC-Burkina Faso | |
| --- | --- | --- | --- | --- | --- | --- | --- | --- | --- | --- |
|  | Dependent Variable/  Mediator | Independent Variable | Estimate^a^ (SE) | % of effect direct (top) or indirect (bottom)^b^ | Estimate^a^ (SE) | % of effect direct (top) or indirect (bottom)^b^ | Estimate^a^ (SE) | % of effect direct (top) or indirect (bottom)^b^ | Estimate^a^ (SE) | % of effect direct (top) or indirect (bottom)^b^ |
| Direct Effects | | |  |  |  |  |  |  |  |  |
|  | Language Z-Score | |  |  |  |  |  |  |  |  |
|  |  | Asset Index | -0.01 (0.03) | 15% | - | - | - | - | - | - |
|  |  | Distance to Nearest Market | - | - | - | - | - | - | 0.07* (0.03) | 56% |
|  |  | Paternal Education | - | - | 0.03 (0.04) | 78% | 0.01 (0.03) | 18% | - | - |
|  |  | Unimproved water source | - | - | - | - | - | - | -0.11† (0.06) | 67% |
|  |  | Maternal Age | 0.09** (0.03) | 78% | - | - | - | - | - | - |
|  |  | Maternal Height | - | - | 0.07† (0.04) | 100% | - | - | -0.01 (0.03) | 0% |
|  |  | Maternal Cognitive Z-Score | - | - | 0.06 (0.04) | 80% | - | - | - | - |
|  |  | Firstborn | -0.13† (0.07) | 89% | -0.20* (0.09) | 100% | - | - | - | - |
|  |  | LAZ at Birth/6/9 mo | 0.10** (0.03) | 100% | - | - | 0.14*** (0.03) | 100% | 0.14*** (0.03) | 79% |
|  |  | Change in LAZ Birth/6/9 to 18 mo | 0.08* (0.04) | 100% | - | - | 0.11** (0.04) | 100% | 0.18*** (0.05) | 72% |
|  |  | BMIZ/WLZ at Birth/6/9 mo | - | - | - | - | - | - | 0.05† (0.03) | 76% |
|  |  | Child 6-month Hb Concentration | 0.09* (0.04) | 100% | 0.10*† (0.05) | 100% | - | - | - | - |
|  |  | Change in Hb 6 to 18 months | 0.09* (0.04) | 100% | 0.12* (0.05) | 100% | - | - | - | - |
|  |  | Child 9-month ZPP Concentration | - | - | - | - | - | - | -0.07* (0.03) | 100% |
|  |  | Mean 24-hr feeding frequency | - | - | - | - | - | - | 0.07* (0.03) | 100% |
|  |  | Mean 7-day dietary diversity | 0.08* (0.03) | 100% | - | - | 0.11*** (0.03) | 100% | 0.07* (0.03) | 100% |
|  |  | 6-month HOME Total Score | - | - | 0.10* (0.04) | 100% | - | - | - | - |
|  |  | 18-Month variety of play materials | 0.17*** (0.03) | 100% | 0.22*** (0.04) | 100% | 0.17*** (0.03) | 100% | 0.19*** (0.03) | 100% |
|  |  | 18-Month activities with caregivers | 0.08** (0.03) | 100% | - | - | 0.06† (0.03) | 100% | 0.13*** (0.03) | 100% |
|  |  | Exposed to > 1 language | 0.32*** (0.08) | 100% | 0.18* (0.09) | 100% | 0.24*** (0.06) | 100% | 0.13 (0.11) | 100% |
|  |  | iLiNS Intervention | - | - | - | - | - | - | 0.23*** (0.06) | 64% |
| Indirect Effects | |  |  |  |  |  |  |  |  |  |
|  | Maternal Age | |  |  |  |  |  |  |  |  |
|  |  | Asset Index | 0.16*** (0.03) | 20% | - | - | - | - | - | - |
|  | LAZ at Birth/6/9 mo | |  |  |  |  |  |  |  |  |
|  |  | Asset Index | 0.09** (0.03) | 11% | - | - | - | - | - | - |
|  |  | Paternal Education | - | - | - | - | 0.08** (0.03) | 15% | - | - |
|  |  | Unimproved water source | - | - | - | - | - | - | -0.20** (0.07) | 16% |
|  |  | Maternal Age | 0.09** (0.03) | 5% | - | - | - | - | - | - |
|  |  | Maternal Height | - | - | - | - | - | - | 0.34*** (0.03) | 86% |
|  | Change in LAZ Birth/6/9 to 18 mo | |  |  |  |  |  |  |  |  |
|  |  | iLiNS Intervention | - | - | - | - | - | - | 0.24*** (0.04) | 13% |
|  | Mean 24-hr feeding frequency | |  |  |  |  |  |  |  |  |
|  |  | Unimproved water source | - | - | - | - | - | - | -0.19** (0.07) | 17% |
|  |  | iLiNS Intervention | - | - | - | - | - | - | 0.31*** (0.06) | 11% |
|  | Mean 7-day dietary diversity | |  |  |  |  |  |  |  |  |
|  |  | Asset Index | 0.17*** (0.03) | 17% | - | - | - | - | - | - |
|  |  | Paternal Education | - | - | - | - | 0.15*** (0.03) | 21% | - | - |
|  |  | Maternal Age | 0.08** (0.03) | 8% | - | - | - | - | - | - |
|  |  | Maternal Height | - | - | - | - | - | - | 0.07* (0.03) | 14% |
|  | 6-month HOME Total Score | |  |  |  |  |  |  |  |  |
|  |  | Maternal Cognitive Z-Score | - | - | 0.25*** (0.04) | 20% | - | - | - | - |
|  | 18-Month variety of play materials | |  |  |  |  |  |  |  |  |
|  |  | Asset Index | 0.18*** (0.03) | 27% | - | - | - | - | - | - |
|  |  | Distance to Nearest Market | - | - | - | - | - | - | 0.11*** (0.03) | 44% |
|  |  | Paternal Education | - | - | 0.10** (0.04) | 22% | 0.16*** (0.02) | 29% | - | - |
|  |  | Maternal Age | 0.07* (0.03) | 10% | - | - | - | - | - | - |
|  |  | LAZ at Birth/6/9 mo | - | - | - | - | - | - | 0.12*** (0.03) | 21% |
|  |  | Change in LAZ Birth/6/9 to 18 mo | - | - | - | - | - | - | 0.24*** (0.05) | 21% |
|  |  | BMIZ/WLZ at Birth/6/9 mo | - | - | - | - | - | - | 0.08* (0.03) | 24% |
|  |  | iLiNS Intervention | - | - | - | - | - | - | 0.13* (0.06) | 12% |
|  | 18-Month activities with caregivers | |  |  |  |  |  |  |  |  |
|  |  | Asset Index | 0.18*** (0.04) | 10% | - | - | - | - | - | - |
|  |  | Paternal Education | - | - | - | - | 0.16*** (0.02) | 17% | - | - |
|  |  | Child Firstborn | 0.22** (0.07) | 11% | - | - | - | - | - | - |
|  |  | Change in LAZ Birth/6/9 to 18 mo | - | - | - | - | - | - | 0.16** (0.05) | 7% |
|  | *R^2^* |  | 0.18 |  | 0.14 |  | 0.14 |  | 0.25 |  |

^a^Estimates are standardized such that they represent the change in units of SD of the dependent variable/mediator for each 1 SD change in the independent variable.

^b^In the top half of the table, under the heading “Direct Effects” the percentage represents the proportion of the effect of the independent variable that is directly associated with the dependent variable. In the bottom half of the table, under the heading “Indirect Effects” the percentage represents the proportion of the effect of the independent variable on the dependent variable that is mediated by the mediator.

†*p*< 0.1 both uncorrected and after correcting for multiple comparisons using the Benjamini Hochberg correction

**p*< 0.05 both uncorrected and after correcting for multiple comparisons using the Benjamini Hochberg correction

***p*< 0.01uncorrected and *p*< 0.05 after correcting for multiple comparisons using the Benjamini Hochberg correction

****p*< 0.001 uncorrected and*p*< 0.05 after correcting for multiple comparisons using the Benjamini Hochberg correction

*†*p*< 0.05 uncorrected and 0.05 <*p*< 0.1 after correcting for multiple comparisons using the Benjamini Hochberg correction

Table S14. Coefficients for Direct and Indirect Effects on Motor Development in the Final Models

|  |  |  | **Cohort A**:  DYAD-Ghana | | **Cohort B**:  DYAD-Malawi | | **Cohort C**:  DOSE-Malawi | | **Cohort D**:  ZINC-Burkina Faso | |
| --- | --- | --- | --- | --- | --- | --- | --- | --- | --- | --- |
|  | Dependent Variable/  Mediator | Independent Variable | Estimate^a^ (SE) | % of effect direct (top) or indirect (bottom)^b^ | Estimate^a^ (SE) | % of effect direct (top) or indirect (bottom)^b^ | Estimate^a^ (SE) | % of effect direct (top) or indirect (bottom)^b^ | Estimate^a^ (SE) | % of effect direct (top) or indirect (bottom)^b^ |
| Direct Effects | |  |  |  |  |  |  |  |  |  |
|  | Motor Z-Score | |  |  |  |  |  |  |  |  |
|  |  | Asset Index | - | - | - | - | - | - | 0.04† (0.02) | 42% |
|  |  | Distance to Nearest Market | - | - | - | - | -0.08** (0.03) | 79% | - | - |
|  |  | Paternal Education | - | - | - | - | 0.07* (0.03) | 60% | - | - |
|  |  | Unimproved water source | - | - | - | - | -0.06 (0.10) | 44% | - | - |
|  |  | Maternal Height | - | - | - | - | 0.00 (0.03) | 14% | -0.04 (0.03) | 0% |
|  |  | Maternal Body Mass Index | - | - | - | - | - | - | 0.03 (0.02) | 30% |
|  |  | Maternal Baseline CRP | 0.07* (0.03) | 100% | - | - | - | - | - | - |
|  |  | Maternal Basal Cortisol at 36 wk gestation | - | - | -0.11* (0.04) | 100% | - | - | - | - |
|  |  | Maternal Functional Health Literacy | - | - | 0.09* (0.04) | 100% | - | - | - | - |
|  |  | LAZ at Birth/6/9 mo | 0.06† (0.04) | 100% | 0.12** (0.04) | 92% | 0.16*** (0.03) | 88% | 0.27*** (0.03) | 85% |
|  |  | Change in LAZ Birth/6/9 to 18 mo | 0.12** (0.04) | 100% | 0.18*** (0.04) | 100% | 0.25*** (0.04) | 82% | 0.36*** (0.04) | 82% |
|  |  | BMIZ/WLZ at Birth/6/9 mo | 0.12** (0.04) | 100% | - | - | 0.05† (0.03) | 100% | 0.11*** (0.03) | 82% |
|  |  | Change in BMIZ/WLZ Birth/6/9 to 18 mo | 0.07* (0.03) | 100% | - | - | 0.08* (0.03) | 86% | - | - |
|  |  | Child 9-month Hb Concentration | - | - | - | - | - | - | 0.06† (0.03) | 70% |
|  |  | Change in Hb 9 to 18 months | - | - | - | - | - | - | 0.13*** (0.03) | 86% |
|  |  | Child 6 or 9-month ZPP Concentration | - | - | - | - | -0.10*** (0.03) | 100% | -0.09** (0.03) | 100% |
|  |  | Change in ZPP 6 to 18 months | - | - | - | - | -0.05 (0.03) | 81% | - | - |
|  |  | Child 18-month AGP | - | - | -0.07† (0.04) | 67% | - | - | - | - |
|  |  | Child 18-month Basal Cortisol | - | - | -0.12** (0.04) | 100% | - | - | - | - |
|  |  | Child 18-month Activity | - | - | - | - | 0.06*† (0.03) | 100% | - | - |
|  |  | Positive behavior during assessment | 0.67*** (0.06) | 100% | 0.57*** (0.07) | 100% | 0.50*** (0.06) | 100% | - | - |
|  |  | Mean 7-day dietary diversity score | - | - | - | - | - | - | 0.05† (0.02) | 100% |
|  |  | 18-Month variety of play materials | - | - | 0.12** (0.04) | 100% | 0.06*† (0.03) | 100% | 0.29*** (0.03) | 100% |
|  |  | 18-Month activities with caregivers | 0.13*** (0.03) | 100% | - | - | 0.06*† (0.03) | 100% | 0.06*† (0.03) | 100% |
|  |  | iLiNS Intervention | - | - | - | - | - | - | 0.23*** (0.06) | 45% |
| Indirect Effects | |  |  |  |  |  |  |  |  |  |
|  | LAZ at Birth/6/9 mo | |  |  |  |  |  |  |  |  |
|  |  | Asset Index | - | - | - | - | - | - | 0.06*† (0.03) | 13% |
|  |  | Paternal Education | - | - | - | - | 0.05*† (0.03) | 17% | - | - |
|  |  | Unimproved water source | - | - | - | - | -0.23* (0.10) | 22% | - | - |
|  |  | Maternal Height | - | - | - | - | 0.28*** (0.03) | 78% | 0.34*** (0.03) | 84% |
|  |  | Maternal Body Mass Index | - | - | - | - | - | - | 0.12*** (0.03) | 29% |
|  | Change in LAZ Birth/6/9 to 18 mo | |  |  |  |  |  |  |  |  |
|  |  | Distance to Nearest Market | - | - | - | - | -0.08*** (0.02) | 21% | - | - |
|  |  | iLiNS Intervention | - | - | - | - | - | - | 0.24*** (0.04) | 23% |
|  | BMIZ/WLZ at Birth/6/9 mo | |  |  |  |  |  |  |  |  |
|  |  | Asset Index | - | - | - | - | - | - | 0.05† (0.03) | 5% |
|  |  | Maternal Height | - | - | - | - | - | - | 0.06† (0.03) | 10% |
|  |  | Maternal Body Mass Index | - | - | - | - | - | - | 0.19*** (0.03) | 21% |
|  | Child 6 or 9-month ZPP Concentration | |  |  |  |  |  |  |  |  |
|  |  | Maternal Height | - | - | - | - | -0.07** (0.03) | 8% | - | - |
|  | Change in Hb 9 to 18 months | |  |  |  |  |  |  |  |  |
|  |  | iLiNS Intervention | - | - | - | - | - | - | 0.42*** (0.06) | 16% |
|  | Child 18-month Activity | |  |  |  |  |  |  |  |  |
|  |  | Change in LAZ Birth/6/9 to 18 mo | - | - | - | - | 0.28*** (0.04) | 5% | - | - |
|  |  | Change in BMIZ/WLZ Birth/6/9 to 18 mo | - | - | - | - | 0.12** (0.04) | 14% | - | - |
|  |  | Change in ZPP 6 to 18 months | - | - | - | - | -0.12** (0.04) | 19% | - | - |
|  | Positive behavior during assessment | |  |  |  |  |  |  |  |  |
|  |  | Paternal Education | - | - | - | - | 0.02 (0.01) | 12% | - | - |
|  |  | Unimproved water source | - | - | - | - | -0.12* (0.05) | 34% | - | - |
|  |  | LAZ at Birth/6/9 mo | - | - | - | - | 0.03** (0.01) | 12% | - | - |
|  |  | Change in LAZ Birth/6/9 to 18 mo | - | - | - | - | 0.04* (0.02) | 12% | - | - |
|  |  | Child 18-month AGP | - | - | -0.07** (0.02) | 33% | - | - | - | - |
|  | Mean 7-day dietary diversity score | |  |  |  |  |  |  |  |  |
|  |  | Maternal Height | - | - | - | - | - | - | 0.07* (0.03) | 6% |
|  | 18-Month variety of play materials | |  |  |  |  |  |  |  |  |
|  |  | Asset Index | - | - | - | - | - | - | 0.15*** (0.03) | 39% |
|  |  | Maternal Body Mass Index | - | - | - | - | - | - | 0.03 (0.03) | 20% |
|  |  | LAZ at Birth/6/9 mo | - | - | 0.12** (0.04) | 8% | - | - | 0.10* (0.03) | 15% |
|  |  | Change in LAZ Birth/6/9 to 18 mo | - | - | - | - | - | - | 0.22*** (0.05) | 18% |
|  |  | BMIZ/WLZ at Birth/6/9 mo | - | - | - | - | - | - | 0.06† (0.04) | 18% |
|  |  | Child 9-month Hb Concentration | - | - | - | - | - | - | 0.13** (0.04) | 30% |
|  |  | Change in Hb 9 to 18 months | - | - | - | - | - | - | 0.04 (0.04) | 14% |
|  |  | iLiNS Intervention | - | - | - | - | - | - | 0.07 (0.06) | 16% |
|  | 18-Month activities with caregivers | |  |  |  |  |  |  |  |  |
|  |  | Paternal Education | - | - | - | - | 0.16*** (0.02) | 10% | - | - |
|  | *R*^2^ |  | 0.21 |  | 0.36 |  | 0.31 |  | 0.36 |  |

^a^Estimates are standardized such that they represent the change in units of SD of the dependent variable/mediator for each 1 SD change in the independent variable.

^b^In the top half of the table, under the heading “Direct Effects” the percentage represents the proportion of the effect of the independent variable that is directly associated with the dependent variable. In the bottom half of the table, under the heading “Indirect Effects” the percentage represents the proportion of the effect of the independent variable on the dependent variable that is mediated by the mediator.

†*p*< 0.1 both uncorrected and after correcting for multiple comparisons using the Benjamini Hochberg correction

**p*< 0.05 both uncorrected and after correcting for multiple comparisons using the Benjamini Hochberg correction

***p*< 0.01 uncorrected and *p*< 0.05 after correcting for multiple comparisons using the Benjamini Hochberg correction

****p*< 0.001 uncorrected and *p*< 0.05 after correcting for multiple comparisons using the Benjamini Hochberg correction

*†*p*< 0.05 uncorrected and 0.05 <*p*< 0.1 after correcting for multiple comparisons using the Benjamini Hochberg correction

Figure S1. Percent of Socio-Economic Disparities Mediated by Each Category of Factors

References

Boivin, M. J., Sikorskii, A., Familiar-Lopez, I., Ruisenor-Escudero, H., Muhindo, M., Kapisi, J., et al. (2016). Malaria illness mediated by anaemia lessens cognitive development in younger Ugandan children. *Malar J, 15*(1), 210.

Caldwell, B. M., & Bradley, R. H. (2003). *Home Observation for Measurement of the Environment: Administration Manual*. Tempe, AZ: Family & Human Dynamics Research Institute, Arizona State University.

Coates, J., Swindale, A., & Bilinsky, P. (2007). *Household Food Insecurity Access Scale (HFIAS) for Measurement of Household Food Access: Indicator Guide (v. 3)*. Washington, D.C: Food and Nutrition Technical Assistance Project, Academy for Educational Development.

Cohen, S., Kamarck, T., & Mermelstein, R. (1983). A global measure of perceived stress. *J Health Soc Behav, 24*(4), 385-396.

Cox, J. L., Holden, J. M., & Sagovsky, R. (1987). Detection of postnatal depression: Development of the 10-item Edinburgh Postnatal Depression Scale. *British Journal of Psychiatry, 150*, 782-786.

Felt, B. T., Peirano, P., Algarin, C., Chamorro, R., Sir, T., Kaciroti, N., et al. (2012). Long-term neuroendocrine effects of iron-deficiency anemia in infancy. *Pediatr Res, 71*(6), 707-712.

Fernald, L. C., & Grantham-McGregor, S. M. (1998). Stress response in school-age children who have been growth retarded since early childhood. *The American journal of clinical nutrition, 68*(3), 691-698.

Fernald, L. C., Kariger, P., Hidrobo, M., & Gertler, P. J. (2012). Socioeconomic gradients in child development in very young children: evidence from India, Indonesia, Peru, and Senegal. *Proceedings of the National Academy of Sciences of the United States of America, 109 Suppl 2*, 17273-17280.

Frongillo, E. A., Nguyen, P. H., Saha, K. K., Sanghvi, T., Afsana, K., Haque, R., et al. (2016). Large-Scale Behavior-Change Initiative for Infant and Young Child Feeding Advanced Language and Motor Development in a Cluster-Randomized Program Evaluation in Bangladesh. *J Nutr*.

Hamadani, J. D., Tofail, F., Hilaly, A., Huda, S. N., Engle, P., & Grantham-McGregor, S. M. (2010). Use of family care indicators and their relationship with child development in Bangladesh. *J Health Popul Nutr, 28*(1), 23-33.

Hamadani, J. D., Tofail, F., Huda, S. N., Alam, D. S., Ridout, D. A., Attanasio, O., et al. (2014). Cognitive deficit and poverty in the first 5 years of childhood in Bangladesh. *Pediatrics, 134*(4), e1001-1008.

Jukes, M. C., & Grigorenko, E. L. (2010). Assessment of cognitive abilities in multiethnic countries: The case of the Wolof and Mandinka in the Gambia. *British Journal of Educational Psychology, 80*, 77-97.

Kariger, P., Frongillo, E. A., Engle, P., Britto, P. M., Sywulka, S. M., & Menon, P. (2012). Indicators of family care for development for use in multicountry surveys. *J Health Popul Nutr, 30*(4), 472-486.

Kitsao-Wekulo, P., Holding, P., Taylor, H. G., Abubakar, A., Kvalsvig, J., & Connolly, K. (2013). Nutrition as an important mediator of the impact of background variables on outcome in middle childhood. *Frontiers in human neuroscience, 7*, 713.

Knauer, H. A., Kagawa, R. M. C., García-Guerra, A., Schnaas, L., Neufeld, L. M., & Fernald, L. C. H. (2016). Pathways to improved development for children living in poverty: A randomized effectiveness trial in rural Mexico. *International Journal of Behavioral Development*.

Lopez Boo, F. (2016). Socio-economic status and early childhood cognitive skills: A mediation analysis using the Young Lives panel. *International Journal of Behavioral Development*.

McCoy, D. C., Zuilkowski, S. S., & Fink, G. (2015). Poverty, physical stature, and cognitive skills: Mechanisms underlying children's school enrollment in Zambia. *Dev Psychol, 51*(5), 600-614.

Murray, L., De Pascalis, L., Tomlinson, M., Vally, Z., Dadomo, H., MacLachlan, B., et al. (2016). Randomized controlled trial of a book-sharing intervention in a deprived South African community: effects on carer-infant interactions, and their relation to infant cognitive and socioemotional outcome. *J Child Psychol Psychiatry*.

Obradovic, J., Yousafzai, A. K., Finch, J. E., & Rasheed, M. A. (2016). Maternal Scaffolding and Home Stimulation: Key Mediators of Early Intervention Effects on Children's Cognitive Development. *Dev Psychol*.

Prado, E. L., Abbeddou, S., Yakes Jimenez, E., Some, J. W., Dewey, K. G., Brown, K. H., et al. (2016). Effects of an intervention on infant growth and development: evidence for different mechanisms at work. *Matern Child Nutr*.

Prado, E. L., Abbeddou, S., Yakes Jimenez, E., Some, J. W., Ouedraogo, Z. P., Vosti, S. A., et al. (2016). Lipid-Based Nutrient Supplements Plus Malaria and Diarrhea Treatment Increase Infant Development Scores in a Cluster-Randomized Trial in Burkina Faso. *J Nutr*.

Prado, E. L., Adu-Afarwuah, S., Lartey, A., Ocansey, M., Ashorn, P., Vosti, S. A., et al. (2016). Effects of pre- and post-natal lipid-based nutrient supplements on infant development in a randomized trial in Ghana. *Early Human Development, 99*, 43-51.

Prado, E. L., Maleta, K., Ashorn, P., Ashorn, U., Vosti, S. A., Sadalaki, J., et al. (2016). Effects of maternal and child lipid-based nutrient supplements on infant development: a randomized trial in Malawi. *Am J Clin Nutr, 103*(3), 784-793.

Prado, E. L., Phuka, J., Maleta, K., Ashorn, P., Ashorn, U., Vosti, S. A., et al. (2016). Provision of Lipid-Based Nutrient Supplements from Age 6 to 18 Months Does Not Affect Infant Development Scores in a Randomized Trial in Malawi. *Matern Child Health J, 20*(10), 2199-2208.

Pulakka, A., Cheung, Y. B., Ashorn, U., Penpraze, V., Maleta, K., Phuka, J. C., et al. (2013). Feasibility and validity of the ActiGraph GT3X accelerometer in measuring physical activity of Malawian toddlers. [Research Support, Non-U.S. Gov't]. *Acta paediatrica, 102*(12), 1192-1198.

Rubio-Codina, M., Attanasio, O., & Grantham-McGregor, S. (2016). Mediating pathways in the socio-economic gradient of child development: Evidence from children 6–42 months in Bogota. *International Journal of Behavioral Development*.

Santibañez, L., & Fagioli, L. (2016). Nothing succeeds like success? Equity, student outcomes, and opportunity to learn in high- and middle-income countries. *International Journal of Behavioral Development*.

Singla, D. R., Kumbakumba, E., & Aboud, F. E. (2015). Effects of a parenting intervention to address both maternal psychological wellbeing and child development and growth in rural Uganda: a community-based, cluster randomised trial. *Lancet Glob Health, 3*(8), e458-469.

Stewart, C. P., Oaks, B. M., Laugero, K. D., Ashorn, U., Harjunmaa, U., Kumwenda, C., et al. (2015). Maternal cortisol and stress are associated with birth outcomes, but are not affected by lipid-based nutrient supplements during pregnancy: an analysis of data from a randomized controlled trial in rural Malawi. *BMC Pregnancy Childbirth, 15*(1), 346.

Stewart, R. C., Ashorn, P., Umar, E., Dewey, K. G., Ashorn, U., Creed, F., et al. (2016). The impact of maternal diet fortification with lipid-based nutrient supplements on postpartum depression in rural Malawi: a randomised-controlled trial. *Maternal & Child Nutrition*, n/a-n/a.

Stewart, R. C., Kauye, F., Umar, E., Vokhiwa, M., Bunn, J., Fitzgerald, M., et al. (2009). Validation of a Chichewa version of the self-reporting questionnaire (SRQ) as a brief screening measure for maternal depressive disorder in Malawi, Africa. *Journal of Affective Disorders, 112*(1-3), 126-134.

Surkan, P. J., Charles, M. K., Katz, J., Siegel, E. H., Khatry, S. K., LeClerq, S. C., et al. (2015). The role of zinc and iron-folic acid supplementation on early child temperament and eating behaviors in rural Nepal: a randomized controlled trial. *PLoS One, 10*(3), e0114266.

Tran, T. D., Luchters, S., & Fisher, J. (2016). Early childhood development: impact of national human development, family poverty, parenting practices and access to early childhood education. *Child Care Health Dev*.

Tran, T. D., Tran, T., Simpson, J. A., Tran, H. T., Nguyen, T. T., Hanieh, S., et al. (2014). Infant motor development in rural Vietnam and intrauterine exposures to anaemia, iron deficiency and common mental disorders: a prospective community-based study. *BMC Pregnancy Childbirth, 14*, 8.

Trost, S. G., Fees, B. S., Haar, S. J., Murray, A. D., & Crowe, L. K. (2012). Identification and validity of accelerometer cut-points for toddlers. *Obesity (Silver Spring), 20*(11), 2317-2319.

Vyas, S., & Kumaranayake, L. (2006). Constructing socio-economic status indices: how to use principal components analysis. *Health Policy and Planning, 21*(6), 459-468.

WHO. (1994). *A user's guide to the Self-Reporting Questionnaire (SRQ)*. Geneva: World Health Organization.

WHO Multicentre Growth Reference Study Group. (2006). *WHO Child Growth Standards: Length/height-for-age, weight-for-age, weight-for-length, weight-for-height and body mass index-for-age: Methods and development*. Geneva: World Health Organization.

Woldehanna, T. (2016). Inequality, preschool education and cognitive development in Ethiopia: Implication for public investment in pre-primary education. *International Journal of Behavioral Development*.

World Health Organization. (2010). *Indicators for assessing infant and young child feeding practices. Part 2 Measurement*. Geneva: WHO.
